# Long-term development of refractive error in refractive, nonrefractive and partially accommodative esotropia

**DOI:** 10.1371/journal.pone.0204396

**Published:** 2018-09-24

**Authors:** Paolo Esposito Veneruso, Dario Bruzzese, Adriano Magli

**Affiliations:** 1 GI.MA Eyecare Centre, Naples, Italy; 2 Department of Public Health, University of Naples “Federico II”, Naples, Italy; 3 Department of Pediatric Ophthalmology, University of Salerno, Salerno, Italy; Faculty of Medicine, Cairo University, EGYPT

## Abstract

Accommodative esotropia (AE) is a convergent deviation due to an excess of the convergence linked to the innervational stimulus for accommodation and it is usually associated to the presence of hyperopia. The development of hyperopia over time has been widely described but the lack of comparative analysis among accommodative esotropia subtypes, does not allow to verify the existence of different developmental patterns. In this study we aimed to describe the long term changes of refractive error in patients affected by accommodative esotropia as a function of the strabismus type: refractive (RAE), non-refractive (NRAE) and partially accommodative esotropia (PAE). The medical records of 66 patients (24 RAE, 22 PAE, 20 NRAE), who wore the full correction of their cycloplegic refraction error during the entire follow up period, were retrospectively reviewed. Mean outcome was the analysis of the variations, among groups, of refractive error over time; differences between mild (≤3.00D) and high (≥5.00D) hyperopia, effects of amblyopia and relationship with AC/A ratio were also investigated. All patients were followed up from approximately 4 years of age to 20, with mean follow up of 16.06±0.29 years. Our results described similar non-linear trend of refractive error development among groups. The initial increase of hyperopia (up to 7–8 years) was followed by a decreasing trend persisting up to adulthood (approximately 20 years of age). During this period, spherical equivalent decreased at a lower mean annual rate in patients affected by RAE (-0.07D) compared to other groups (NRAE -0.11D, p = 0.003 and PAE 0.13D, p = 0.002). In all groups, however, significant amount of hyperopia was found at last examination; indeed the observed difference in SE values from baseline to the end of follow up, was not significant (RAE: +0.27D, 95% C.I. -0.49 to +1.04D, p = 0.472; PAE: -0.69D, 95% C.I. -1.67 to +0.28D, p = 0.154; NRAE: -0.39D, 95% C.I. -1.11 to +0.34D, p = 0.278). AC/A ratio, amblyopia and amount of initial hyperopia appeared to have no significant role in longitudinal change of the refractive error. In conclusion, this study provides a complete overview, from childhood to adulthood, of refractive error development in different form of accommodative esotropia.

## Introduction

The term “accommodative esotropia” (AE) is generally referred to a convergent deviation due to an excess of the convergence linked to the innervational stimulus for accommodation [1]. It can be associated with hyperopia (frequently undercorrected at early age), anisometropia, insufficient fusional divergence, normal or high accommodative convergence to accommodation ratio (AC/A) and subnormal amplitude of accommodation [1–3].

Several studies, focused on the development pattern of refractive error in esotropic children, have reported an increasing or unchanged hyperopia from 5 to 10 years of age [4–7] and, afterwards, a decreasing trend is frequently observed [4,7,8].

The emmetropization mechanisms in esotropic patients and the effects of optical correction on the eye-growth remain, although extensively studied, an unresolved issue. Birch et al. reported a slower emmetropization process in esotropic children compared to healthy controls [9,10]; although some Authors suggested the emmetropization process might be prevented by using full optical correction of hyperopia [11,12], Demirkilinç Biler et al. described no significant differences between the development of refractive error in children treated with partial and full spectacles correction [13].

Despite contrasting result in human, in animal model, the effect of defocus induced by optical correction on eye growth is well documented [14–16].

The effects of amblyopia, anisometropia, the amount of initial hyperopia, age of prescription and full versus partial optical correction on the refractive change over time in AE patients, have been described with non-univocal opinions [7,8,13,17], moreover comparison of AE studies is very difficult because inclusion criteria and follow up lasting vary among studies and strabismus classification is not always clearly defined.

To the best of our knowledge, there are no studies focused on the possible differences of refractive error modifications over time among AE subtypes.

The aim of this study is to evaluate the long term changes of refractive error in patients affected by accommodative esotropia as a function of the strabismus type: refractive, non-refractive and partially accommodative esotropia.

## Materials and methods

Medical records of patient with accommodative esotropia examined between 1993 and 2015 at GI.MA Eyecare Center, followed up for the whole period by the same equipe (1 Ophthalmologist and 2 Orthoptist), were retrospectively reviewed. We selected patients affected by refractive accommodative esotropia (RAE), nonrefractive accommodative esotropia (NRAE) and partially accommodative esotropia (PAE).

According to von Noorden classification [2], accommodative esotropia was defined on the basis of the following clinical characteristics:

■ RAE: no significant distance-near incomitance (<6 prism diopters—pd) without optical correction, normal AC/A ratio and orthotropia or residual phoria (<10pd) at distance and near wearing glasses;
■ NRAE: significant distance-near incomitance (>10pd) without optical correction, high AC/A ratio, residual phoria or orthotropia wearing glasses at distance, residual phoria (<10pd) or orthotropia at near wearing a positive addiction (from 2.00 up to 3.00D) at near;
■ PAE: no significant distance-near incomitance (<6pd), significant residual deviation after optical correction at distance and near, normal AC/A ratio. All PAE patients underwent strabismus surgery.

All patients underwent extensive ophthalmological and orthoptic evaluation including best corrected visual acuity (BCVA) (Early Treatment Diabetic Retinopathy Study–ETDRS letters or symbol charts, according to patients’ cooperation), slit-lamp biomicroscopy, intraocular pressure and fundus oculi. Cycloplegic refraction was assessed by using retinoscopy. Cycloplegia was obtained with 1% Cyclopentolate + 1% Tropicamide every 15 minutes for 3 times at each examination during follow up period. Orthoptic evaluation included Hirschberg and Krimsky tests, cover, cover-uncover and alternating cover test at distance and near using an accommodative target, Worth’s 4 light test at distance and near, Lang II and TNO stereotest. AC/A ratio was measured by using the gradient method at distance adding -3.00 lens; AC/A ratio >5 was defined as high. Deviation values were obtained using prismatic alternating cover test at distance an near (6 and 0.3m).

In order to identify patients with nonaccommodative convergence excess (hyperkinetic type), all NRAE patients were treated with bifocals for 3 months at initial examination; significant change in the amount of deviation at near after this period, allowed us to select exclusively hypoaccommodative form of convergence excess esotropia. All NRAE patients were treated with bifocals; reduction or weaning exclusively from positive addition at near in NRAE group were established during follow up on the basis of patients’ ability to control near deviation; it was prescribed when residual phoria or good compensation of intermittent tropia at near were present.

All patients received full correction of their refractive error evaluated by retinoscopy for the entire period of follow up. The spherical equivalent (SE) was calculated as the sphere plus half a cylinder. SE variation >0.50D was considered significant.

Amblyopia was defined as an inter-eye difference in best corrected visual acuity of 2 or more logarithm of the minimal angle of resolution (logMAR) lines.

Occlusion therapy was performed from 2 to 4 hours in all amblyopic eyes according to the age of patient and depth of the amblyopia.

In order to rule out, as far as possible, any factor influencing the development of refractive error in esotropic children, strict inclusion criteria were applied:

- Follow up period of 16 years (from 4 to 20 years of age, approximately); at least 1 examination per year
- Baseline hyperopia (spherical equivalent) ≥1.50D
- Refractive, nonrefractive and partially accommodative esotropia (see above)
- Constant use of spectacles; good visual outcome (≥0.0 logMAR in non-amblyopic eyes).

Other forms of strabismus, lack of any follow up examination, discontinuous use of the optical correction, reduction of distance correction over time, systemic, metabolic or neurologic disease, developmental delay, ocular disease (corneal, optic media, retinal or optic nerve disease), strabismus surgery before age 4 and other previous surgery (nystagmus, ptosis) were considered conditions for exclusion from the study.

Mean outcome was the analysis of the variations, among groups, of refractive error over time; differences between mild and high hyperopia, effects of amblyopia and relationship with AC/A ratio were also investigated.

The research adhered to the tenets of the Declaration of Helsinki and was approved by the Institutional Review Board of the University of Salerno. Written informed consent was obtained from all subjects, or their parents, after full explanation of the aims and modalities of the investigation.

### Statistical analysis

Demographic and clinical data referred to the baseline visit were summarized using standard descriptive statistics, mean±standard deviation in case of numerical variables and frequencies and percentages in case of qualitative factors. Accordingly, comparison among groups were based on ANOVA test or the chi square test.

Longitudinal trajectories of SE and Cylinder have been analyzed using linear mixed models (LMM) with subject specific random term for the intercept. Time was treated as categorical factors to account for non-linear relationship. Interaction between time and groups were assessed by adding the corresponding interaction term in the model. All the results of the LMM are expressed as estimated marginal means with the corresponding 95% Confidence Intervals (95% C.I.). Moreover, after observing a non-linear trend in the longitudinal trajectories of spherical equivalent, a further analysis was done by dividing the whole follow-up in two different periods and by computing, for each of these, a LMM were time was coded continuously. This allowed to estimate a mean annual change of spherical equivalent for each period and for each group.

Differences between amblyopic and fellow eyes, in terms of SE, were analyzed by using the T test for paired sample.

All tests were two sided and statistical significance was set at p<0.05. Statistical analysis was performed using R statistical computing software (R Foundation for Statistical Computing, Vienna, Austria).

## Results

A total of 66 subjects affected by accommodative esotropia met the inclusion criteria. Patients were assigned to RAE (n = 24), PAE (n = 22) or NRAE (n = 20) group according to strabismus characteristics as previously described (see Materials and Methods section).

In the overall cohort, mean (± std. dev.) age at first prescription was 4.4±0.3 years (range: 4 to 4.9 years) and mean follow up period was 16.1±0.3 years (range: 15.4 to 16.7 years), up to 20.4±0.2 years of age (range: 20.0 to 20.9 years). No significant differences were observed among the three groups in terms of age at first prescription (p = 0.360). The distribution of gender was also homogenous among the three groups (p = 0.260). Demographic and clinical data are shown in Table 1.

**Table 1:** Demographic and clinical data (left eyes data are shown).

		*initial SE*		*8 years of age SE*		*final SE*	
	*gender (female)*	*mean age±sd*	*mean SE±sd [max; min]*	*mean age±sd*	*mean SE±sd [max; min]*	*mean age±sd*	*mean SE±sd [max; min]*
***RAE (n = 24)***	*13*	*4*.*3±0*.*2*	**5±2 [8.88; 1.63]**	*8*.*4±0*.*3*	**6.03±2 [10.38; 2.13]**	*20*.*5±0*.*2*	**5.25±2.1 [10; 1.63]**
***PAE (n = 22)***	*9*	*4*.*4±0*.*3*	**4.48±1.5 [7.50; 1.75]**	*8*.*5±0*.*3*	**4.94±1.7 [8.50; 2.25]**	*20*.*5±0*.*2*	**3.78±2.2 [7.38; 0]**
***NRAE (n = 20)***	*11*	*4*.*4±0*.*3*	**4.11±1.8 [7.13; 1.50]**	*8*.*4±0*.*2*	**4.81±1.8 [8; 1.25]**	*20*.*4±0*.*2*	**3.70±2.3 [7.13; -1]**
***amblyopic eyes (n = 18)***	*7*	*4*.*5±0*.*3*	**4.78±1.8 [9; 2.25]**	*8*.*4±0*.*3*	**5.15±1.8 [8; 2.38]**	*20*.*4±0*.*2*	**3.10±2.1 [6.50; -0.75]**

**SE:** spherical equivalent

**RAE:** refractive accommodative esotropia

**PAE:** partially accommodative esotropia

**NRAE:** non-refractive accommodative esotropia

sd: standard deviation

### Spherical equivalent

According to LMMs, mean (95% CI.) SE values at baseline were in the right eye 4.96D (4.19 to 5.73D), 4.58D (3.78 to 5.38D), 4.01D (3.12 to 4.8D) and in the left eye 5D (4.2 to 5.76D), 4.48D (3.67 to 5.29D), 4.11D (3.24 to 4.94D) in RAE, PAE and NRAE groups respectively. For both right and left eyes no significant differences were found among groups (right eye: p = 0.086 in the NRAE vs. RAE comparison, p = 0.501 in the PAE vs. RAE comparison and p = 0.294 in the PAE vs. NRAE comparison; left eye p = 0.128 in the NRAE vs. RAE comparison, p = 0.377 in the PAE vs. RAE comparison and p = 0.512 in the PAE vs. NRAE comparison).

At last examination only 2 patients (1 PAE and 1 NRAE—2.9% of our sample) were emmetropes and only 1 NRAE (1.5%) patient showed a myopic refraction (SE -0.75D); except for those 3 patients, all subjects showed a residual hyperopic refraction. Mean SE values at last examination, with the corresponding 95% C.I., were in the right eye 5.01D (4.24 to 5.77D), 3.71D (2.91 to 4.51D), 3.59D (2.75 to 4.43D) and in the left eye 5.25D (4.47 to 6.03D), 3.78D (2.97 to 4.6D), 3.7D (2.85 to 4.55D) in RAE, PAE and NRAE groups respectively. For both right and left eyes, RAE residual SE mean values were significantly higher than PAE (p = 0.023 and p = 0.011 in the right and left eyes, respectively) and NRAE ones (p = 0.015 and p = 0.009 in the right and left eyes, respectively). No significant differences between PAE and NRAE values were found (p = 0.834 and p = 0.887 in the right and left eyes, respectively).

**Fig 1:**
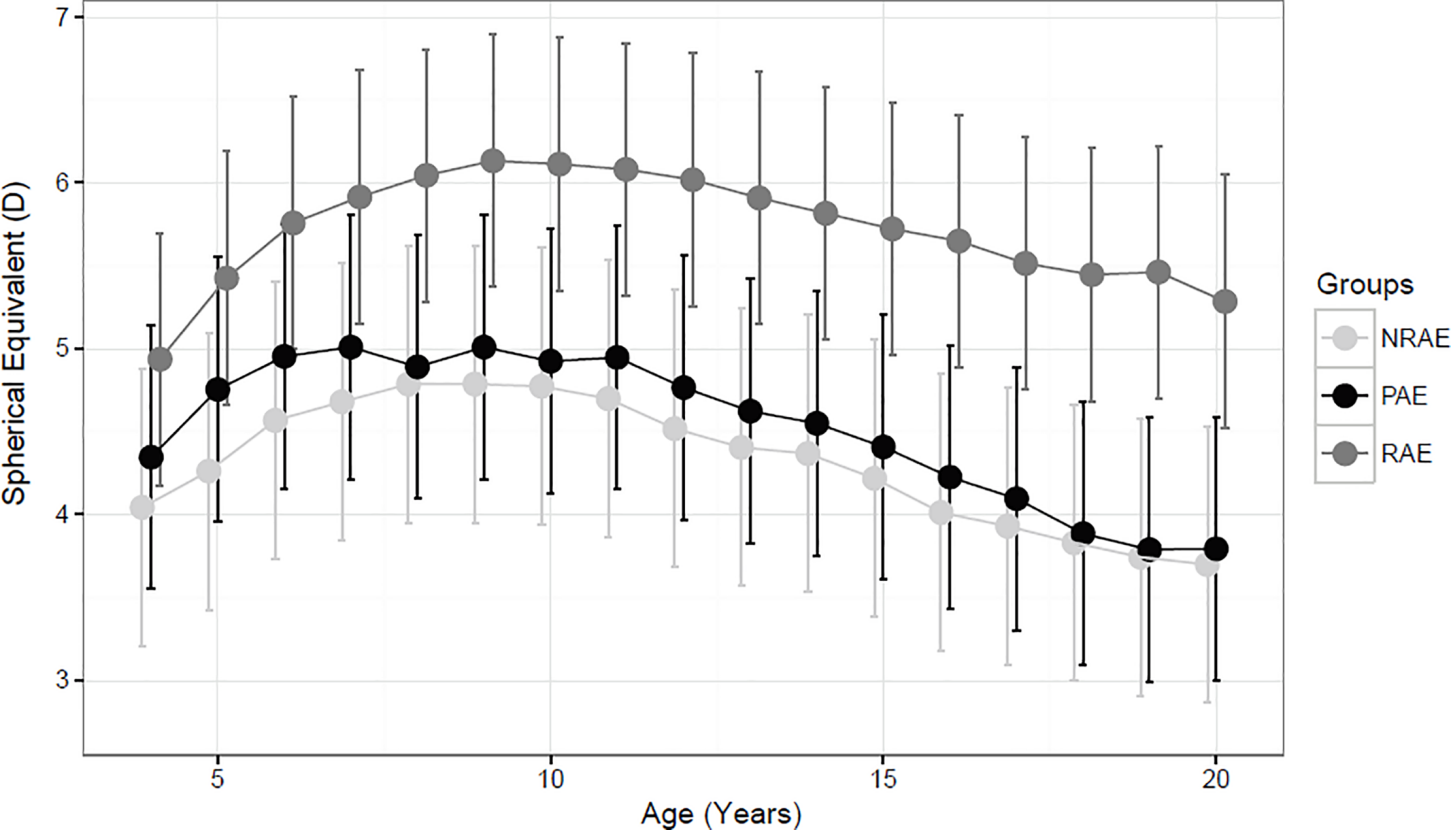
Estimated Marginal Means, with 95% C.I., of refractive error over time in refractive, nonrefractive and partially accommodative esotropia patients.

Fig 1 shows the change in SE mean values from age 4 to 20 for all groups; left eyes were considered. In all groups, an increasing trend of hyperopia from the first prescription up to approximately 7 years of age was found; this period was slightly prolonged (up to 8 years of age) in RAE subjects compared to NRAE and PAE ones. After 3–4 years from first prescription (7–8 years of age) all groups showed a consistent reduction of the SE up to 20 years of age. Due to this nonlinear trend, we analyzed separately the first 4 years and the following 13 years of follow up.

By observing, from a descriptive point of view, the subject specific trajectories over time, we observed that the patients with an increasing trend of the refractive error during the first years from the first prescription were 17 (70.8%) in RAE, 15 (68.2%) in PAE and 13 (65%) in NRAE group. Six (25%), 4 (18.2%) and 6 (30%) patients of RAE, PAE and NRAE group respectively showed no significant changes in the same follow up period and 4 patients (1 RAE, 1 NRAE and 3 PAE) showed an SE reduction trend.

The estimated mean annual increment, in the first four years of follow up, was equal to +0.31D (95% C.I.: 0.22 to 0.4D, p<0.001), +0.19D (95% C.I.: 0.1 to 0.29, p<0.001) and +0.22D (95% C.I.: 0.12 to 0.32D, p<0.001) in RAE, PAE and NRAE respectively, with no significant differences among groups.

During the following years, a significant mean annual reduction was observed in NRAE (-0.11D, 95% C.I.: -0.13 to -0.09D,p<0.001), PAE (-0.12D, 95% C.I. -0.13 to -0.1D,P<0.001) and RAE patients (-0.07D, 95% C.I.: -0.09 to -0.06D, p<0.001). In particular, the decrease rate observed in the RAE patients was significantly less pronounced than that observed in NRAE (p = 0.001) and PAE (p<0.001) patients.

Howewer, when considering the entire follow-up, no groups showed a significant variation in their refractive error (RAE: +0.27D, 95% C.I. -0.49 to +1.04D, p = 0.472; PAE: -0.69D, 95% C.I. -1.67 to +0.28D, p = 0.154; NRAE: -0.39D, 95% C.I. -1.11 to +0.34D, p = 0.278)

### Astigmatism

**Fig 2:**
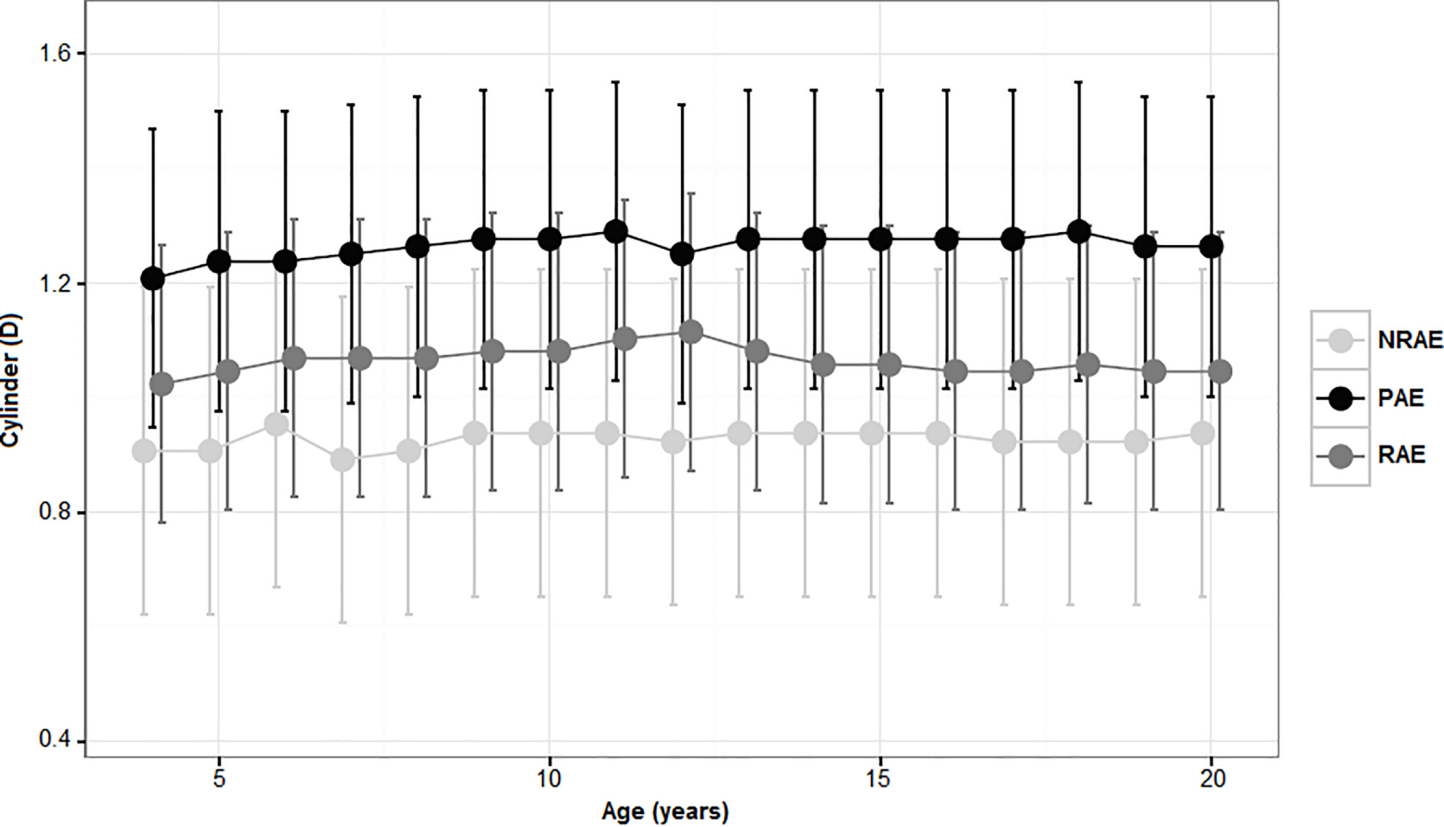
Estimated Marginal Means, with 95% C.I., of astigmatism over time in refractive, nonrefractive and partially accommodative esotropia patients. Data refer to left eyes.

Mean baseline values of cylinder (left eye), with the corresponding 95% C.I., were 1.02D (0.78 to 1.27 D), 0.91D (0.62 to 1.19D), 1.21D (0.95 to 1.47D) in RAE, NRAE and PAE groups respectively, without clinical significant modification over timer. At last examination mean cylinder values were 1.05D (0.80 to 1.29 D), 0.94 D (0.65 to 1.22D), 1.26D (1.00 to 1.52D) in RAE, NRAE and PAE respectively. Fig 2 show astigmatism trend over time. No statistically significant difference among group were found (S1 Table).

### AC/A ratio

AC/A ratio of RAE and PAE group was normal (<5). NRAE patients AC/A ratio mean value was 7.22±1.57 (ranging from 5.5 to 10).

In order to study whether higher or lower AC/A ratio values might have influenced the longitudinal change of SE values in NRAE patients, they were arbitrarily divided into 2 subgroups ≥7 and <7, on the basis of their AC/A ratio value. Ten (50%) patient showed an AC/A ratio ranging from 5.5 and 6.5 and 10 (50%) patient from 7 to 10. Baseline mean SE, with the corresponding 95% C.I., were 4.59D (3.41 to 5.76D) in <7 group and 3.59D (2.41 to 4.76D) in ≥7 group (p = 0.226). No significant differences were found at any time point during follow. In the last examination, although mean SE values were smaller in ≥ 7 group (2.89D, 95% C.I.: 1.71 to 4.06) compared to those of <7 group (4.51D 95% C.I.: 3.34 to 5.69D), this difference did not reach the statistical significance (p = 0.055).

### Amblyopia

**Fig 3:**
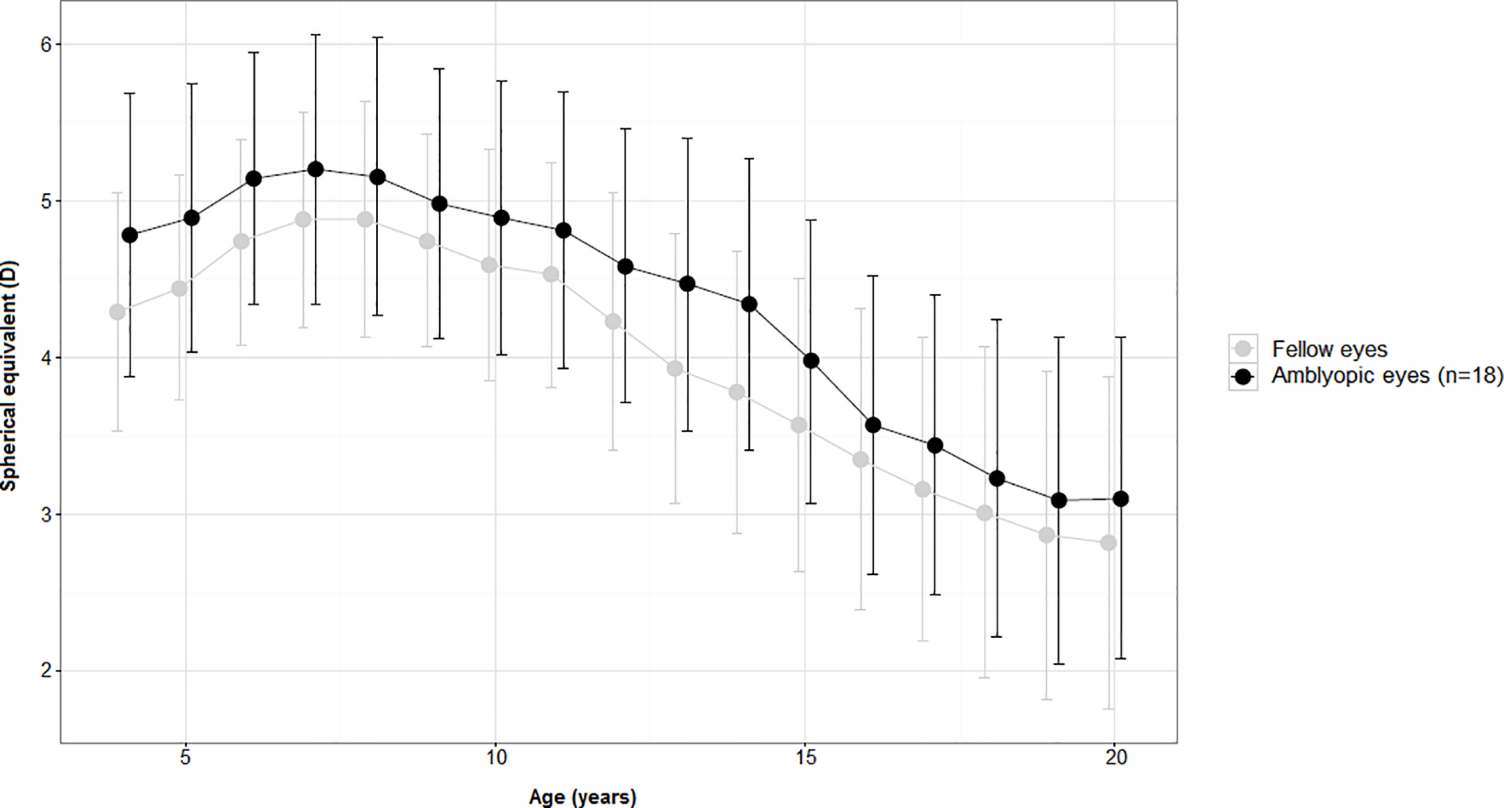
Mean values (95% C.I.) of refractive error over time of amblyopic and fellow eyes in PAE and NRAE subjects (n = 18).

**Fig 4:**
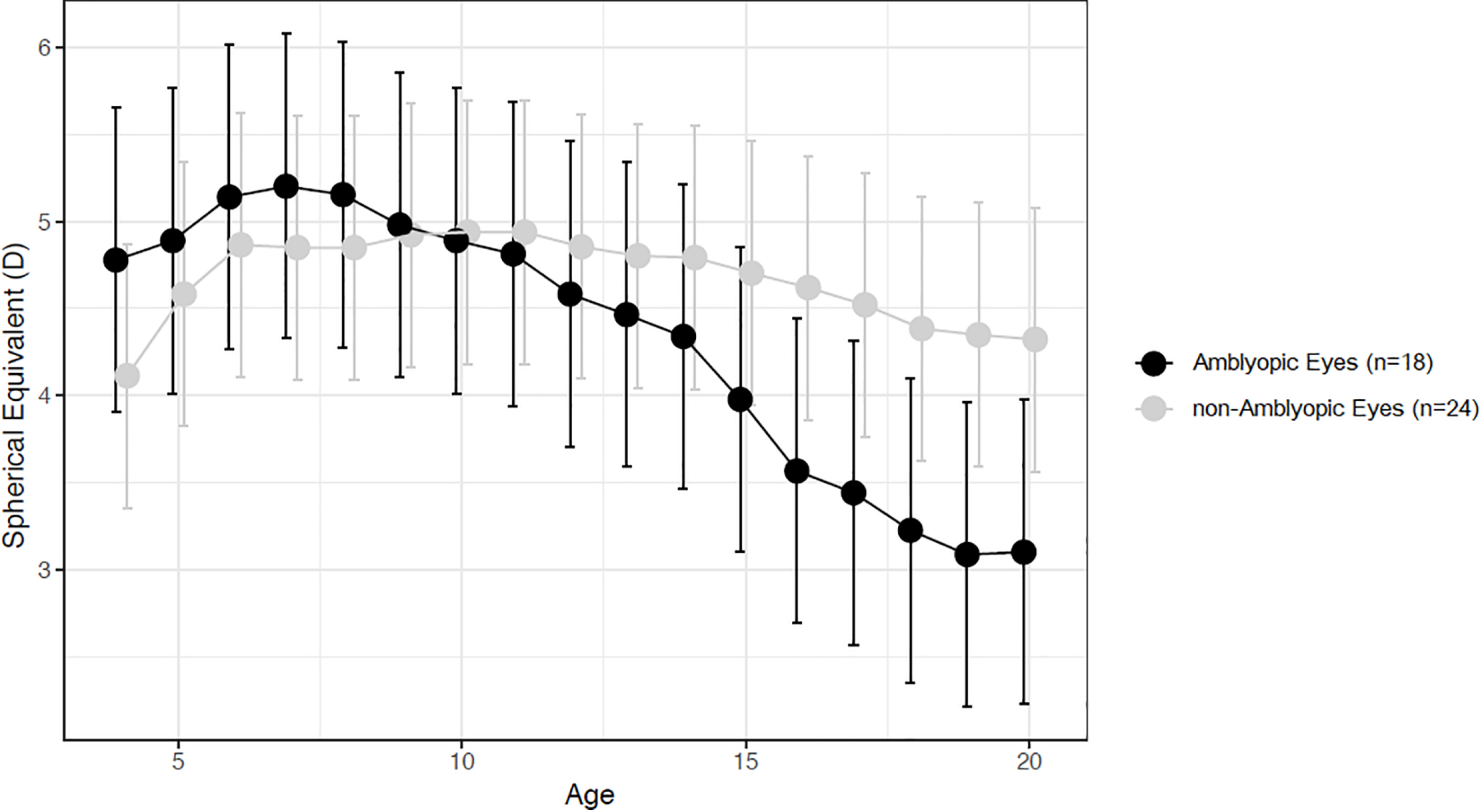
Estimated Marginal Means, with 95% C.I., of refractive error over time of amblyopic eyes (n = 18) and left eyes of non-amblyopic subjects (n = 24) in PAE and NRAE groups.

Eighteen (13 PAE and 5 NRAE) subjects showed amblyopia (14 strabismic and 4 anisometropic amblyopia) and performed occlusion therapy at early age. No patients of RAE group showed amblyopia. Occlusion therapy was performed on the basis of depth of amblyopia from 2 to 4 hours per day up to 10 years of age. At the end of treatment no patients showed residual amblyopia (BCVA ≥0.0 logMAR). No significant differences were noticed when comparing SE mean values of amblyopic and fellow eyes (Fig 3; S2 Table). When mean SE values of the amblyopic eyes (n = 18) and non-amblyopic eyes of NRAE/PAE patients (n = 24) were compared, no significant differences were observed in each time points (Fig 4; S3 Table). However, when the 8–20 years period was analyzed separately, the amblyopic eye SE showed a significantly (p<0.001) steeper decline (-0.19D, 95% C.I. -0.21 to -0.18D) compared to non-amblyopic ones (-0.06D, 95% C.I. -0.07 to -0.04D).

### Mild versus high hyperopia

**Fig 5:**
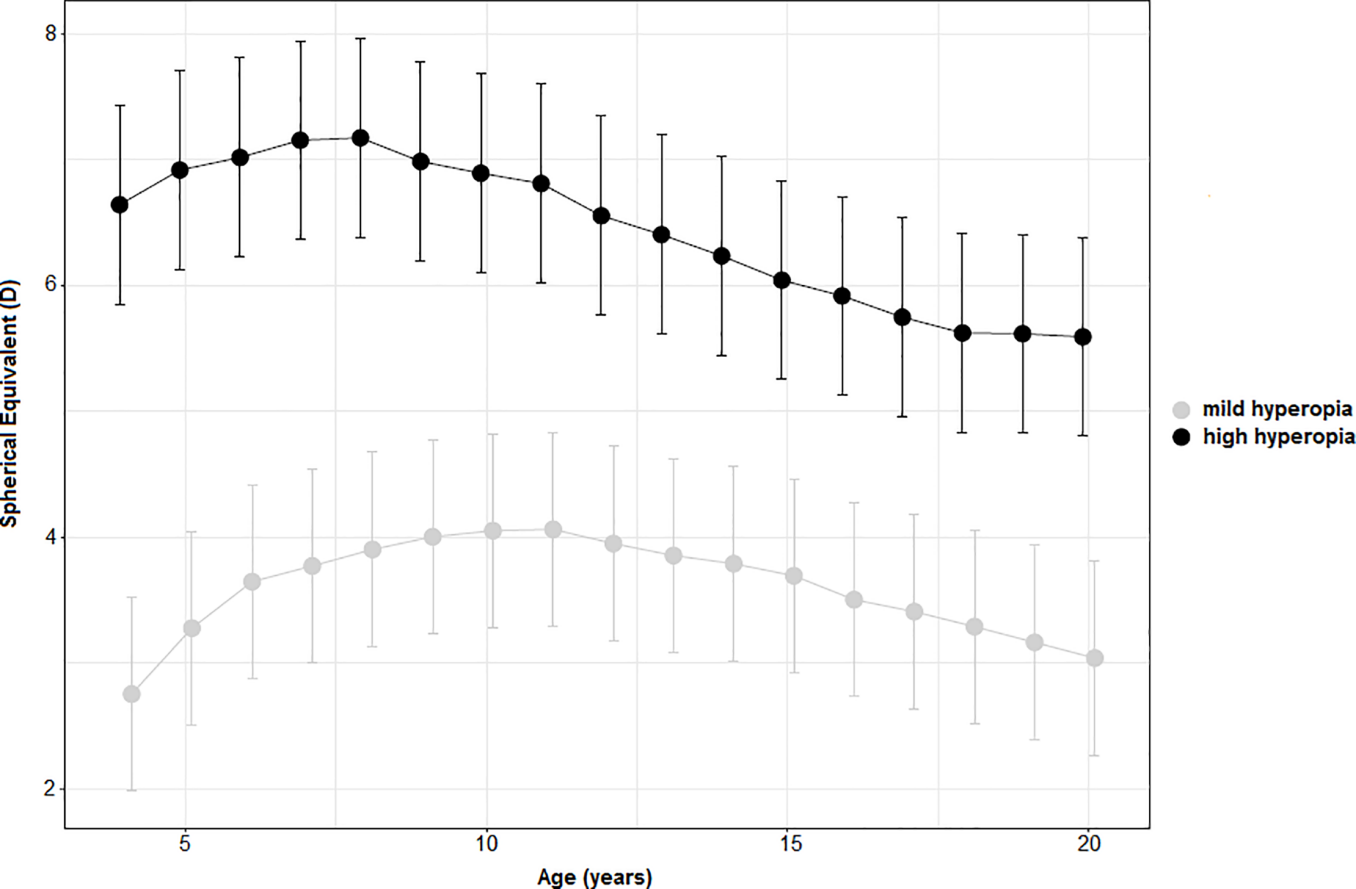
Estimated Marginal Means, with 95% C.I., of refractive error over time of mild (≤3.00D) and high (≥5.00D) hyperopia groups.

In order to study the differences of SE developmental pattern on the basis of the baseline amount of SE all patients were divided in high (≥5.00D) and mild (≤3.00D) hyperopia groups. SE baseline mean values were 2.76D (95% C.I.: 1.99 to 3.53D;) and 6.64D (95% C.I.: 5.85 to 7.43D). Fig 5 shows similar non-linear trend in both groups; mild group appears to reach the SE peak between 8 and 10 years of age, high group between 7–8 years of age. After reaching the apex, both group showed a constant reduction of SE up to 20.5 years, approximately. Considering the entire follow up period, patients in the high hyperopia group experienced a reduction of SE (-1.05±1.89D) significantly higher than that observed in the mild hyperopia group (+0.28 ±1.83D; p = 0.027).

## Discussion

Although previous studies described the longitudinal changes of refractive error in accommodative esotropia [1,5–7,12,13,17], the differences of developmental pattern of refractive error among accommodative esotropia subtypes were scarcely investigated. The goal of this study was to evaluate the development of the refractive error as function of the type of accommodative esotropia: refractive, nonrefractive and partially accommodative esotropia. In order to rule out, as far as possible, any factor influencing the development of refractive error (i.e. different age at first prescription or different follow up), patients were selected on the basis of strict inclusion criteria.

According to Repka et al. and Raab, an initial increasing trend of the spherical equivalent up to 7 years of age were found with no statistically significant differences among groups (mean annual change ranged from 0.1 to 0.4D) [1,6]. Lambert and Lynn found similar results and added that the timing of the SE peak was function of the age of glasses prescription; for patients who were prescribed glasses at early age, hyperopia peaked slower (meanly 6 years after prescription) compared to patients who were prescribed glasses at a later age [17].

Nevertheless, It has to be considered that this initial increase of hyperopia might be influenced by gradual relaxation of the ciliary muscle tone probably in response to the effects of the optical correction [5] or by the hypothetical ineffectiveness of cycloplegia to completely damp ciliary muscular fibers activity at early age (age at baseline examination: RAE 4.3±0.2 years; PAE 4.4±0.3 years and NRAE 4.4±0.3 years). Fig 1 shows hyperopia in RAE patients peaked slightly later than PAE and NRAE patients (8 years of age).

After 7–8 years of age a decreasing trend of the SE was observed in all groups (mean annual change ranged from -0.05 to -0.13D). Whereas no differences of SE mean annual changes were found between PAE and NRAE groups, RAE patients showed a significantly lower reduction of refractive error (-0.07D; 95% C.I. -0.05 to 0.09) compared with NRAE (p = 0.001) and PAE (p<0.001) patients.

Mean amount of decreasing per year (RAE -0.07D; PAE -0.12D; NRAE -0.11D) was comparable to those previously reported by other Authors [4,12,13]. By contrast smaller mean decrease per year was found by Wang et al. in the late-onset accommodative ET group (-0.012D/y). This difference may be due to the different amount of initial hyperopia and lasting of follow up period (from 4D to 10D vs >1.5D; 6.5±1.6 years follow up vs 16 years follow up).

Nevertheless, in this study, a significant amount of residual hyperopia was present in all groups (at least 2.50D) after 16 years from the first examination. This finding appears to be consistent with theories describing that full correction of hyperopic refractive error might impede emmetropization process in children. Several studies, indeed, described that eliminating retinal blur by wearing glasses may prevent emmetropization [18–21]. Repka et al. suggested that patients affected by accommodative esotropia who wear their full hyperopic correction appear to be less likely to go through the emmetropization process [6]. By contrast other studies reported that full and partial hyperopic correction have no significantly different effects on emmetropization [12,13].

Our results describe similar non-linear trend over time among refractive, nonrefractive and partially accommodative esotropia but a significantly slower reduction of spherical equivalent in refractive esotropia group is present after 7–8 years of age. Authors speculated that eliminating retinal blur in hyperopic children with appropriate lenses could remove the stimulus for the myopic shift toward emmetropia [22] or that an intrinsic defect in the process of emmetropization might exist in accommodative esotropes [21]. Considering that all enrolled patients in this study received full correction of their refractive error, these theories are not able to explain the slightly different behavior of RAE group compared to other included form of accommodative esotropia. The role of ocular alignment or motor control skill or different accommodation/convergence mechanisms of such different form of accommodative esotropia in the emmetropization process, might only be speculated. On the other hand, a possible bias related to the sample selection or size, cannot be excluded. This issue required further investigation.

In order to study the difference of refractive error development in mild and high hyperopia, our patients were divided in high (>+5.00D) and mild (<+3.00D) hyperopia groups on the basis of initial amount of hyperopia. Although Mezer et al. observed a greater reduction of refractive error in patients with mild hyperopia (<3.00D) compared to high hyperopia (>5.00D) ones [23], we found no statistically significant differences between groups over time.

Although previous studies [24,25] described a greater eye elongation of non-amblyopic eyes compared to amblyopic ones, we found different results. According to other studies [7,8,26], indeed, we reported no difference in SE change over time between amblyopic and fellow eyes but a significantly greater decrease of the amblyopic eyes SE (mean values: -0.19D) when compared to those of non-amblyopic subjects [7] (mean values: -0.06D). Parks proposed possible, reasonable explanations of this phenomenon based on differences of demographics characteristics, amount of initial hyperopia, genetics and environmental factors and the role of blurred vision induced by amblyopia. Nevin et al. experimentally induced myopia in chicks using plus lenses and applied stand-off cones to interfere with sharp vision. These authors found that the preclusion of sharp vision not only prevented compensation but also resulted in increased eye growth and myopia in the chicks [27]. It is reasonable to hypothesize that, similarly, the lack of sharp vision induced by amblyopia might alter the effect of glasses and the development of refractive error.

One of most discussed environmental factor concerning the myopic shift in children is the effects of the balance near/outdoor activities [28,29]. Severe and prolonged near activity with moderate outdoor activity, and the inappropriate eye distance for near prolonged work without eye break, appear to be risk factors for myopic shifts. These effects might have influenced the “natural” development of refractive error in our cohort of hyperopic patients but the retrospective nature of our study does not allow us to take them in consideration for the statistical analysis.

For the first time, to the best of our knowledge, the effects of AC/A ratio on the development of refractive error in patients affected by accommodative esotropia were investigated. Patients with non-refractive accommodative were arbitrarily divided in lower (<7) and higher (≥7) AC/A ratio (on the basis of ratio measurement made between 4 and 6 years of age) and the variations of refractive error over time were compared between these groups. Although slightly significant differences were found at 13,14,15 and 20 years of age, the statistical significance level (p = 0.036, 0.042, 0.047 and 0.037 respectively) and the sample size let us to consider the AC/A ratio as no clinically relevant in regards of the development of refractive error in patients affected by accommodative esotropia.

This study presents several limitations: 1) our sample size is small and, in order to confirm our results, further multicentric studies are required; 2) the lack of adequate number of patients affected by anisometropic and strabismic amblyopia for a comparative analysis and the evaluation of the effects of treatment (occlusion and penalization) on the refractive error development; 3) the intrinsic bias related to the retrospective design of the study, such as AC/A ratio missing data after 3 years of follow up; 4) the lack of control group including fully-corrected hyperopic ortotropic patients, patients weaned from spectacles and low hyperopic patients with no treatment; 5) the lack of ocular biometric data such as axial-length to provide an objective measure of the eye-growth.

In conclusion this represent, to the best of our knowledge, the longest follow up analysis of refractive error development in accommodative esotropia, and probably the first one providing a complete overview, from childhood to adulthood, of the hyperopia modification pattern focused on differences among different accommodative esotropia subtypes: refractive, nonrefractive and partially accommodative. Our results described similar non-linear trend of refractive error development among groups. The initial increase of hyperopia (up to 7–8 years) was followed by a decreasing trend persisting up to adulthood (approximately 20 years of age). Patients affected by refractive accommodative esotropia showed a significantly lower reduction of spherical equivalent compared to other groups. However, significant amount of hyperopia was found in all groups at last examination. AC/A ratio, amblyopia and amount of initial hyperopia appeared to have no significant role in longitudinal change of the refractive error.

Although further wider studies are required, this study allow to enhance the comprehension of the long term development pattern of refractive error in esotropic patients, as a function of their form of strabismus. Our results, combined to those present in literature, could provide reference for clinicians to describe longitudinal prognosis of spectacle correction for patients with different form of accommodative ET and, therefore, to choice the most appropriate approach for the clinical management of these patients.

## Supporting information

S1 Table Estimated Marginal Means, with 95% C.I., of astigmatism over time in refractive, nonrefractive and partially accommodative esotropia patients. Data refer to left eyes.
(DOCX)

S2 Table Refractive error over time of amblyopic and fellow eyes in PAE and NRAE subjects (n = 18). (DOCX)

S3 Table Estimated Marginal Means, with 95% C.I., of refractive error over time of amblyopic eyes (n = 18) and left eyes of non-amblyopic subjects (n = 24) in PAE and NRAE groups. (DOCX)

S4 Table Patients’ raw clinical data. (DOCX)

S5 Table NRAE patients’ A/CA ratio data. (DOCX)
