# Supplementary material for: Long-term development of refractive error in refractive, nonrefractive and partially accommodative esotropia
Source: PLoS One. 2018 Sep 24;13(9):e0204396. doi: 10.1371/journal.pone.0204396 (PMC6152953; doi:10.1371/journal.pone.0204396)
Supplement: S1 Table — Data refer to left eyes. (DOCX) [file pone.0204396.s001.docx]

**S1 Table.**

|  | **Cylinder (D)** | | | **pvalue** | | |
| --- | --- | --- | --- | --- | --- | --- |
| **Age (years)** | **NRAE** | **PAE** | **RAE** | **NRAE vs. RAE** | **PAE vs. RAE** | **PAE vs. NRAE** |
| 4 | 0.91 [0.62 to 1.19] | 1.21 [0.95 to 1.47] | 1.02 [0.78 to 1.27] | 0,566 | 0,305 | 0,124 |
| 5 | 0.91 [0.62 to 1.19] | 1.24 [0.98 to 1.5] | 1.05 [0.8 to 1.29] | 0,459 | 0,287 | 0,092 |
| 6 | 0.95 [0.67 to 1.24] | 1.24 [0.98 to 1.5] | 1.07 [0.83 to 1.31] | 0,541 | 0,348 | 0,147 |
| 7 | 0.89 [0.61 to 1.18] | 1.25 [0.99 to 1.51] | 1.07 [0.83 to 1.31] | 0,346 | 0,312 | 0,068 |
| 8 | 0.91 [0.62 to 1.19] | 1.26 [1 to 1.52] | 1.07 [0.83 to 1.31] | 0,39 | 0,278 | 0,069 |
| 9 | 0.94 [0.65 to 1.22] | 1.28 [1.02 to 1.54] | 1.08 [0.84 to 1.32] | 0,45 | 0,274 | 0,084 |
| 10 | 0.94 [0.65 to 1.22] | 1.28 [1.02 to 1.54] | 1.08 [0.84 to 1.32] | 0,45 | 0,274 | 0,084 |
| 11 | 0.94 [0.65 to 1.22] | 1.29 [1.03 to 1.55] | 1.1 [0.86 to 1.35] | 0,382 | 0,298 | 0,073 |
| 12 | 0.92 [0.64 to 1.21] | 1.25 [0.99 to 1.51] | 1.11 [0.87 to 1.36] | 0,309 | 0,447 | 0,094 |
| 13 | 0.94 [0.65 to 1.22] | 1.28 [1.02 to 1.54] | 1.08 [0.84 to 1.32] | 0,45 | 0,274 | 0,084 |
| 14 | 0.94 [0.65 to 1.22] | 1.28 [1.02 to 1.54] | 1.06 [0.81 to 1.3] | 0,526 | 0,223 | 0,084 |
| 15 | 0.94 [0.65 to 1.22] | 1.28 [1.02 to 1.54] | 1.06 [0.81 to 1.3] | 0,526 | 0,223 | 0,084 |
| 16 | 0.94 [0.65 to 1.22] | 1.28 [1.02 to 1.54] | 1.05 [0.8 to 1.29] | 0,566 | 0,2 | 0,084 |
| 17 | 0.92 [0.64 to 1.21] | 1.28 [1.02 to 1.54] | 1.05 [0.8 to 1.29] | 0,511 | 0,2 | 0,071 |
| 18 | 0.92 [0.64 to 1.21] | 1.29 [1.03 to 1.55] | 1.06 [0.81 to 1.3] | 0,473 | 0,197 | 0,062 |
| 19 | 0.92 [0.64 to 1.21] | 1.26 [1 to 1.52] | 1.05 [0.8 to 1.29] | 0,511 | 0,227 | 0,082 |
| 20 | 0.94 [0.65 to 1.22] | 1.26 [1 to 1.52] | 1.05 [0.8 to 1.29] | 0,566 | 0,227 | 0,097 |
