# Supplementary material for: Long-term development of refractive error in refractive, nonrefractive and partially accommodative esotropia
Source: PLoS One. 2018 Sep 24;13(9):e0204396. doi: 10.1371/journal.pone.0204396 (PMC6152953; doi:10.1371/journal.pone.0204396)
Supplement: S2 Table — (DOCX) [file pone.0204396.s002.docx]

**S2 Table.**

| Age | Fellow Eyes | Amblyopic Eyes | pvalue |
| --- | --- | --- | --- |
| 4 | 4.3 ± 1.5 | 4.8 ± 1.8 | 0,053 |
| 5 | 4.4 ± 1.4 | 4.9 ± 1.7 | 0,078 |
| 6 | 4.7 ± 1.3 | 5.1 ± 1.6 | 0,089 |
| 7 | 4.9 ± 1.4 | 5.2 ± 1.7 | 0,132 |
| 8 | 4.9 ± 1.5 | 5.2 ± 1.8 | 0,256 |
| 9 | 4.7 ± 1.4 | 5 ± 1.7 | 0,389 |
| 10 | 4.6 ± 1.5 | 4.9 ± 1.8 | 0,337 |
| 11 | 4.5 ± 1.4 | 4.8 ± 1.8 | 0,329 |
| 12 | 4.2 ± 1.7 | 4.6 ± 1.8 | 0,325 |
| 13 | 3.9 ± 1.7 | 4.5 ± 1.9 | 0,157 |
| 14 | 3.8 ± 1.8 | 4.3 ± 1.9 | 0,143 |
| 15 | 3.6 ± 1.9 | 4 ± 1.8 | 0,294 |
| 16 | 3.3 ± 1.9 | 3.6 ± 1.9 | 0,577 |
| 17 | 3.2 ± 1.9 | 3.4 ± 1.9 | 0,445 |
| 18 | 3 ± 2.1 | 3.2 ± 2 | 0,579 |
| 19 | 2.9 ± 2.1 | 3.1 ± 2.1 | 0,563 |
| 20 | 2.8 ± 2.1 | 3.1 ± 2.1 | 0,443 |
