# Supplementary material for: Long-term development of refractive error in refractive, nonrefractive and partially accommodative esotropia
Source: PLoS One. 2018 Sep 24;13(9):e0204396. doi: 10.1371/journal.pone.0204396 (PMC6152953; doi:10.1371/journal.pone.0204396)
Supplement: S3 Table — (DOCX) [file pone.0204396.s003.docx]

**S3 Table.**

| **Age (years)** | **Amblyopic Eyes (n=18)** | **non-Amblyopic Eyes (n=24)** | **pvalue** |
| --- | --- | --- | --- |
| 4 | 4.1 [3.4 to 4.9] | 4.8 [3.9 to 5.7] | 0,254 |
| 5 | 4.6 [3.8 to 5.3] | 4.9 [4 to 5.8] | 0,598 |
| 6 | 4.9 [4.1 to 5.6] | 5.1 [4.3 to 6] | 0,636 |
| 7 | 4.8 [4.1 to 5.6] | 5.2 [4.3 to 6.1] | 0,543 |
| 8 | 4.8 [4.1 to 5.6] | 5.2 [4.3 to 6] | 0,6 |
| 9 | 4.9 [4.2 to 5.7] | 5 [4.1 to 5.9] | 0,921 |
| 10 | 4.9 [4.2 to 5.7] | 4.9 [4 to 5.8] | 0,933 |
| 11 | 4.9 [4.2 to 5.7] | 4.8 [3.9 to 5.7] | 0,829 |
| 12 | 4.9 [4.1 to 5.6] | 4.6 [3.7 to 5.5] | 0,64 |
| 13 | 4.8 [4 to 5.6] | 4.5 [3.6 to 5.3] | 0,561 |
| 14 | 4.8 [4 to 5.5] | 4.3 [3.5 to 5.2] | 0,436 |
| 15 | 4.7 [3.9 to 5.5] | 4 [3.1 to 4.9] | 0,214 |
| 16 | 4.6 [3.9 to 5.4] | 3.6 [2.7 to 4.4] | 0,074 |
| 17 | 4.5 [3.8 to 5.3] | 3.4 [2.6 to 4.3] | 0,067 |
| 18 | 4.4 [3.6 to 5.1] | 3.2 [2.4 to 4.1] | 0,061 |
| 19 | 4.3 [3.6 to 5.1] | 3.1 [2.2 to 4] | 0,052 |
| 20 | 4.3 [3.6 to 5.1] | 3.1 [2.2 to 4] | 0,051 |
