# Supplementary material for: Long-term development of refractive error in refractive, nonrefractive and partially accommodative esotropia
Source: PLoS One. 2018 Sep 24;13(9):e0204396. doi: 10.1371/journal.pone.0204396 (PMC6152953; doi:10.1371/journal.pone.0204396)
Supplement: S4 Table — (DOCX) [file pone.0204396.s004.docx]

**S4 Table.**

| **subjects' #** | **time points** | **groups** | **sf (right eye)** | **cyl (right eye)** | **SE (right eye)** |  | **sf (left eye)** | **cyl (left eye)** | **SE (left eye)** |  |
| --- | --- | --- | --- | --- | --- | --- | --- | --- | --- | --- |
| 1 | settembre 1993 | **PAE** | 4,50 |  | **4,500** | * | 2,75 |  | **2,750** |  |
| 1 | novembre 1994 | **PAE** | 5,00 |  | **5,000** | * | 3,00 |  | **3,000** |  |
| 1 | luglio 1995 | **PAE** | 5,00 |  | **5,000** | * | 4,00 |  | **4,000** |  |
| 1 | luglio 1996 | **PAE** | 5,25 | 0,50 | **5,500** | * | 5,50 |  | **5,500** |  |
| 1 | dicembre 1997 | **PAE** | 5,25 | 0,50 | **5,500** | * | 5,50 |  | **5,500** |  |
| 1 | dicembre 1998 | **PAE** | 5,25 | 0,50 | **5,500** | * | 5,50 |  | **5,500** |  |
| 1 | dicembre 1999 | **PAE** | 5,75 | 0,25 | **5,875** | * | 5,50 |  | **5,500** |  |
| 1 | giugno 2000 | **PAE** | 5,50 | 0,75 | **5,875** | * | 5,50 |  | **5,500** |  |
| 1 | settembre 2001 | **PAE** | 5,50 | 0,75 | **5,875** | * | 5,50 |  | **5,500** |  |
| 1 | settembre 2002 | **PAE** | 5,50 | 0,75 | **5,875** | * | 5,50 |  | **5,500** |  |
| 1 | novembre 2003 | **PAE** | 5,50 | 0,75 | **5,875** | * | 5,00 |  | **5,000** |  |
| 1 | dicembre 2004 | **PAE** | 5,00 | 0,75 | **5,375** | * | 5,00 |  | **5,000** |  |
| 1 | dicembre 2005 | **PAE** | 4,00 | 0,50 | **4,250** | * | 4,75 |  | **4,750** |  |
| 1 | luglio 2006 | **PAE** | 4,00 | 0,50 | **4,250** | * | 4,75 |  | **4,750** |  |
| 1 | maggio 2007 | **PAE** | 4,00 | 0,50 | **4,250** | * | 4,75 |  | **4,750** |  |
| 1 | maggio 2008 | **PAE** | 4,00 | 0,50 | **4,250** | * | 4,75 |  | **4,750** |  |
| 1 | agosto 2009 | **PAE** | 4,00 | 0,50 | **4,250** | * | 4,75 |  | **4,750** |  |
| 2 | dicembre 1994 | **PAE** | 3,75 | 0,25 | **3,875** | * | 3,75 | 0,25 | **3,875** |  |
| 2 | luglio 1996 | **PAE** | 3,75 | 0,25 | **3,875** | * | 3,75 | 0,25 | **3,875** |  |
| 2 | luglio 1997 | **PAE** | 4,00 |  | **4,000** | * | 4,25 | 0,25 | **4,375** |  |
| 2 | luglio 1998 | **PAE** | 4,00 |  | **4,000** | * | 4,25 | 0,25 | **4,375** |  |
| 2 | settembre 1999 | **PAE** | 4,00 |  | **4,000** | * | 4,00 | 0,50 | **4,250** |  |
| 2 | giugno 2000 | **PAE** | 4,00 |  | **4,000** | * | 4,00 | 0,50 | **4,250** |  |
| 2 | marzo 2001 | **PAE** | 4,00 |  | **4,000** | * | 3,50 | 0,50 | **3,750** |  |
| 2 | maggio 2002 | **PAE** | 4,00 |  | **4,000** | * | 3,50 | 0,50 | **3,750** |  |
| 2 | febbraio 2003 | **PAE** | 3,75 |  | **3,750** | * | 3,50 | 0,50 | **3,750** |  |
| 2 | luglio 2004 | **PAE** | 2,75 |  | **2,750** | * | 2,75 | 0,50 | **3,000** |  |
| 2 | novembre 2004 | **PAE** | 2,75 |  | **2,750** | * | 2,75 | 0,50 | **3,000** |  |
| 2 | giugno 2006 | **PAE** | 2,75 |  | **2,750** | * | 2,75 | 0,50 | **3,000** |  |
| 2 | dicembre 2006 | **PAE** | 2,25 |  | **2,250** | * | 2,25 | 0,50 | **2,500** |  |
| 2 | dicembre 2007 | **PAE** | 2,25 |  | **2,250** | * | 2,25 | 0,50 | **2,500** |  |
| 2 | febbraio 2009 | **PAE** | 2,25 |  | **2,250** | * | 2,25 | 0,50 | **2,500** |  |
| 2 | dicembre 2009 | **PAE** | 1,75 |  | **1,750** | * | 1,75 | 0,50 | **2,000** |  |
| 2 | dicembre 2010 | **PAE** | 1,50 |  | **1,500** | * | 1,50 | 0,50 | **1,750** |  |
| 3 | agosto 2000 | **PAE** | 3,00 | 1,75 | **3,875** |  | 3,50 | 1,50 | **4,250** | * |
| 3 | dicembre 2001 | **PAE** | 2,50 | 1,75 | **3,375** |  | 3,00 | 1,50 | **3,750** | * |
| 3 | luglio 2002 | **PAE** | 2,50 | 1,75 | **3,375** |  | 3,00 | 1,50 | **3,750** | * |
| 3 | luglio 2003 | **PAE** | 2,25 | 1,75 | **3,125** |  | 2,00 | 1,50 | **2,750** | * |
| 3 | aprile 2004 | **PAE** | 2,00 | 1,75 | **2,875** |  | 2,00 | 1,50 | **2,750** | * |
| 3 | maggio 2005 | **PAE** | 2,00 | 1,75 | **2,875** |  | 2,00 | 1,50 | **2,750** | * |
| 3 | febbraio 2007 | **PAE** | 2,00 | 2,00 | **3,000** |  | 2,00 | 1,50 | **2,750** | * |
| 3 | giugno 2007 | **PAE** | 2,00 | 2,00 | **3,000** |  | 2,00 | 1,50 | **2,750** | * |
| 3 | ottobre 2008 | **PAE** | 2,00 | 2,00 | **3,000** |  | 2,00 | 1,50 | **2,750** | * |
| 3 | novembre 2009 | **PAE** | 2,00 | 2,00 | **3,000** |  | 2,00 | 1,50 | **2,750** | * |
| 3 | giugno 2010 | **PAE** | 1,50 | 2,00 | **2,500** |  | 1,75 | 1,50 | **2,500** | * |
| 3 | aprile 2011 | **PAE** | 1,50 | 2,00 | **2,500** |  | 1,75 | 1,50 | **2,500** | * |
| 3 | settembre 2012 | **PAE** | 1,50 | 2,00 | **2,500** |  | 1,75 | 1,50 | **2,500** | * |
| 3 | gennaio 2014 | **PAE** | 1,25 | 2,00 | **2,250** |  | 1,75 | 1,25 | **2,375** | * |
| 3 | giugno 2014 | **PAE** | 1,25 | 2,00 | **2,250** |  | 1,75 | 1,25 | **2,375** | * |
| 3 | aprile 2015 | **PAE** | 1,25 | 2,00 | **2,250** |  | 1,75 | 1,25 | **2,375** | * |
| 3 | settembre 2016 | **PAE** | 1,25 | 1,75 | **2,125** |  | 1,50 | 1,25 | **2,125** | * |
| 4 | ottobre 1996 | **RAE** | 7,00 | 1,75 | **7,875** |  | 6,50 | 2,00 | **7,500** |  |
| 4 | ottobre 1997 | **RAE** | 7,00 | 1,75 | **7,875** |  | 6,50 | 2,00 | **7,500** |  |
| 4 | luglio 1998 | **RAE** | 7,00 | 1,75 | **7,875** |  | 6,50 | 2,00 | **7,500** |  |
| 4 | marzo 1999 | **RAE** | 7,00 | 2,00 | **8,000** |  | 6,50 | 2,00 | **7,500** |  |
| 4 | febbraio 2000 | **RAE** | 7,00 | 2,00 | **8,000** |  | 6,50 | 2,00 | **7,500** |  |
| 4 | marzo 2001 | **RAE** | 7,00 | 2,00 | **8,000** |  | 6,50 | 2,00 | **7,500** |  |
| 4 | aprile 2002 | **RAE** | 7,00 | 2,00 | **8,000** |  | 6,50 | 2,00 | **7,500** |  |
| 4 | aprile 2003 | **RAE** | 6,50 | 2,00 | **7,500** |  | 5,50 | 2,25 | **6,625** |  |
| 4 | novembre 2004 | **RAE** | 6,50 | 2,00 | **7,500** |  | 5,50 | 2,25 | **6,625** |  |
| 4 | novembre 2005 | **RAE** | 6,50 | 2,00 | **7,500** |  | 5,50 | 2,25 | **6,625** |  |
| 4 | novembre 2006 | **RAE** | 6,50 | 2,25 | **7,625** |  | 5,50 | 2,25 | **6,625** |  |
| 4 | settembre 2007 | **RAE** | 6,50 | 2,25 | **7,625** |  | 5,50 | 2,25 | **6,625** |  |
| 4 | luglio 2008 | **RAE** | 6,50 | 2,25 | **7,625** |  | 5,50 | 2,25 | **6,625** |  |
| 4 | marzo 2009 | **RAE** | 6,50 | 2,25 | **7,625** |  | 5,00 | 2,25 | **6,125** |  |
| 4 | maggio 2010 | **RAE** | 6,50 | 2,00 | **7,500** |  | 5,25 | 2,25 | **6,375** |  |
| 4 | novembre 2011 | **RAE** | 6,50 | 2,00 | **7,500** |  | 5,25 | 2,25 | **6,375** |  |
| 4 | maggio 2012 | **RAE** | 6,50 | 2,00 | **7,500** |  | 5,25 | 2,25 | **6,375** |  |
| 5 | luglio 1997 | **PAE** | 4,50 |  | **4,500** |  | 4,50 |  | **4,500** |  |
| 5 | luglio 1998 | **PAE** | 5,00 | 1,25 | **5,625** |  | 5,50 | 1,50 | **6,250** |  |
| 5 | aprile 1999 | **PAE** | 4,50 | 1,25 | **5,125** |  | 5,00 | 1,50 | **5,750** |  |
| 5 | maggio 2000 | **PAE** | 4,50 | 1,25 | **5,125** |  | 5,00 | 1,50 | **5,750** |  |
| 5 | marzo 2001 | **PAE** | 4,00 | 1,25 | **4,625** |  | 4,00 | 1,50 | **4,750** |  |
| 5 | febbraio 2002 | **PAE** | 4,50 | 1,25 | **5,125** |  | 4,50 | 1,50 | **5,250** |  |
| 5 | gennaio 2003 | **PAE** | 4,50 | 1,25 | **5,125** |  | 4,50 | 1,50 | **5,250** |  |
| 5 | aprile 2004 | **PAE** | 4,50 | 1,25 | **5,125** |  | 4,50 | 1,50 | **5,250** |  |
| 5 | febbraio 2005 | **PAE** | 4,50 | 1,25 | **5,125** |  | 5,00 | 1,50 | **5,750** |  |
| 5 | gennaio 2006 | **PAE** | 4,50 | 1,25 | **5,125** |  | 5,00 | 1,50 | **5,750** |  |
| 5 | marzo 2007 | **PAE** | 4,50 | 1,25 | **5,125** |  | 5,00 | 1,50 | **5,750** |  |
| 5 | marzo 2008 | **PAE** | 4,50 | 1,25 | **5,125** |  | 5,00 | 1,50 | **5,750** |  |
| 5 | aprile 2009 | **PAE** | 4,50 | 1,25 | **5,125** |  | 5,00 | 1,50 | **5,750** |  |
| 5 | maggio 2010 | **PAE** | 4,50 | 1,25 | **5,125** |  | 5,00 | 1,50 | **5,750** |  |
| 5 | settembre 2011 | **PAE** | 4,50 | 1,25 | **5,125** |  | 5,00 | 1,50 | **5,750** |  |
| 5 | novembre 2011 | **PAE** | 4,50 | 1,25 | **5,125** |  | 5,00 | 1,50 | **5,750** |  |
| 5 | luglio 2013 | **PAE** | 4,50 | 1,25 | **5,125** |  | 5,00 | 1,50 | **5,750** |  |
| 6 | settembre 1994 | **PAE** | 3,00 | 1,50 | **3,750** |  | 5,00 | 1,75 | **5,875** | * |
| 6 | ottobre 1995 | **PAE** | 3,00 | 1,50 | **3,750** |  | 5,00 | 1,75 | **5,875** | * |
| 6 | aprile 1997 | **PAE** | 3,00 | 1,50 | **3,750** |  | 5,00 | 1,75 | **5,875** | * |
| 6 | settembre 1997 | **PAE** | 2,75 | 1,50 | **3,500** |  | 4,50 | 1,75 | **5,375** | * |
| 6 | gennaio 1999 | **PAE** | 2,75 | 1,50 | **3,500** |  | 4,50 | 1,75 | **5,375** | * |
| 6 | febbraio 2000 | **PAE** | 2,75 | 1,50 | **3,500** |  | 6,00 | 1,75 | **6,875** | * |
| 6 | marzo 2001 | **PAE** | 2,75 | 1,50 | **3,500** |  | 6,00 | 1,75 | **6,875** | * |
| 6 | giugno 2002 | **PAE** | 3,50 | 1,50 | **4,250** |  | 6,00 | 1,75 | **6,875** | * |
| 6 | febbraio 2003 | **PAE** | 3,50 | 1,50 | **4,250** |  | 6,00 | 1,75 | **6,875** | * |
| 6 | aprile 2004 | **PAE** | 3,50 | 1,75 | **4,375** |  | 6,00 | 2,00 | **7,000** | * |
| 6 | gennaio 2005 | **PAE** | 3,50 | 1,75 | **4,375** |  | 6,00 | 2,00 | **7,000** | * |
| 6 | ottobre 2005 | **PAE** | 3,50 | 1,75 | **4,375** |  | 6,00 | 2,00 | **7,000** | * |
| 6 | settembre 2006 | **PAE** | 3,00 | 1,75 | **3,875** |  | 5,50 | 2,00 | **6,500** | * |
| 6 | febbraio 2008 | **PAE** | 2,50 | 2,00 | **3,500** |  | 5,00 | 2,25 | **6,125** | * |
| 6 | febbraio 2009 | **PAE** | 2,50 | 2,00 | **3,500** |  | 5,00 | 2,25 | **6,125** | * |
| 6 | gennaio 2010 | **PAE** | 2,00 | 2,00 | **3,000** |  | 4,50 | 2,25 | **5,625** | * |
| 6 | febbraio 2011 | **PAE** | 2,00 | 2,00 | **3,000** |  | 4,25 | 2,25 | **5,375** | * |
| 7 | febbraio 1998 | **PAE** | 7,00 | 4,00 | **9,000** | * | 5,50 | 1,00 | **6,000** |  |
| 7 | gennaio 1999 | **PAE** | 7,00 | 4,00 | **9,000** | * | 5,50 | 1,00 | **6,000** |  |
| 7 | settembre 1999 | **PAE** | 7,00 | 4,00 | **9,000** | * | 5,50 | 1,00 | **6,000** |  |
| 7 | dicembre 2000 | **PAE** | 7,00 | 4,00 | **9,000** | * | 5,50 | 1,00 | **6,000** |  |
| 7 | dicembre 2001 | **PAE** | 6,00 | 4,00 | **8,000** | * | 4,75 | 1,00 | **5,250** |  |
| 7 | giugno 2002 | **PAE** | 5,00 | 4,00 | **7,000** | * | 4,00 | 1,25 | **4,625** |  |
| 7 | aprile 2004 | **PAE** | 4,50 | 4,00 | **6,500** | * | 2,50 | 1,25 | **3,125** |  |
| 7 | febbraio 2005 | **PAE** | 4,50 | 4,00 | **6,500** | * | 2,50 | 1,25 | **3,125** |  |
| 7 | marzo 2006 | **PAE** | 4,00 | 4,25 | **6,125** | * | 0,50 | 1,25 | **1,125** |  |
| 7 | novembre 2006 | **PAE** | 4,00 | 4,25 | **6,125** | * | 0,50 | 1,25 | **1,125** |  |
| 7 | settembre 2007 | **PAE** | 4,00 | 4,25 | **6,125** | * | 0,50 | 1,25 | **1,125** |  |
| 7 | luglio 2008 | **PAE** | 4,00 | 4,25 | **6,125** | * | 0,50 | 1,25 | **1,125** |  |
| 7 | settembre 2009 | **PAE** | 4,00 | 4,25 | **6,125** | * | 0,50 | 1,25 | **1,125** |  |
| 7 | novembre 2010 | **PAE** | 4,00 | 4,25 | **6,125** | * | 0,50 | 1,25 | **1,125** |  |
| 7 | novembre 2011 | **PAE** | 4,00 | 4,00 | **6,000** | * | 0,50 | 1,25 | **1,125** |  |
| 7 | novembre 2012 | **PAE** | 4,00 | 4,00 | **6,000** | * | 0,50 | 1,25 | **1,125** |  |
| 7 | settembre 2013 | **PAE** | 4,00 | 4,00 | **6,000** | * | 0,50 | 1,25 | **1,125** |  |
| 8 | dicembre 1994 | **RAE** | 6,00 | 0,75 | **6,375** |  | 6,50 | 0,75 | **6,875** |  |
| 8 | ottobre 1995 | **RAE** | 6,00 | 0,75 | **6,375** |  | 6,50 | 0,75 | **6,875** |  |
| 8 | novembre 1996 | **RAE** | 6,50 | 0,75 | **6,875** |  | 7,00 | 0,75 | **7,375** |  |
| 8 | settembre 1997 | **RAE** | 7,00 | 0,75 | **7,375** |  | 7,25 | 0,75 | **7,625** |  |
| 8 | dicembre 1998 | **RAE** | 7,00 | 0,75 | **7,375** |  | 7,25 | 0,75 | **7,625** |  |
| 8 | gennaio 2000 | **RAE** | 7,00 | 0,75 | **7,375** |  | 7,25 | 0,75 | **7,625** |  |
| 8 | dicembre 2000 | **RAE** | 8,25 | 0,50 | **8,500** |  | 8,50 | 0,50 | **8,750** |  |
| 8 | dicembre 2001 | **RAE** | 7,75 | 0,50 | **8,000** |  | 8,00 | 0,50 | **8,250** |  |
| 8 | dicembre 2002 | **RAE** | 7,75 | 0,50 | **8,000** |  | 8,00 | 0,50 | **8,250** |  |
| 8 | novembre 2003 | **RAE** | 7,75 | 0,50 | **8,000** |  | 8,00 | 0,50 | **8,250** |  |
| 8 | ottobre 2004 | **RAE** | 7,75 | 0,50 | **8,000** |  | 8,00 | 0,50 | **8,250** |  |
| 8 | settembre 2005 | **RAE** | 7,00 | 0,50 | **7,250** |  | 7,50 | 0,50 | **7,750** |  |
| 8 | settembre 2006 | **RAE** | 7,00 | 0,50 | **7,250** |  | 7,50 | 0,50 | **7,750** |  |
| 8 | ottobre 2007 | **RAE** | 7,00 | 0,50 | **7,250** |  | 7,50 | 0,50 | **7,750** |  |
| 8 | settembre 2008 | **RAE** | 7,00 | 0,50 | **7,250** |  | 7,00 | 0,50 | **7,250** |  |
| 8 | febbraio 2010 | **RAE** | 7,00 | 0,50 | **7,250** |  | 7,00 | 0,50 | **7,250** |  |
| 8 | marzo 2011 | **RAE** | 7,00 | 0,50 | **7,250** |  | 6,50 | 0,50 | **6,750** |  |
| 9 | marzo 1997 | **NRAE** | 3,00 | 1,50 | **3,750** | * | 3,00 | 1,50 | **3,750** |  |
| 9 | aprile 1998 | **NRAE** | 3,75 | 1,50 | **4,500** | * | 3,25 | 1,50 | **4,000** |  |
| 9 | maggio 1999 | **NRAE** | 4,00 | 1,50 | **4,750** | * | 3,25 | 1,50 | **4,000** |  |
| 9 | giugno 2000 | **NRAE** | 4,75 | 1,50 | **5,500** | * | 4,00 | 1,50 | **4,750** |  |
| 9 | maggio 2001 | **NRAE** | 4,75 | 1,50 | **5,500** | * | 4,00 | 1,50 | **4,750** |  |
| 9 | maggio 2002 | **NRAE** | 4,25 | 1,50 | **5,000** | * | 4,00 | 1,50 | **4,750** |  |
| 9 | giugno 2003 | **NRAE** | 4,25 | 1,50 | **5,000** | * | 4,00 | 1,50 | **4,750** |  |
| 9 | giugno 2004 | **NRAE** | 4,25 | 1,50 | **5,000** | * | 4,00 | 1,50 | **4,750** |  |
| 9 | maggio 2005 | **NRAE** | 4,25 | 1,50 | **5,000** | * | 4,00 | 1,50 | **4,750** |  |
| 9 | ottobre 2005 | **NRAE** | 4,25 | 1,50 | **5,000** | * | 4,00 | 1,50 | **4,750** |  |
| 9 | febbraio 2007 | **NRAE** | 4,00 | 1,50 | **4,750** | * | 4,00 | 1,50 | **4,750** |  |
| 9 | febbraio 2008 | **NRAE** | 4,00 | 1,50 | **4,750** | * | 4,00 | 1,50 | **4,750** |  |
| 9 | gennaio 2009 | **NRAE** | 3,75 | 1,50 | **4,500** | * | 3,75 | 1,50 | **4,500** |  |
| 9 | ottobre 2009 | **NRAE** | 3,75 | 1,50 | **4,500** | * | 3,75 | 1,50 | **4,500** |  |
| 9 | dicembre 2010 | **NRAE** | 3,75 | 1,50 | **4,500** | * | 3,50 | 1,50 | **4,250** |  |
| 9 | ottobre 2011 | **NRAE** | 3,25 | 1,50 | **4,000** | * | 3,00 | 1,50 | **3,750** |  |
| 9 | novembre 2012 | **NRAE** | 3,25 | 1,50 | **4,000** | * | 3,00 | 1,50 | **3,750** |  |
| 10 | settembre 1996 | **PAE** | 3,75 | 0,75 | **4,125** | * | 3,75 | 1,50 | **4,500** |  |
| 10 | novembre 1997 | **PAE** | 3,00 | 0,75 | **3,375** | * | 3,25 | 1,50 | **4,000** |  |
| 10 | novembre 1998 | **PAE** | 3,00 | 0,75 | **3,375** | * | 3,25 | 1,50 | **4,000** |  |
| 10 | ottobre 1999 | **PAE** | 3,00 | 0,75 | **3,375** | * | 2,50 | 1,50 | **3,250** |  |
| 10 | gennaio 2001 | **PAE** | 2,00 | 0,75 | **2,375** | * | 1,50 | 1,50 | **2,250** |  |
| 10 | febbraio 2002 | **PAE** | 1,50 | 0,75 | **1,875** | * | 1,50 | 1,50 | **2,250** |  |
| 10 | gennaio 2003 | **PAE** | 1,00 | 0,75 | **1,375** | * | 1,00 | 1,50 | **1,750** |  |
| 10 | ottobre 2003 | **PAE** | 0,75 | 1,00 | **1,250** | * | 1,00 | 1,50 | **1,750** |  |
| 10 | gennaio 2005 | **PAE** | 0,75 | 1,00 | **1,250** | * | 1,00 | 1,50 | **1,750** |  |
| 10 | maggio 2006 | **PAE** | 0,75 | 1,00 | **1,250** | * | 1,00 | 1,50 | **1,750** |  |
| 10 | dicembre 2006 | **PAE** | 0,75 | 1,00 | **1,250** | * | 1,00 | 1,50 | **1,750** |  |
| 10 | settembre 2007 | **PAE** | 0,75 | 1,00 | **1,250** | * | 1,00 | 1,50 | **1,750** |  |
| 10 | dicembre 2008 | **PAE** | 0,25 | 1,00 | **0,750** | * | 0,50 | 1,50 | **1,250** |  |
| 10 | ottobre 2009 | **PAE** | 0,25 | 1,00 | **0,750** | * | 0,50 | 1,50 | **1,250** |  |
| 10 | settembre 2010 | **PAE** | 0,00 | 1,00 | **0,500** | * | 0,00 | 1,50 | **0,750** |  |
| 10 | novembre 2011 | **PAE** | 0,00 | 1,00 | **0,500** | * | 0,00 | 1,50 | **0,750** |  |
| 10 | aprile 2013 | **PAE** | 0,00 | 1,00 | **0,500** | * | 0,00 | 1,50 | **0,750** |  |
| 11 | luglio 2000 | **RAE** | 4,50 | 1,00 | **5,000** |  | 4,50 | 1,00 | **5,000** |  |
| 11 | febbraio 2001 | **RAE** | 6,25 | 1,00 | **6,750** |  | 6,25 | 1,25 | **6,875** |  |
| 11 | febbraio 2002 | **RAE** | 6,25 | 1,00 | **6,750** |  | 6,25 | 1,25 | **6,875** |  |
| 11 | dicembre 2002 | **RAE** | 5,75 | 1,00 | **6,250** |  | 6,25 | 1,25 | **6,875** |  |
| 11 | dicembre 2003 | **RAE** | 6,00 | 1,00 | **6,500** |  | 6,00 | 1,00 | **6,500** |  |
| 11 | luglio 2005 | **RAE** | 6,00 | 1,00 | **6,500** |  | 6,00 | 1,00 | **6,500** |  |
| 11 | settembre 2006 | **RAE** | 6,00 | 1,00 | **6,500** |  | 6,00 | 1,00 | **6,500** |  |
| 11 | luglio 2007 | **RAE** | 6,00 | 1,00 | **6,500** |  | 6,00 | 1,00 | **6,500** |  |
| 11 | novembre 2007 | **RAE** | 6,00 | 1,00 | **6,500** |  | 6,00 | 1,00 | **6,500** |  |
| 11 | febbraio 2009 | **RAE** | 6,00 | 1,00 | **6,500** |  | 6,00 | 1,00 | **6,500** |  |
| 11 | giugno 2010 | **RAE** | 6,00 | 1,00 | **6,500** |  | 6,00 | 1,00 | **6,500** |  |
| 11 | settembre 2011 | **RAE** | 6,00 | 1,00 | **6,500** |  | 6,00 | 1,00 | **6,500** |  |
| 11 | aprile 2012 | **RAE** | 6,00 | 1,00 | **6,500** |  | 6,00 | 1,00 | **6,500** |  |
| 11 | luglio 2013 | **RAE** | 6,00 | 1,00 | **6,500** |  | 6,00 | 1,00 | **6,500** |  |
| 11 | luglio 2014 | **RAE** | 6,00 | 1,00 | **6,500** |  | 6,00 | 1,00 | **6,500** |  |
| 11 | marzo 2015 | **RAE** | 6,00 | 1,00 | **6,500** |  | 6,00 | 1,00 | **6,500** |  |
| 11 | maggio 2016 | **RAE** | 6,00 | 1,00 | **6,500** |  | 6,00 | 1,00 | **6,500** |  |
| 12 | settembre 1995 | **PAE** | 1,25 | 1,25 | **1,875** |  | 1,25 | 2,00 | **2,250** | * |
| 12 | novembre 1996 | **PAE** | 2,00 | 1,25 | **2,625** |  | 2,00 | 2,00 | **3,000** | * |
| 12 | ottobre 1997 | **PAE** | 2,75 | 1,25 | **3,375** |  | 2,00 | 2,00 | **3,000** | * |
| 12 | settembre 1998 | **PAE** | 2,75 | 1,25 | **3,375** |  | 1,75 | 2,00 | **2,750** | * |
| 12 | ottobre 1999 | **PAE** | 2,75 | 1,25 | **3,375** |  | 1,75 | 2,00 | **2,750** | * |
| 12 | gennaio 2001 | **PAE** | 3,00 | 1,25 | **3,625** |  | 1,75 | 2,00 | **2,750** | * |
| 12 | maggio 2002 | **PAE** | 3,00 | 1,25 | **3,625** |  | 1,75 | 2,00 | **2,750** | * |
| 12 | gennaio 2003 | **PAE** | 2,50 | 1,25 | **3,125** |  | 1,75 | 2,00 | **2,750** | * |
| 12 | maggio 2004 | **PAE** | 2,00 | 1,25 | **2,625** |  | 1,75 | 2,00 | **2,750** | * |
| 12 | luglio 2005 | **PAE** | 1,25 | 1,25 | **1,875** |  | 1,75 | 2,00 | **2,750** | * |
| 12 | aprile 2006 | **PAE** | 0,75 | 1,25 | **1,375** |  | 1,75 | 2,00 | **2,750** | * |
| 12 | luglio 2007 | **PAE** | 0,25 | 1,25 | **0,875** |  | 1,25 | 2,00 | **2,250** | * |
| 12 | febbraio 2008 | **PAE** | 0,50 | 1,25 | **1,125** |  | 0,50 | 2,00 | **1,500** | * |
| 12 | gennaio 2009 | **PAE** | -0,50 | 1,25 | **0,125** |  | -0,25 | 2,00 | **0,750** | * |
| 12 | luglio 2010 | **PAE** | -0,75 | 1,25 | **-0,125** |  | -0,75 | 2,00 | **0,250** | * |
| 12 | aprile 2011 | **PAE** | -0,75 | 1,25 | **-0,125** |  | -1,00 | 2,00 | **0,000** | * |
| 12 | marzo 2012 | **PAE** | -0,75 | 1,25 | **-0,125** |  | -1,00 | 2,00 | **0,000** | * |
| 13 | febbraio 2000 | **NRAE** | 4,25 | 1,25 | **4,875** |  | 4,25 | 1,00 | **4,750** |  |
| 13 | maggio 2001 | **NRAE** | 5,00 | 1,25 | **5,625** |  | 4,75 | 1,00 | **5,250** |  |
| 13 | febbraio 2002 | **NRAE** | 5,00 | 1,25 | **5,625** |  | 4,75 | 1,00 | **5,250** |  |
| 13 | aprile 2003 | **NRAE** | 5,00 | 1,25 | **5,625** |  | 4,75 | 1,00 | **5,250** |  |
| 13 | giugno 2004 | **NRAE** | 5,00 | 1,25 | **5,625** |  | 4,75 | 1,00 | **5,250** |  |
| 13 | luglio 2005 | **NRAE** | 5,00 | 1,25 | **5,625** |  | 4,75 | 1,00 | **5,250** |  |
| 13 | luglio 2006 | **NRAE** | 5,00 | 1,25 | **5,625** |  | 4,75 | 1,00 | **5,250** |  |
| 13 | marzo 2007 | **NRAE** | 5,00 | 1,25 | **5,625** |  | 4,75 | 1,00 | **5,250** |  |
| 13 | settembre 2008 | **NRAE** | 5,00 | 1,25 | **5,625** |  | 4,75 | 1,00 | **5,250** |  |
| 13 | ottobre 2009 | **NRAE** | 4,75 | 1,00 | **5,250** |  | 4,75 | 1,00 | **5,250** |  |
| 13 | novembre 2010 | **NRAE** | 4,75 | 1,00 | **5,250** |  | 4,50 | 1,00 | **5,000** |  |
| 13 | marzo 2011 | **NRAE** | 4,00 | 1,00 | **4,500** |  | 4,50 | 1,00 | **5,000** |  |
| 13 | giugno 2012 | **NRAE** | 4,00 | 1,00 | **4,500** |  | 4,00 | 1,00 | **4,500** |  |
| 13 | novembre 2013 | **NRAE** | 3,75 | 1,00 | **4,250** |  | 3,75 | 1,00 | **4,250** |  |
| 13 | settembre 2014 | **NRAE** | 3,75 | 1,00 | **4,250** |  | 3,75 | 1,00 | **4,250** |  |
| 13 | luglio 2015 | **NRAE** | 3,50 | 1,00 | **4,000** |  | 3,50 | 1,00 | **4,000** |  |
| 13 | giugno 2016 | **NRAE** | 3,50 | 1,00 | **4,000** |  | 3,50 | 1,00 | **4,000** |  |
| 14 | novembre 2000 | **NRAE** | 4,50 | 1,00 | **5,000** |  | 4,50 | 0,50 | **4,750** |  |
| 14 | luglio 2002 | **NRAE** | 5,00 | 1,00 | **5,500** |  | 5,00 | 0,50 | **5,250** |  |
| 14 | marzo 2003 | **NRAE** | 5,50 | 1,00 | **6,000** |  | 5,50 | 0,50 | **5,750** |  |
| 14 | gennaio 2004 | **NRAE** | 5,50 | 1,00 | **6,000** |  | 5,50 | 0,50 | **5,750** |  |
| 14 | febbraio 2005 | **NRAE** | 5,50 | 1,25 | **6,125** |  | 5,75 | 0,75 | **6,125** |  |
| 14 | gennaio 2006 | **NRAE** | 5,50 | 1,25 | **6,125** |  | 5,75 | 0,75 | **6,125** |  |
| 14 | gennaio 2007 | **NRAE** | 6,00 | 1,25 | **6,625** |  | 6,50 | 0,75 | **6,875** |  |
| 14 | gennaio 2008 | **NRAE** | 6,00 | 1,25 | **6,625** |  | 6,50 | 0,75 | **6,875** |  |
| 14 | dicembre 2008 | **NRAE** | 6,00 | 1,25 | **6,625** |  | 6,50 | 0,75 | **6,875** |  |
| 14 | dicembre 2009 | **NRAE** | 6,00 | 1,25 | **6,625** |  | 6,50 | 0,75 | **6,875** |  |
| 14 | maggio 2011 | **NRAE** | 6,00 | 1,25 | **6,625** |  | 6,50 | 0,75 | **6,875** |  |
| 14 | dicembre 2011 | **NRAE** | 6,00 | 1,25 | **6,625** |  | 6,50 | 0,75 | **6,875** |  |
| 14 | gennaio 2013 | **NRAE** | 6,00 | 1,25 | **6,625** |  | 6,50 | 0,75 | **6,875** |  |
| 14 | dicembre 2013 | **NRAE** | 6,00 | 1,25 | **6,625** |  | 6,50 | 0,75 | **6,875** |  |
| 14 | dicembre 2014 | **NRAE** | 6,00 | 1,25 | **6,625** |  | 6,50 | 0,75 | **6,875** |  |
| 14 | febbraio 2016 | **NRAE** | 6,00 | 1,25 | **6,625** |  | 6,50 | 0,75 | **6,875** |  |
| 14 | maggio 2017 | **NRAE** | 6,00 | 1,25 | **6,625** |  | 6,50 | 0,75 | **6,875** |  |
| 15 | aprile 1997 | **RAE** | 3,50 | 0,50 | **3,750** |  | 3,75 | 0,75 | **4,125** |  |
| 15 | gennaio 1998 | **RAE** | 4,00 | 0,50 | **4,250** |  | 4,00 | 0,75 | **4,375** |  |
| 15 | febbraio 1999 | **RAE** | 4,75 | 0,50 | **5,000** |  | 4,50 | 0,75 | **4,875** |  |
| 15 | maggio 2000 | **RAE** | 4,75 | 0,50 | **5,000** |  | 4,75 | 0,75 | **5,125** |  |
| 15 | luglio 2001 | **RAE** | 4,75 | 0,50 | **5,000** |  | 4,75 | 0,75 | **5,125** |  |
| 15 | luglio 2002 | **RAE** | 4,75 | 0,50 | **5,000** |  | 4,75 | 0,75 | **5,125** |  |
| 15 | aprile 2003 | **RAE** | 4,75 | 0,50 | **5,000** |  | 4,75 | 0,75 | **5,125** |  |
| 15 | dicembre 2003 | **RAE** | 4,75 | 0,50 | **5,000** |  | 4,75 | 0,75 | **5,125** |  |
| 15 | novembre 2004 | **RAE** | 4,75 | 0,50 | **5,000** |  | 4,00 | 0,75 | **4,375** |  |
| 15 | dicembre 2005 | **RAE** | 4,75 | 0,50 | **5,000** |  | 4,00 | 0,75 | **4,375** |  |
| 15 | dicembre 2006 | **RAE** | 4,75 | 0,50 | **5,000** |  | 4,75 | 0,75 | **5,125** |  |
| 15 | gennaio 2008 | **RAE** | 4,75 | 0,50 | **5,000** |  | 4,75 | 0,75 | **5,125** |  |
| 15 | marzo 2009 | **RAE** | 4,75 | 0,50 | **5,000** |  | 4,25 | 0,50 | **4,500** |  |
| 15 | aprile 2010 | **RAE** | 4,75 | 0,50 | **5,000** |  | 4,25 | 0,50 | **4,500** |  |
| 15 | dicembre 2010 | **RAE** | 4,75 | 0,50 | **5,000** |  | 4,25 | 0,50 | **4,500** |  |
| 15 | novembre 2011 | **RAE** | 4,75 | 0,50 | **5,000** |  | 4,25 | 0,50 | **4,500** |  |
| 15 | aprile 2013 | **RAE** | 4,75 | 0,50 | **5,000** |  | 4,25 | 0,50 | **4,500** |  |
| 16 | luglio 1998 | **NRAE** | 2,75 |  | **2,750** |  | 3,25 | 0,50 | **3,500** | * |
| 16 | luglio 1999 | **NRAE** | 3,25 |  | **3,250** |  | 2,75 | 0,50 | **3,000** | * |
| 16 | luglio 2000 | **NRAE** | 4,25 |  | **4,250** |  | 3,75 | 0,50 | **4,000** | * |
| 16 | aprile 2001 | **NRAE** | 3,25 |  | **3,250** |  | 3,00 | 0,50 | **3,250** | * |
| 16 | maggio 2002 | **NRAE** | 4,25 |  | **4,250** |  | 3,00 | 0,50 | **3,250** | * |
| 16 | settembre 2003 | **NRAE** | 4,25 |  | **4,250** |  | 3,00 | 0,50 | **3,250** | * |
| 16 | aprile 2004 | **NRAE** | 4,25 |  | **4,250** |  | 3,00 | 0,50 | **3,250** | * |
| 16 | aprile 2005 | **NRAE** | 4,25 |  | **4,250** |  | 3,00 | 0,50 | **3,250** | * |
| 16 | luglio 2006 | **NRAE** | 3,75 |  | **3,750** |  | 3,00 | 0,50 | **3,250** | * |
| 16 | aprile 2007 | **NRAE** | 2,00 |  | **2,000** |  | 1,75 | 0,50 | **2,000** | * |
| 16 | luglio 2008 | **NRAE** | 2,00 |  | **2,000** |  | 1,75 | 0,50 | **2,000** | * |
| 16 | ottobre 2009 | **NRAE** | 2,00 |  | **2,000** |  | 1,75 | 0,50 | **2,000** | * |
| 16 | giugno 2010 | **NRAE** | 2,00 |  | **2,000** |  | 1,00 | 0,50 | **1,250** | * |
| 16 | dicembre 2011 | **NRAE** | 1,50 |  | **1,500** |  | 1,00 | 0,50 | **1,250** | * |
| 16 | luglio 2012 | **NRAE** | 0,75 |  | **0,750** |  | 0,75 | 0,50 | **1,000** | * |
| 16 | maggio 2013 | **NRAE** | 0,75 |  | **0,750** |  | 0,50 | 0,50 | **0,750** | * |
| 16 | luglio 2014 | **NRAE** | 0,75 |  | **0,750** |  | 0,50 | 0,50 | **0,750** | * |
| 17 | settembre 1998 | **RAE** | 4,00 | 2,25 | **5,125** |  | 4,00 | 2,00 | **5,000** |  |
| 17 | settembre 1999 | **RAE** | 4,00 | 2,25 | **5,125** |  | 4,00 | 2,00 | **5,000** |  |
| 17 | novembre 2000 | **RAE** | 4,00 | 2,25 | **5,125** |  | 4,00 | 2,00 | **5,000** |  |
| 17 | luglio 2001 | **RAE** | 4,75 | 2,25 | **5,875** |  | 4,75 | 2,00 | **5,750** |  |
| 17 | novembre 2002 | **RAE** | 4,75 | 2,25 | **5,875** |  | 4,75 | 2,00 | **5,750** |  |
| 17 | giugno 2003 | **RAE** | 4,50 | 2,25 | **5,625** |  | 4,75 | 2,00 | **5,750** |  |
| 17 | novembre 2004 | **RAE** | 4,50 | 2,25 | **5,625** |  | 4,75 | 2,00 | **5,750** |  |
| 17 | giugno 2005 | **RAE** | 4,50 | 2,25 | **5,625** |  | 4,75 | 2,00 | **5,750** |  |
| 17 | febbraio 2007 | **RAE** | 4,00 | 2,50 | **5,250** |  | 4,75 | 2,00 | **5,750** |  |
| 17 | dicembre 2007 | **RAE** | 4,00 | 2,50 | **5,250** |  | 4,75 | 2,00 | **5,750** |  |
| 17 | settembre 2008 | **RAE** | 4,00 | 2,50 | **5,250** |  | 4,00 | 2,00 | **5,000** |  |
| 17 | marzo 2010 | **RAE** | 4,00 | 2,50 | **5,250** |  | 4,00 | 2,00 | **5,000** |  |
| 17 | gennaio 2011 | **RAE** | 3,50 | 2,50 | **4,750** |  | 4,00 | 2,00 | **5,000** |  |
| 17 | novembre 2011 | **RAE** | 3,00 | 2,25 | **4,125** |  | 3,75 | 2,00 | **4,750** |  |
| 17 | novembre 2012 | **RAE** | 3,00 | 2,25 | **4,125** |  | 3,75 | 2,00 | **4,750** |  |
| 17 | gennaio 2014 | **RAE** | 3,00 | 2,25 | **4,125** |  | 3,75 | 2,00 | **4,750** |  |
| 17 | ottobre 2014 | **RAE** | 3,00 | 2,25 | **4,125** |  | 3,00 | 2,00 | **4,000** |  |
| 18 | febbraio 1998 | **RAE** | 3,00 |  | **3,000** |  | 4,00 | 0,25 | **4,125** |  |
| 18 | febbraio 1999 | **RAE** | 3,00 |  | **3,000** |  | 5,00 | 0,25 | **5,125** |  |
| 18 | novembre 2000 | **RAE** | 5,00 |  | **5,000** |  | 6,50 | 0,25 | **6,625** |  |
| 18 | luglio 2001 | **RAE** | 5,00 |  | **5,000** |  | 6,50 | 0,25 | **6,625** |  |
| 18 | novembre 2002 | **RAE** | 5,00 |  | **5,000** |  | 6,50 | 0,25 | **6,625** |  |
| 18 | ottobre 2003 | **RAE** | 5,00 |  | **5,000** |  | 6,50 | 0,25 | **6,625** |  |
| 18 | novembre 2004 | **RAE** | 5,00 |  | **5,000** |  | 6,50 | 0,25 | **6,625** |  |
| 18 | gennaio 2005 | **RAE** | 5,00 |  | **5,000** |  | 6,25 | 0,25 | **6,375** |  |
| 18 | gennaio 2006 | **RAE** | 5,00 |  | **5,000** |  | 6,25 | 0,25 | **6,375** |  |
| 18 | aprile 2007 | **RAE** | 5,00 |  | **5,000** |  | 6,25 | 0,25 | **6,375** |  |
| 18 | aprile 2008 | **RAE** | 5,00 |  | **5,000** |  | 6,25 | 0,25 | **6,375** |  |
| 18 | aprile 2009 | **RAE** | 5,00 |  | **5,000** |  | 6,00 | 0,25 | **6,125** |  |
| 18 | gennaio 2010 | **RAE** | 5,00 |  | **5,000** |  | 6,00 | 0,25 | **6,125** |  |
| 18 | gennaio 2011 | **RAE** | 5,00 |  | **5,000** |  | 6,00 | 0,25 | **6,125** |  |
| 18 | gennaio 2012 | **RAE** | 5,00 |  | **5,000** |  | 6,00 | 0,25 | **6,125** |  |
| 18 | aprile 2013 | **RAE** | 5,00 |  | **5,000** |  | 6,00 | 0,25 | **6,125** |  |
| 18 | giugno 2014 | **RAE** | 5,00 |  | **5,000** |  | 6,00 | 0,25 | **6,125** |  |
| 19 | dicembre 1996 | **RAE** | 4,00 | 0,50 | **4,250** |  | 4,00 | 0,50 | **4,250** |  |
| 19 | dicembre 1997 | **RAE** | 4,00 | 0,50 | **4,250** |  | 4,00 | 0,50 | **4,250** |  |
| 19 | luglio 1998 | **RAE** | 4,00 | 0,50 | **4,250** |  | 4,00 | 0,50 | **4,250** |  |
| 19 | luglio 1999 | **RAE** | 4,00 | 0,50 | **4,250** |  | 4,00 | 0,50 | **4,250** |  |
| 19 | ottobre 2000 | **RAE** | 4,00 | 0,50 | **4,250** |  | 4,00 | 0,50 | **4,250** |  |
| 19 | maggio 2001 | **RAE** | 4,00 | 0,75 | **4,375** |  | 4,00 | 0,75 | **4,375** |  |
| 19 | luglio 2002 | **RAE** | 4,00 | 0,75 | **4,375** |  | 4,00 | 1,00 | **4,500** |  |
| 19 | marzo 2003 | **RAE** | 4,00 | 0,75 | **4,375** |  | 4,00 | 0,75 | **4,375** |  |
| 19 | settembre 2004 | **RAE** | 4,00 | 0,75 | **4,375** |  | 4,00 | 0,75 | **4,375** |  |
| 19 | settembre 2005 | **RAE** | 4,00 | 0,75 | **4,375** |  | 4,00 | 0,75 | **4,375** |  |
| 19 | luglio 2006 | **RAE** | 4,00 | 0,75 | **4,375** |  | 4,00 | 0,75 | **4,375** |  |
| 19 | giugno 2007 | **RAE** | 4,00 | 0,75 | **4,375** |  | 4,00 | 0,75 | **4,375** |  |
| 19 | ottobre 2008 | **RAE** | 4,00 | 0,75 | **4,375** |  | 4,00 | 0,75 | **4,375** |  |
| 19 | giugno 2009 | **RAE** | 4,00 | 0,75 | **4,375** |  | 4,00 | 0,75 | **4,375** |  |
| 19 | settembre 2010 | **RAE** | 4,00 | 0,75 | **4,375** |  | 4,00 | 0,75 | **4,375** |  |
| 19 | novembre 2011 | **RAE** | 4,00 | 0,75 | **4,375** |  | 4,00 | 0,75 | **4,375** |  |
| 19 | febbraio 2013 | **RAE** | 4,00 | 0,75 | **4,375** |  | 4,00 | 0,75 | **4,375** |  |
| 20 | luglio 1999 | **PAE** | 3,50 | 2,75 | **4,875** |  | 3,50 | 1,50 | **4,250** |  |
| 20 | ottobre 2000 | **PAE** | 4,00 | 2,75 | **5,375** |  | 4,25 | 1,50 | **5,000** |  |
| 20 | maggio 2001 | **PAE** | 5,00 | 2,75 | **6,375** |  | 5,50 | 1,50 | **6,250** |  |
| 20 | luglio 2002 | **PAE** | 4,00 | 2,75 | **5,375** |  | 5,00 | 1,50 | **5,750** |  |
| 20 | ottobre 2003 | **PAE** | 4,00 | 2,75 | **5,375** |  | 5,00 | 1,50 | **5,750** |  |
| 20 | settembre 2004 | **PAE** | 4,00 | 2,75 | **5,375** |  | 5,00 | 1,50 | **5,750** |  |
| 20 | settembre 2005 | **PAE** | 4,00 | 2,75 | **5,375** |  | 5,00 | 1,50 | **5,750** |  |
| 20 | novembre 2006 | **PAE** | 4,00 | 2,50 | **5,250** |  | 5,00 | 1,25 | **5,625** |  |
| 20 | giugno 2007 | **PAE** | 3,75 | 2,50 | **5,000** |  | 4,50 | 1,25 | **5,125** |  |
| 20 | settembre 2008 | **PAE** | 3,75 | 2,50 | **5,000** |  | 4,50 | 1,25 | **5,125** |  |
| 20 | giugno 2009 | **PAE** | 3,75 | 2,50 | **5,000** |  | 4,50 | 1,25 | **5,125** |  |
| 20 | luglio 2010 | **PAE** | 3,75 | 2,75 | **5,125** |  | 4,50 | 1,50 | **5,250** |  |
| 20 | maggio 2011 | **PAE** | 3,75 | 2,75 | **5,125** |  | 4,50 | 1,50 | **5,250** |  |
| 20 | giugno 2012 | **PAE** | 3,75 | 2,75 | **5,125** |  | 4,50 | 1,50 | **5,250** |  |
| 20 | luglio 2013 | **PAE** | 3,75 | 2,75 | **5,125** |  | 4,50 | 1,50 | **5,250** |  |
| 20 | settembre 2014 | **PAE** | 3,75 | 2,75 | **5,125** |  | 4,50 | 1,50 | **5,250** |  |
| 20 | luglio 2015 | **PAE** | 3,75 | 2,75 | **5,125** |  | 4,50 | 1,50 | **5,250** |  |
| 21 | aprile 2000 | **NRAE** | 6,00 | 1,25 | **6,625** |  | 6,50 | 0,75 | **6,875** | * |
| 21 | giugno 2001 | **NRAE** | 6,00 | 1,25 | **6,625** |  | 6,50 | 0,75 | **6,875** | * |
| 21 | maggio 2002 | **NRAE** | 6,00 | 1,25 | **6,625** |  | 6,50 | 0,75 | **6,875** | * |
| 21 | giugno 2003 | **NRAE** | 6,00 | 1,25 | **6,625** |  | 6,50 | 0,75 | **6,875** | * |
| 21 | giugno 2004 | **NRAE** | 6,00 | 1,25 | **6,625** |  | 7,50 | 1,00 | **8,000** | * |
| 21 | settembre 2005 | **NRAE** | 6,00 | 1,25 | **6,625** |  | 7,50 | 1,00 | **8,000** | * |
| 21 | maggio 2006 | **NRAE** | 6,00 | 1,25 | **6,625** |  | 7,50 | 1,00 | **8,000** | * |
| 21 | gennaio 2007 | **NRAE** | 6,00 | 1,25 | **6,625** |  | 7,50 | 1,00 | **8,000** | * |
| 21 | marzo 2008 | **NRAE** | 5,75 | 1,00 | **6,250** |  | 6,50 | 1,00 | **7,000** | * |
| 21 | luglio 2009 | **NRAE** | 5,75 | 1,00 | **6,250** |  | 6,50 | 1,00 | **7,000** | * |
| 21 | febbraio 2010 | **NRAE** | 5,75 | 1,00 | **6,250** |  | 6,50 | 1,00 | **7,000** | * |
| 21 | luglio 2011 | **NRAE** | 5,75 | 1,00 | **6,250** |  | 6,00 | 1,00 | **6,500** | * |
| 21 | marzo 2012 | **NRAE** | 5,75 | 1,00 | **6,250** |  | 6,00 | 1,00 | **6,500** | * |
| 21 | luglio 2013 | **NRAE** | 5,50 | 1,00 | **6,000** |  | 6,00 | 1,00 | **6,500** | * |
| 21 | febbraio 2014 | **NRAE** | 5,50 | 1,00 | **6,000** |  | 6,00 | 1,00 | **6,500** | * |
| 21 | aprile 2015 | **NRAE** | 5,50 | 1,00 | **6,000** |  | 6,00 | 1,00 | **6,500** | * |
| 21 | maggio 2016 | **NRAE** | 5,50 | 1,00 | **6,000** |  | 6,00 | 1,00 | **6,500** | * |
| 22 | luglio 1997 | **PAE** | 3,75 | 1,25 | **4,375** |  | 3,25 | 1,75 | **4,125** |  |
| 22 | ottobre 1998 | **PAE** | 3,75 | 1,25 | **4,375** |  | 3,25 | 1,75 | **4,125** |  |
| 22 | giugno 1999 | **PAE** | 4,25 | 1,25 | **4,875** |  | 3,75 | 1,75 | **4,625** |  |
| 22 | gennaio 2001 | **PAE** | 4,25 | 1,25 | **4,875** |  | 3,75 | 1,75 | **4,625** |  |
| 22 | marzo 2002 | **PAE** | 4,25 | 1,25 | **4,875** |  | 3,75 | 1,75 | **4,625** |  |
| 22 | marzo 2003 | **PAE** | 4,75 | 1,50 | **5,500** |  | 4,75 | 2,00 | **5,750** |  |
| 22 | novembre 2003 | **PAE** | 4,50 | 1,50 | **5,250** |  | 4,75 | 2,00 | **5,750** |  |
| 22 | ottobre 2004 | **PAE** | 4,50 | 1,50 | **5,250** |  | 4,75 | 2,00 | **5,750** |  |
| 22 | novembre 2005 | **PAE** | 4,50 | 1,50 | **5,250** |  | 4,75 | 2,00 | **5,750** |  |
| 22 | giugno 2006 | **PAE** | 4,50 | 1,50 | **5,250** |  | 4,75 | 2,00 | **5,750** |  |
| 22 | gennaio 2008 | **PAE** | 4,50 | 1,50 | **5,250** |  | 4,75 | 2,00 | **5,750** |  |
| 22 | marzo 2009 | **PAE** | 4,50 | 1,50 | **5,250** |  | 4,75 | 2,00 | **5,750** |  |
| 22 | luglio 2009 | **PAE** | 4,50 | 1,50 | **5,250** |  | 4,75 | 2,00 | **5,750** |  |
| 22 | agosto 2010 | **PAE** | 4,50 | 1,50 | **5,250** |  | 4,75 | 2,00 | **5,750** |  |
| 22 | settembre 2011 | **PAE** | 4,50 | 1,50 | **5,250** |  | 4,75 | 2,00 | **5,750** |  |
| 22 | novembre 2012 | **PAE** | 4,50 | 1,50 | **5,250** |  | 4,75 | 1,75 | **5,625** |  |
| 22 | luglio 2013 | **PAE** | 4,50 | 1,50 | **5,250** |  | 4,75 | 1,75 | **5,625** |  |
| 23 | luglio 1994 | **RAE** | 3,00 | 1,00 | **3,500** |  | 3,00 | 1,50 | **3,750** |  |
| 23 | novembre 1995 | **RAE** | 6,00 | 1,25 | **6,625** |  | 6,00 | 1,50 | **6,750** |  |
| 23 | settembre 1996 | **RAE** | 6,50 | 1,50 | **7,250** |  | 6,50 | 1,50 | **7,250** |  |
| 23 | settembre 1997 | **RAE** | 7,00 | 1,25 | **7,625** |  | 7,00 | 1,75 | **7,875** |  |
| 23 | luglio 1998 | **RAE** | 7,00 | 1,25 | **7,625** |  | 7,00 | 1,75 | **7,875** |  |
| 23 | giugno 1999 | **RAE** | 7,00 | 1,25 | **7,625** |  | 7,00 | 1,75 | **7,875** |  |
| 23 | giugno 2000 | **RAE** | 7,00 | 1,25 | **7,625** |  | 7,00 | 1,75 | **7,875** |  |
| 23 | settembre 2001 | **RAE** | 7,00 | 1,25 | **7,625** |  | 7,00 | 1,75 | **7,875** |  |
| 23 | febbraio 2003 | **RAE** | 6,00 | 1,25 | **6,625** |  | 7,00 | 1,75 | **7,875** |  |
| 23 | febbraio 2004 | **RAE** | 6,00 | 1,00 | **6,500** |  | 7,00 | 1,75 | **7,875** |  |
| 23 | ottobre 2004 | **RAE** | 6,00 | 1,00 | **6,500** |  | 7,00 | 1,75 | **7,875** |  |
| 23 | luglio 2005 | **RAE** | 6,00 | 1,00 | **6,500** |  | 7,00 | 1,75 | **7,875** |  |
| 23 | giugno 2006 | **RAE** | 6,00 | 1,00 | **6,500** |  | 7,00 | 1,75 | **7,875** |  |
| 23 | giugno 2007 | **RAE** | 6,00 | 1,00 | **6,500** |  | 7,00 | 1,75 | **7,875** |  |
| 23 | ottobre 2008 | **RAE** | 6,00 | 1,00 | **6,500** |  | 7,00 | 1,75 | **7,875** |  |
| 23 | dicembre 2009 | **RAE** | 6,00 | 1,00 | **6,500** |  | 7,00 | 1,50 | **7,750** |  |
| 23 | novembre 2010 | **RAE** | 6,00 | 1,00 | **6,500** |  | 7,00 | 1,50 | **7,750** |  |
| 24 | settembre 1997 | **RAE** | 1,00 | 3,00 | **2,500** |  | 3,00 | 2,00 | **4,000** |  |
| 24 | settembre 1998 | **RAE** | 2,00 | 3,00 | **3,500** |  | 4,00 | 2,00 | **5,000** |  |
| 24 | maggio 1999 | **RAE** | 3,00 | 3,00 | **4,500** |  | 6,00 | 1,75 | **6,875** |  |
| 24 | settembre 2000 | **RAE** | 4,00 | 3,00 | **5,500** |  | 6,00 | 1,75 | **6,875** |  |
| 24 | giugno 2001 | **RAE** | 4,00 | 3,00 | **5,500** |  | 6,00 | 1,75 | **6,875** |  |
| 24 | settembre 2002 | **RAE** | 3,00 | 3,00 | **4,500** |  | 6,00 | 1,75 | **6,875** |  |
| 24 | ottobre 2003 | **RAE** | 3,00 | 3,00 | **4,500** |  | 6,00 | 1,75 | **6,875** |  |
| 24 | novembre 2004 | **RAE** | 2,50 | 3,25 | **4,125** |  | 6,00 | 2,00 | **7,000** |  |
| 24 | novembre 2005 | **RAE** | 2,50 | 3,25 | **4,125** |  | 6,00 | 2,00 | **7,000** |  |
| 24 | settembre 2006 | **RAE** | 2,50 | 3,25 | **4,125** |  | 6,00 | 2,00 | **7,000** |  |
| 24 | marzo 2007 | **RAE** | 2,50 | 3,25 | **4,125** |  | 6,00 | 2,00 | **7,000** |  |
| 24 | settembre 2008 | **RAE** | 2,50 | 3,25 | **4,125** |  | 6,00 | 2,00 | **7,000** |  |
| 24 | maggio 2009 | **RAE** | 2,50 | 3,25 | **4,125** |  | 6,00 | 2,00 | **7,000** |  |
| 24 | ottobre 2010 | **RAE** | 2,50 | 3,25 | **4,125** |  | 6,00 | 2,00 | **7,000** |  |
| 24 | aprile 2011 | **RAE** | 2,50 | 3,25 | **4,125** |  | 5,50 | 2,00 | **6,500** |  |
| 24 | luglio 2012 | **RAE** | 2,50 | 3,25 | **4,125** |  | 5,50 | 2,00 | **6,500** |  |
| 24 | ottobre 2013 | **RAE** | 2,50 | 3,25 | **4,125** |  | 5,50 | 2,00 | **6,500** |  |
| 25 | giugno 1997 | **RAE** | 5,50 | 2,50 | **6,750** |  | 5,50 | 1,25 | **6,125** |  |
| 25 | settembre 1998 | **RAE** | 6,00 | 2,50 | **7,250** |  | 6,25 | 1,25 | **6,875** |  |
| 25 | novembre 1999 | **RAE** | 6,00 | 2,50 | **7,250** |  | 6,25 | 1,25 | **6,875** |  |
| 25 | maggio 2000 | **RAE** | 6,00 | 2,75 | **7,375** |  | 7,00 | 1,25 | **7,625** |  |
| 25 | gennaio 2001 | **RAE** | 6,00 | 2,75 | **7,375** |  | 7,00 | 1,25 | **7,625** |  |
| 25 | settembre 2002 | **RAE** | 6,00 | 2,50 | **7,250** |  | 7,00 | 1,25 | **7,625** |  |
| 25 | ottobre 2003 | **RAE** | 5,50 | 2,50 | **6,750** |  | 7,00 | 1,25 | **7,625** |  |
| 25 | novembre 2004 | **RAE** | 5,50 | 2,50 | **6,750** |  | 7,00 | 1,25 | **7,625** |  |
| 25 | novembre 2005 | **RAE** | 5,50 | 2,50 | **6,750** |  | 7,00 | 1,25 | **7,625** |  |
| 25 | settembre 2006 | **RAE** | 5,50 | 2,50 | **6,750** |  | 7,00 | 1,25 | **7,625** |  |
| 25 | marzo 2007 | **RAE** | 4,50 | 2,50 | **5,750** |  | 6,50 | 1,25 | **7,125** |  |
| 25 | settembre 2008 | **RAE** | 4,50 | 2,50 | **5,750** |  | 6,50 | 1,25 | **7,125** |  |
| 25 | settembre 2009 | **RAE** | 4,50 | 2,50 | **5,750** |  | 6,50 | 1,25 | **7,125** |  |
| 25 | ottobre 2010 | **RAE** | 4,50 | 2,50 | **5,750** |  | 6,50 | 1,25 | **7,125** |  |
| 25 | aprile 2011 | **RAE** | 4,50 | 2,50 | **5,750** |  | 6,50 | 1,25 | **7,125** |  |
| 25 | settembre 2012 | **RAE** | 4,50 | 2,50 | **5,750** |  | 6,50 | 1,25 | **7,125** |  |
| 25 | marzo 2013 | **RAE** | 4,50 | 2,50 | **5,750** |  | 6,50 | 1,25 | **7,125** |  |
| 26 | settembre 1996 | **RAE** | 2,75 | 0,50 | **3,000** |  | 2,75 |  | **2,750** |  |
| 26 | novembre 1997 | **RAE** | 2,75 | 0,50 | **3,000** |  | 2,75 |  | **2,750** |  |
| 26 | novembre 1998 | **RAE** | 2,75 | 0,50 | **3,000** |  | 2,75 |  | **2,750** |  |
| 26 | luglio 2000 | **RAE** | 2,75 | 0,50 | **3,000** |  | 2,75 |  | **2,750** |  |
| 26 | febbraio 2001 | **RAE** | 2,75 | 0,50 | **3,000** |  | 2,75 |  | **2,750** |  |
| 26 | novembre 2001 | **RAE** | 2,75 | 0,50 | **3,000** |  | 2,75 |  | **2,750** |  |
| 26 | febbraio 2003 | **RAE** | 2,75 | 0,50 | **3,000** |  | 2,75 |  | **2,750** |  |
| 26 | marzo 2004 | **RAE** | 2,50 | 0,50 | **2,750** |  | 2,75 |  | **2,750** |  |
| 26 | marzo 2005 | **RAE** | 2,50 | 0,50 | **2,750** |  | 2,75 |  | **2,750** |  |
| 26 | aprile 2006 | **RAE** | 2,50 | 0,50 | **2,750** |  | 2,75 |  | **2,750** |  |
| 26 | giugno 2007 | **RAE** | 2,50 | 0,50 | **2,750** |  | 2,75 |  | **2,750** |  |
| 26 | marzo 2008 | **RAE** | 2,50 | 0,50 | **2,750** |  | 2,75 |  | **2,750** |  |
| 26 | novembre 2008 | **RAE** | 2,50 | 0,50 | **2,750** |  | 2,75 |  | **2,750** |  |
| 26 | dic-09 | **RAE** | 2,50 | 0,50 | **2,750** |  | 2,75 |  | **2,750** |  |
| 26 | settembre 2010 | **RAE** | 2,50 | 0,50 | **2,750** |  | 3,75 |  | **3,750** |  |
| 26 | settembre 2011 | **RAE** | 2,50 | 0,50 | **2,750** |  | 4,75 |  | **4,750** |  |
| 26 | settembre 2012 | **RAE** | 2,25 | 0,50 | **2,500** |  | 2,50 |  | **2,500** |  |
| 27 | settembre 1999 | **RAE** | 2,50 | 0,50 | **2,750** |  | 2,50 | 0,50 | **2,750** |  |
| 27 | marzo 2001 | **RAE** | 3,00 | 0,50 | **3,250** |  | 3,00 | 0,50 | **3,250** |  |
| 27 | gennaio 2002 | **RAE** | 3,00 | 0,50 | **3,250** |  | 3,00 | 0,50 | **3,250** |  |
| 27 | mag-03 | **RAE** | 3,00 | 0,50 | **3,250** |  | 3,00 | 0,50 | **3,250** |  |
| 27 | marzo 2004 | **RAE** | 3,50 | 0,50 | **3,750** |  | 3,75 | 0,50 | **4,000** |  |
| 27 | maggio 2005 | **RAE** | 4,50 | 0,50 | **4,750** |  | 4,75 | 0,50 | **5,000** |  |
| 27 | gennaio 2006 | **RAE** | 4,50 | 0,50 | **4,750** |  | 4,75 | 0,50 | **5,000** |  |
| 27 | ottobre 2006 | **RAE** | 4,50 | 0,50 | **4,750** |  | 4,75 | 0,75 | **5,125** |  |
| 27 | settembre 2007 | **RAE** | 4,50 | 0,50 | **4,750** |  | 4,75 | 0,75 | **5,125** |  |
| 27 | luglio 2008 | **RAE** | 4,50 | 0,50 | **4,750** |  | 4,75 | 0,75 | **5,125** |  |
| 27 | novembre 2009 | **RAE** | 4,50 | 0,50 | **4,750** |  | 4,75 | 0,75 | **5,125** |  |
| 27 | settembre 2010 | **RAE** | 4,50 | 0,50 | **4,750** |  | 4,75 | 0,75 | **5,125** |  |
| 27 | settembre 2011 | **RAE** | 4,50 | 0,50 | **4,750** |  | 4,75 | 0,75 | **5,125** |  |
| 27 | luglio 2012 | **RAE** | 4,50 | 0,50 | **4,750** |  | 4,75 | 0,75 | **5,125** |  |
| 27 | ottobre 2013 | **RAE** | 4,50 | 0,50 | **4,750** |  | 4,75 | 0,75 | **5,125** |  |
| 27 | luglio 2014 | **RAE** | 4,00 | 0,50 | **4,250** |  | 4,25 | 0,75 | **4,625** |  |
| 27 | novembre 2015 | **RAE** | 4,00 | 0,50 | **4,250** |  | 4,25 | 0,75 | **4,625** |  |
| 28 | marzo 2000 | **NRAE** | 2,50 |  | **2,500** | * | 2,50 |  | **2,500** |  |
| 28 | febbraio 2001 | **NRAE** | 2,50 |  | **2,500** | * | 2,50 |  | **2,500** |  |
| 28 | ottobre 2001 | **NRAE** | 3,50 |  | **3,500** | * | 3,00 |  | **3,000** |  |
| 28 | ottobre 2002 | **NRAE** | 4,00 |  | **4,000** | * | 3,50 |  | **3,500** |  |
| 28 | novembre 2003 | **NRAE** | 4,00 |  | **4,000** | * | 3,50 |  | **3,500** |  |
| 28 | settembre 2004 | **NRAE** | 3,50 |  | **3,500** | * | 3,50 |  | **3,500** |  |
| 28 | novembre 2005 | **NRAE** | 3,50 |  | **3,500** | * | 3,50 |  | **3,500** |  |
| 28 | giugno 2007 | **NRAE** | 3,00 |  | **3,000** | * | 3,50 |  | **3,500** |  |
| 28 | maggio 2008 | **NRAE** | 1,75 |  | **1,750** | * | 2,25 |  | **2,250** |  |
| 28 | febbraio 2009 | **NRAE** | 1,75 |  | **1,750** | * | 2,00 |  | **2,000** |  |
| 28 | maggio 2010 | **NRAE** | 1,25 |  | **1,250** | * | 1,50 |  | **1,500** |  |
| 28 | gennaio 2011 | **NRAE** | 0,50 |  | **0,500** | * | 0,75 |  | **0,750** |  |
| 28 | settembre 2011 | **NRAE** | 0,00 |  | **0,000** | * | 0,00 |  | **0,000** |  |
| 28 | novembre 2012 | **NRAE** | 0,00 |  | **0,000** | * | 0,00 |  | **0,000** |  |
| 28 | ottobre 2013 | **NRAE** | -0,25 |  | **-0,250** | * | -0,50 |  | **-0,500** |  |
| 28 | luglio 2014 | **NRAE** | -0,75 |  | **-0,750** | * | -0,75 |  | **-0,750** |  |
| 28 | dicembre 2015 | **NRAE** | -0,75 |  | **-0,750** | * | -1,00 |  | **-1,000** |  |
| 29 | febbraio 1994 | **RAE** | 3,00 | 0,50 | **3,250** |  | 3,00 | 0,75 | **3,375** |  |
| 29 | settembre 1995 | **RAE** | 3,50 | 0,75 | **3,875** |  | 3,50 | 0,75 | **3,875** |  |
| 29 | ottobre 1996 | **RAE** | 4,00 | 0,75 | **4,375** |  | 4,00 | 0,75 | **4,375** |  |
| 29 | settembre 1997 | **RAE** | 4,00 | 0,75 | **4,375** |  | 4,00 | 0,75 | **4,375** |  |
| 29 | luglio 1998 | **RAE** | 4,00 | 0,75 | **4,375** |  | 4,00 | 0,75 | **4,375** |  |
| 29 | settembre 1999 | **RAE** | 4,00 | 0,75 | **4,375** |  | 4,00 | 0,75 | **4,375** |  |
| 29 | ottobre 2000 | **RAE** | 4,00 | 1,00 | **4,500** |  | 4,00 | 0,75 | **4,375** |  |
| 29 | settembre 2001 | **RAE** | 4,00 | 1,00 | **4,500** |  | 4,00 | 0,75 | **4,375** |  |
| 29 | ottobre 2002 | **RAE** | 4,00 | 1,00 | **4,500** |  | 4,00 | 0,75 | **4,375** |  |
| 29 | settembre 2003 | **RAE** | 4,00 | 1,00 | **4,500** |  | 4,00 | 0,75 | **4,375** |  |
| 29 | marzo 2004 | **RAE** | 4,00 | 1,00 | **4,500** |  | 4,00 | 0,50 | **4,250** |  |
| 29 | gennaio 2005 | **RAE** | 4,00 | 1,00 | **4,500** |  | 4,00 | 0,50 | **4,250** |  |
| 29 | marzo 2006 | **RAE** | 4,00 | 1,00 | **4,500** |  | 4,00 | 0,50 | **4,250** |  |
| 29 | febbraio 2007 | **RAE** | 3,50 | 0,75 | **3,875** |  | 3,50 | 0,50 | **3,750** |  |
| 29 | aprile 2008 | **RAE** | 3,50 | 0,75 | **3,875** |  | 3,50 | 0,50 | **3,750** |  |
| 29 | luglio 2009 | **RAE** | 3,50 | 0,75 | **3,875** |  | 3,50 | 0,50 | **3,750** |  |
| 29 | aprile 2010 | **RAE** | 3,50 | 0,75 | **3,875** |  | 3,50 | 0,50 | **3,750** |  |
| 30 | settembre 1999 | **PAE** | 1,75 | 1,50 | **2,500** | * | 2,00 | 1,00 | **2,500** |  |
| 30 | settembre 2000 | **PAE** | 2,50 | 1,50 | **3,250** | * | 2,50 | 1,00 | **3,000** |  |
| 30 | dicembre 2001 | **PAE** | 2,50 | 1,50 | **3,250** | * | 2,50 | 1,00 | **3,000** |  |
| 30 | ottobre 2002 | **PAE** | 3,00 | 1,25 | **3,625** | * | 3,25 | 1,00 | **3,750** |  |
| 30 | luglio 2003 | **PAE** | 3,00 | 1,25 | **3,625** | * | 3,25 | 1,00 | **3,750** |  |
| 30 | ottobre 2004 | **PAE** | 3,00 | 1,25 | **3,625** | * | 3,25 | 1,00 | **3,750** |  |
| 30 | luglio 2005 | **PAE** | 3,00 | 1,25 | **3,625** | * | 3,25 | 1,00 | **3,750** |  |
| 30 | ottobre 2006 | **PAE** | 3,00 | 1,50 | **3,750** | * | 3,25 | 1,00 | **3,750** |  |
| 30 | settembre 2007 | **PAE** | 3,00 | 1,50 | **3,750** | * | 3,25 | 1,00 | **3,750** |  |
| 30 | febbraio 2009 | **PAE** | 3,00 | 1,50 | **3,750** | * | 3,25 | 1,00 | **3,750** |  |
| 30 | dicembre 2009 | **PAE** | 3,00 | 1,50 | **3,750** | * | 3,50 | 1,00 | **4,000** |  |
| 30 | ottobre 2010 | **PAE** | 3,00 | 1,50 | **3,750** | * | 3,50 | 1,00 | **4,000** |  |
| 30 | dicembre 2011 | **PAE** | 3,00 | 1,50 | **3,750** | * | 3,50 | 1,00 | **4,000** |  |
| 30 | luglio 2012 | **PAE** | 3,00 | 1,50 | **3,750** | * | 3,50 | 1,00 | **4,000** |  |
| 30 | luglio 2013 | **PAE** | 2,50 | 1,50 | **3,250** | * | 3,50 | 1,00 | **4,000** |  |
| 30 | settembre 2014 | **PAE** | 2,50 | 1,50 | **3,250** | * | 3,50 | 1,00 | **4,000** |  |
| 30 | novembre 2015 | **PAE** | 2,50 | 1,50 | **3,250** | * | 3,50 | 1,00 | **4,000** |  |
| 31 | maggio 1997 | **NRAE** | 2,50 | 0,75 | **2,875** |  | 2,50 | 1,75 | **3,375** |  |
| 31 | giugno 1998 | **NRAE** | 3,50 | 0,75 | **3,875** |  | 3,50 | 1,75 | **4,375** |  |
| 31 | luglio 1999 | **NRAE** | 4,00 | 0,75 | **4,375** |  | 4,00 | 2,00 | **5,000** |  |
| 31 | gennaio 2000 | **NRAE** | 4,25 | 0,50 | **4,500** |  | 5,00 | 1,00 | **5,500** |  |
| 31 | novembre 2000 | **NRAE** | 4,25 | 0,50 | **4,500** |  | 5,00 | 1,00 | **5,500** |  |
| 31 | ottobre 2001 | **NRAE** | 5,00 | 0,75 | **5,375** |  | 5,00 | 1,75 | **5,875** |  |
| 31 | marzo 2003 | **NRAE** | 5,00 | 0,75 | **5,375** |  | 5,00 | 1,75 | **5,875** |  |
| 31 | gennaio 2004 | **NRAE** | 4,00 | 0,75 | **4,375** |  | 4,25 | 1,75 | **5,125** |  |
| 31 | maggio 2005 | **NRAE** | 4,00 | 0,75 | **4,375** |  | 4,25 | 1,75 | **5,125** |  |
| 31 | giugno 2006 | **NRAE** | 4,00 | 0,75 | **4,375** |  | 4,25 | 1,75 | **5,125** |  |
| 31 | luglio 2007 | **NRAE** | 4,00 | 0,75 | **4,375** |  | 4,00 | 2,00 | **5,000** |  |
| 31 | luglio 2008 | **NRAE** | 4,00 | 0,75 | **4,375** |  | 4,00 | 2,00 | **5,000** |  |
| 31 | gennaio 2009 | **NRAE** | 4,00 | 0,75 | **4,375** |  | 4,00 | 1,75 | **4,875** |  |
| 31 | dicembre 2009 | **NRAE** | 4,00 | 0,75 | **4,375** |  | 4,00 | 2,00 | **5,000** |  |
| 31 | novembre 2010 | **NRAE** | 3,50 | 0,75 | **3,875** |  | 3,50 | 1,75 | **4,375** |  |
| 31 | dicembre 2011 | **NRAE** | 3,50 | 0,75 | **3,875** |  | 3,50 | 1,75 | **4,375** |  |
| 31 | marzo 2013 | **NRAE** | 2,00 | 0,75 | **2,375** |  | 3,50 | 2,00 | **4,500** |  |
| 32 | ottobre 1994 | **RAE** | 8,00 | 1,00 | **8,500** |  | 8,00 | 1,75 | **8,875** |  |
| 32 | settembre 1995 | **RAE** | 8,50 | 1,00 | **9,000** |  | 9,00 | 1,75 | **9,875** |  |
| 32 | novembre 1996 | **RAE** | 8,50 | 1,00 | **9,000** |  | 9,00 | 1,75 | **9,875** |  |
| 32 | giugno 1998 | **RAE** | 9,00 | 1,25 | **9,625** |  | 9,00 | 1,50 | **9,750** |  |
| 32 | marzo 1999 | **RAE** | 9,50 | 1,00 | **10,000** |  | 9,50 | 1,75 | **10,375** |  |
| 32 | dicembre 1999 | **RAE** | 9,50 | 1,00 | **10,000** |  | 9,50 | 1,75 | **10,375** |  |
| 32 | ottobre 2000 | **RAE** | 9,50 | 1,00 | **10,000** |  | 9,50 | 1,75 | **10,375** |  |
| 32 | settembre 2001 | **RAE** | 9,50 | 1,00 | **10,000** |  | 9,50 | 1,75 | **10,375** |  |
| 32 | novembre 2002 | **RAE** | 9,50 | 1,00 | **10,000** |  | 9,50 | 1,75 | **10,375** |  |
| 32 | gennaio 2004 | **RAE** | 9,50 | 0,75 | **9,875** |  | 9,50 | 1,00 | **10,000** |  |
| 32 | febbraio 2005 | **RAE** | 9,50 | 0,75 | **9,875** |  | 9,50 | 1,00 | **10,000** |  |
| 32 | marzo 2006 | **RAE** | 9,50 | 0,75 | **9,875** |  | 9,50 | 1,00 | **10,000** |  |
| 32 | novembre 2006 | **RAE** | 9,50 | 0,75 | **9,875** |  | 9,50 | 1,00 | **10,000** |  |
| 32 | ottobre 2007 | **RAE** | 9,50 | 0,75 | **9,875** |  | 9,50 | 1,00 | **10,000** |  |
| 32 | luglio 2009 | **RAE** | 9,50 | 0,75 | **9,875** |  | 9,50 | 1,00 | **10,000** |  |
| 32 | marzo 2010 | **RAE** | 9,50 | 0,75 | **9,875** |  | 9,50 | 1,00 | **10,000** |  |
| 32 | gennaio 2011 | **RAE** | 9,50 | 0,75 | **9,875** |  | 9,50 | 1,00 | **10,000** |  |
| 33 | settembre 1993 | **RAE** | 4,50 | 0,50 | **4,750** |  | 5,50 | 0,50 | **5,750** |  |
| 33 | novembre 1994 | **RAE** | 4,50 | 0,50 | **4,750** |  | 5,50 | 0,50 | **5,750** |  |
| 33 | settembre 1995 | **RAE** | 5,00 | 0,50 | **5,250** |  | 6,00 | 0,50 | **6,250** |  |
| 33 | dicembre 1996 | **RAE** | 5,00 | 0,50 | **5,250** |  | 6,00 | 0,50 | **6,250** |  |
| 33 | ottobre 1997 | **RAE** | 5,50 | 0,50 | **5,750** |  | 7,00 | 0,50 | **7,250** |  |
| 33 | gennaio 1999 | **RAE** | 5,50 | 0,50 | **5,750** |  | 7,00 | 0,50 | **7,250** |  |
| 33 | giugno 2000 | **RAE** | 5,00 | 0,50 | **5,250** |  | 6,00 | 0,50 | **6,250** |  |
| 33 | marzo 2001 | **RAE** | 5,50 | 0,50 | **5,750** |  | 7,00 | 0,50 | **7,250** |  |
| 33 | gennaio 2002 | **RAE** | 5,50 | 0,50 | **5,750** |  | 7,00 | 0,50 | **7,250** |  |
| 33 | marzo 2003 | **RAE** | 5,50 | 0,50 | **5,750** |  | 7,00 | 0,50 | **7,250** |  |
| 33 | marzo 2004 | **RAE** | 5,50 | 0,50 | **5,750** |  | 7,00 | 0,50 | **7,250** |  |
| 33 | giugno 2005 | **RAE** | 5,50 | 0,50 | **5,750** |  | 7,00 | 0,50 | **7,250** |  |
| 33 | febbraio 2006 | **RAE** | 5,50 | 0,50 | **5,750** |  | 7,00 | 0,50 | **7,250** |  |
| 33 | maggio 2007 | **RAE** | 5,50 | 0,50 | **5,750** |  | 7,00 | 0,50 | **7,250** |  |
| 33 | febbraio 2008 | **RAE** | 5,50 | 0,50 | **5,750** |  | 7,00 | 0,50 | **7,250** |  |
| 33 | marzo 2009 | **RAE** | 5,50 | 0,50 | **5,750** |  | 7,00 | 0,50 | **7,250** |  |
| 33 | novembre 2009 | **RAE** | 5,50 | 0,50 | **5,750** |  | 7,00 | 0,50 | **7,250** |  |
| 34 | settembre 2000 | **NRAE** | 3,00 | 0,25 | **3,125** |  | 3,00 | 0,50 | **3,250** |  |
| 34 | ottobre 2001 | **NRAE** | 3,00 | 0,25 | **3,125** |  | 3,00 | 0,50 | **3,250** |  |
| 34 | luglio 2002 | **NRAE** | 3,00 | 0,25 | **3,125** |  | 3,00 | 0,50 | **3,250** |  |
| 34 | ottobre 2003 | **NRAE** | 3,00 | 0,25 | **3,125** |  | 3,00 | 0,50 | **3,250** |  |
| 34 | ottobre 2004 | **NRAE** | 3,00 | 0,25 | **3,125** |  | 3,00 | 0,50 | **3,250** |  |
| 34 | febbraio 2006 | **NRAE** | 3,00 | 0,25 | **3,125** |  | 3,00 | 0,50 | **3,250** |  |
| 34 | marzo 2007 | **NRAE** | 3,00 | 0,25 | **3,125** |  | 3,00 | 0,50 | **3,250** |  |
| 34 | novembre 2007 | **NRAE** | 3,00 | 0,25 | **3,125** |  | 3,00 | 0,50 | **3,250** |  |
| 34 | ottobre 2008 | **NRAE** | 3,00 | 0,25 | **3,125** |  | 3,00 | 0,50 | **3,250** |  |
| 34 | settembre 2009 | **NRAE** | 3,00 | 0,25 | **3,125** |  | 3,00 | 0,50 | **3,250** |  |
| 34 | settembre 2010 | **NRAE** | 3,00 | 0,25 | **3,125** |  | 3,00 | 0,50 | **3,250** |  |
| 34 | marzo 2012 | **NRAE** | 3,00 | 0,25 | **3,125** |  | 3,00 | 0,50 | **3,250** |  |
| 34 | novembre 2012 | **NRAE** | 2,50 | 0,25 | **2,625** |  | 2,50 | 0,50 | **2,750** |  |
| 34 | dicembre 2013 | **NRAE** | 2,50 | 0,25 | **2,625** |  | 2,50 | 0,50 | **2,750** |  |
| 34 | ottobre 2014 | **NRAE** | 2,00 | 0,25 | **2,125** |  | 2,50 | 0,50 | **2,750** |  |
| 34 | settembre 2015 | **NRAE** | 2,00 | 0,25 | **2,125** |  | 2,00 | 0,50 | **2,250** |  |
| 34 | settembre 2016 | **NRAE** | 2,00 | 0,25 | **2,125** |  | 2,00 | 0,50 | **2,250** |  |
| 35 | luglio 1999 | **PAE** | 4,50 | 0,75 | **4,875** |  | 4,50 | 0,75 | **4,875** |  |
| 35 | settembre 2000 | **PAE** | 5,50 | 0,75 | **5,875** |  | 5,50 | 0,75 | **5,875** |  |
| 35 | dicembre 2001 | **PAE** | 5,50 | 0,75 | **5,875** |  | 5,50 | 0,75 | **5,875** |  |
| 35 | novembre 2002 | **PAE** | 5,50 | 0,75 | **5,875** |  | 5,50 | 0,75 | **5,875** |  |
| 35 | ottobre 2003 | **PAE** | 5,50 | 0,75 | **5,875** |  | 5,50 | 0,75 | **5,875** |  |
| 35 | settembre 2004 | **PAE** | 5,50 | 0,75 | **5,875** |  | 5,50 | 0,75 | **5,875** |  |
| 35 | settembre 2005 | **PAE** | 5,50 | 0,75 | **5,875** |  | 5,50 | 0,75 | **5,875** |  |
| 35 | ottobre 2006 | **PAE** | 5,50 | 0,75 | **5,875** |  | 5,50 | 0,75 | **5,875** |  |
| 35 | settembre 2007 | **PAE** | 5,50 | 0,75 | **5,875** |  | 5,50 | 0,75 | **5,875** |  |
| 35 | febbraio 2009 | **PAE** | 5,50 | 0,75 | **5,875** |  | 5,50 | 0,75 | **5,875** |  |
| 35 | novembre 2009 | **PAE** | 5,50 | 0,75 | **5,875** |  | 5,50 | 0,75 | **5,875** |  |
| 35 | ottobre 2010 | **PAE** | 5,25 | 1,00 | **5,750** |  | 5,75 | 0,50 | **6,000** |  |
| 35 | settembre 2011 | **PAE** | 5,50 | 1,00 | **6,000** |  | 5,75 | 0,50 | **6,000** |  |
| 35 | dicembre 2012 | **PAE** | 5,00 | 1,00 | **5,500** |  | 5,75 | 0,75 | **6,125** |  |
| 35 | aprile 2014 | **PAE** | 4,50 | 1,00 | **5,000** |  | 5,00 | 0,75 | **5,375** |  |
| 35 | settembre 2014 | **PAE** | 4,50 | 1,00 | **5,000** |  | 5,00 | 0,75 | **5,375** |  |
| 35 | febbraio 2016 | **PAE** | 4,50 | 1,00 | **5,000** |  | 5,00 | 0,75 | **5,375** |  |
| 36 | aprile 1999 | **PAE** | 1,50 | 0,50 | **1,750** |  | 1,50 | 0,50 | **1,750** |  |
| 36 | novembre 2000 | **PAE** | 1,50 | 0,50 | **1,750** |  | 2,50 | 0,50 | **2,750** |  |
| 36 | dicembre 2001 | **PAE** | 2,75 | 0,50 | **3,000** |  | 2,75 | 0,50 | **3,000** |  |
| 36 | settembre 2002 | **PAE** | 2,75 | 0,50 | **3,000** |  | 2,75 | 0,50 | **3,000** |  |
| 36 | giugno 2003 | **PAE** | 2,75 | 0,50 | **3,000** |  | 2,75 | 0,50 | **3,000** |  |
| 36 | maggio 2004 | **PAE** | 2,75 | 0,25 | **2,875** |  | 2,75 | 0,50 | **3,000** |  |
| 36 | aprile 2005 | **PAE** | 2,75 | 0,25 | **2,875** |  | 1,75 | 0,25 | **1,875** |  |
| 36 | luglio 2006 | **PAE** | 3,25 | 0,25 | **3,375** |  | 3,25 | 0,50 | **3,500** |  |
| 36 | marzo 2007 | **PAE** | 3,25 | 0,25 | **3,375** |  | 3,25 | 0,50 | **3,500** |  |
| 36 | aprile 2008 | **PAE** | 3,25 | 0,25 | **3,375** |  | 3,25 | 0,50 | **3,500** |  |
| 36 | giugno 2009 | **PAE** | 3,25 | 0,25 | **3,375** |  | 3,25 | 0,50 | **3,500** |  |
| 36 | luglio 2010 | **PAE** | 3,25 | 0,25 | **3,375** |  | 3,25 | 0,50 | **3,500** |  |
| 36 | maggio 2011 | **PAE** | 3,25 | 0,25 | **3,375** |  | 3,25 | 0,50 | **3,500** |  |
| 36 | luglio 2012 | **PAE** | 2,75 | 0,25 | **2,875** |  | 2,50 | 0,50 | **2,750** |  |
| 36 | giugno 2013 | **PAE** | 2,00 | 0,25 | **2,125** |  | 2,00 | 0,50 | **2,250** |  |
| 36 | aprile 2014 | **PAE** | 2,00 | 0,25 | **2,125** |  | 2,00 | 0,50 | **2,250** |  |
| 36 | luglio 2015 | **PAE** | 2,00 | 0,25 | **2,125** |  | 2,00 | 0,50 | **2,250** |  |
| 37 | maggio 1999 | **PAE** | 5,50 | 0,50 | **5,750** |  | 5,50 |  | **5,500** | * |
| 37 | febbraio 2000 | **PAE** | 5,50 | 0,50 | **5,750** |  | 5,50 |  | **5,500** | * |
| 37 | settembre 2000 | **PAE** | 5,50 | 0,50 | **5,750** |  | 5,50 |  | **5,500** | * |
| 37 | dicembre 2001 | **PAE** | 6,25 | 0,50 | **6,500** |  | 6,25 |  | **6,250** | * |
| 37 | ottobre 2002 | **PAE** | 7,00 | 0,50 | **7,250** |  | 6,25 |  | **6,250** | * |
| 37 | ottobre 2003 | **PAE** | 6,00 | 0,50 | **6,250** |  | 5,75 |  | **5,750** | * |
| 37 | gennaio 2005 | **PAE** | 6,00 | 0,50 | **6,250** |  | 5,75 |  | **5,750** | * |
| 37 | aprile 2006 | **PAE** | 5,50 | 0,50 | **5,750** |  | 5,75 |  | **5,750** | * |
| 37 | maggio 2007 | **PAE** | 5,50 | 0,50 | **5,750** |  | 5,75 |  | **5,750** | * |
| 37 | maggio 2008 | **PAE** | 5,50 | 0,50 | **5,750** |  | 5,75 |  | **5,750** | * |
| 37 | febbraio 2009 | **PAE** | 5,50 | 0,50 | **5,750** |  | 5,50 |  | **5,500** | * |
| 37 | giugno 2010 | **PAE** | 4,50 | 0,50 | **4,750** |  | 4,50 |  | **4,500** | * |
| 37 | marzo 2011 | **PAE** | 3,50 | 0,50 | **3,750** |  | 3,50 |  | **3,500** | * |
| 37 | aprile 2012 | **PAE** | 3,50 | 0,50 | **3,750** |  | 3,50 |  | **3,500** | * |
| 37 | gennaio 2013 | **PAE** | 3,00 | 0,50 | **3,250** |  | 2,00 |  | **2,000** | * |
| 37 | gennaio 2014 | **PAE** | 3,00 | 0,50 | **3,250** |  | 2,00 |  | **2,000** | * |
| 37 | gennaio 2015 | **PAE** | 3,00 | 0,50 | **3,250** |  | 3,00 |  | **3,000** | * |
| 38 | gennaio 1999 | **PAE** | 5,25 | 0,50 | **5,500** | * | 5,00 |  | **5,000** |  |
| 38 | giugno 1999 | **PAE** | 5,25 | 0,50 | **5,500** | * | 5,00 |  | **5,000** |  |
| 38 | luglio 2000 | **PAE** | 5,50 | 0,50 | **5,750** | * | 5,00 |  | **5,000** |  |
| 38 | settembre 2001 | **PAE** | 5,50 | 0,50 | **5,750** | * | 6,00 |  | **6,000** |  |
| 38 | novembre 2002 | **PAE** | 5,50 | 0,50 | **5,750** | * | 6,50 |  | **6,500** |  |
| 38 | luglio 2003 | **PAE** | 5,50 | 0,50 | **5,750** | * | 6,50 |  | **6,500** |  |
| 38 | settembre 2004 | **PAE** | 5,50 | 0,50 | **5,750** | * | 6,50 |  | **6,500** |  |
| 38 | ottobre 2005 | **PAE** | 5,50 | 0,50 | **5,750** | * | 6,50 |  | **6,500** |  |
| 38 | ottobre 2006 | **PAE** | 5,50 | 0,50 | **5,750** | * | 6,50 |  | **6,500** |  |
| 38 | luglio 2007 | **PAE** | 5,50 | 0,50 | **5,750** | * | 6,00 |  | **6,000** |  |
| 38 | luglio 2008 | **PAE** | 5,50 | 0,50 | **5,750** | * | 6,00 |  | **6,000** |  |
| 38 | settembre 2009 | **PAE** | 4,50 | 0,50 | **4,750** | * | 6,00 |  | **6,000** |  |
| 38 | luglio 2010 | **PAE** | 4,50 | 0,50 | **4,750** | * | 6,00 |  | **6,000** |  |
| 38 | ottobre 2011 | **PAE** | 4,50 | 0,50 | **4,750** | * | 5,00 |  | **5,000** |  |
| 38 | dicembre 2012 | **PAE** | 4,50 | 0,50 | **4,750** | * | 5,50 |  | **5,500** |  |
| 38 | luglio 2013 | **PAE** | 4,50 | 0,50 | **4,750** | * | 5,50 |  | **5,500** |  |
| 38 | settembre 2014 | **PAE** | 4,50 | 0,50 | **4,750** | * | 5,50 |  | **5,500** |  |
| 39 | dicembre 1999 | **NRAE** | 2,00 | 0,50 | **2,250** |  | 2,00 | 0,50 | **2,250** |  |
| 39 | settembre 2000 | **NRAE** | 2,00 | 0,50 | **2,250** |  | 2,00 | 0,50 | **2,250** |  |
| 39 | dicembre 2001 | **NRAE** | 2,00 | 0,50 | **2,250** |  | 2,00 | 0,50 | **2,250** |  |
| 39 | settembre 2002 | **NRAE** | 2,50 | 0,50 | **2,750** |  | 2,50 | 0,50 | **2,750** |  |
| 39 | novembre 2003 | **NRAE** | 2,50 | 0,50 | **2,750** |  | 2,50 | 0,50 | **2,750** |  |
| 39 | dicembre 2004 | **NRAE** | 2,50 | 0,50 | **2,750** |  | 2,50 | 0,50 | **2,750** |  |
| 39 | settembre 2005 | **NRAE** | 2,50 | 0,50 | **2,750** |  | 2,50 | 0,50 | **2,750** |  |
| 39 | febbraio 2007 | **NRAE** | 2,50 | 0,50 | **2,750** |  | 2,50 | 0,50 | **2,750** |  |
| 39 | marzo 2008 | **NRAE** | 2,50 | 0,50 | **2,750** |  | 2,50 | 0,50 | **2,750** |  |
| 39 | dicembre 2008 | **NRAE** | 2,50 | 0,50 | **2,750** |  | 2,50 | 0,50 | **2,750** |  |
| 39 | novembre 2009 | **NRAE** | 2,50 | 0,25 | **2,625** |  | 2,50 | 0,25 | **2,625** |  |
| 39 | maggio 2011 | **NRAE** | 2,50 | 0,25 | **2,625** |  | 2,50 | 0,25 | **2,625** |  |
| 39 | maggio 2012 | **NRAE** | 2,50 | 0,25 | **2,625** |  | 2,50 | 0,25 | **2,625** |  |
| 39 | maggio 2013 | **NRAE** | 2,50 | 0,25 | **2,625** |  | 2,50 | 0,25 | **2,625** |  |
| 39 | giugno 2014 | **NRAE** | 2,50 | 0,25 | **2,625** |  | 2,50 | 0,25 | **2,625** |  |
| 39 | gennaio 2015 | **NRAE** | 2,50 | 0,25 | **2,625** |  | 2,50 | 0,25 | **2,625** |  |
| 39 | marzo 2016 | **NRAE** | 2,50 | 0,25 | **2,625** |  | 2,50 | 0,25 | **2,625** |  |
| 40 | aprile 1993 | **NRAE** | 5,00 | 0,50 | **5,250** |  | 4,25 |  | **4,250** |  |
| 40 | marzo 1994 | **NRAE** | 5,00 | 0,50 | **5,250** |  | 4,25 |  | **4,250** |  |
| 40 | gennaio 1995 | **NRAE** | 5,00 | 0,50 | **5,250** |  | 4,25 |  | **4,250** |  |
| 40 | novembre 1996 | **NRAE** | 4,75 | 0,50 | **5,000** |  | 4,75 |  | **4,750** |  |
| 40 | novembre 1997 | **NRAE** | 4,50 | 0,75 | **4,875** |  | 5,00 |  | **5,000** |  |
| 40 | novembre 1998 | **NRAE** | 4,50 | 0,75 | **4,875** |  | 5,00 |  | **5,000** |  |
| 40 | novembre 1999 | **NRAE** | 4,50 | 0,75 | **4,875** |  | 5,00 |  | **5,000** |  |
| 40 | novembre 2000 | **NRAE** | 4,50 | 0,75 | **4,875** |  | 5,00 |  | **5,000** |  |
| 40 | novembre 2001 | **NRAE** | 4,50 | 0,75 | **4,875** |  | 5,00 |  | **5,000** |  |
| 40 | novembre 2002 | **NRAE** | 4,50 | 0,75 | **4,875** |  | 5,00 |  | **5,000** |  |
| 40 | settembre 2003 | **NRAE** | 4,50 | 0,75 | **4,875** |  | 6,00 |  | **6,000** |  |
| 40 | luglio 2004 | **NRAE** | 4,50 | 0,75 | **4,875** |  | 5,00 |  | **5,000** |  |
| 40 | aprile 2005 | **NRAE** | 4,50 | 0,75 | **4,875** |  | 5,00 |  | **5,000** |  |
| 40 | maggio 2006 | **NRAE** | 4,50 | 0,75 | **4,875** |  | 4,00 |  | **4,000** |  |
| 40 | settembre 2007 | **NRAE** | 4,50 | 0,75 | **4,875** |  | 4,00 |  | **4,000** |  |
| 40 | marzo 2008 | **NRAE** | 4,50 | 0,75 | **4,875** |  | 5,00 |  | **5,000** |  |
| 40 | ottobre 2009 | **NRAE** | 4,50 | 0,75 | **4,875** |  | 4,25 |  | **4,250** |  |
| 41 | luglio 1992 | **NRAE** | 1,50 |  | **1,500** |  | 1,50 |  | **1,500** |  |
| 41 | giugno 1993 | **NRAE** | 1,50 |  | **1,500** |  | 1,50 |  | **1,500** |  |
| 41 | settembre 1994 | **NRAE** | 2,50 |  | **2,500** |  | 3,00 |  | **3,000** |  |
| 41 | luglio 1995 | **NRAE** | 3,25 |  | **3,250** |  | 3,00 |  | **3,000** |  |
| 41 | luglio 1996 | **NRAE** | 3,25 |  | **3,250** |  | 3,00 |  | **3,000** |  |
| 41 | luglio 1997 | **NRAE** | 3,00 |  | **3,000** |  | 3,00 |  | **3,000** |  |
| 41 | ottobre 1998 | **NRAE** | 3,00 |  | **3,000** |  | 3,00 |  | **3,000** |  |
| 41 | settembre 1999 | **NRAE** | 3,00 |  | **3,000** |  | 3,00 |  | **3,000** |  |
| 41 | giugno 2000 | **NRAE** | 2,00 |  | **2,000** |  | 2,00 |  | **2,000** |  |
| 41 | settembre 2001 | **NRAE** | 1,50 |  | **1,500** |  | 1,50 |  | **1,500** |  |
| 41 | settembre 2002 | **NRAE** | 1,50 |  | **1,500** |  | 1,50 |  | **1,500** |  |
| 41 | settembre 2003 | **NRAE** | 1,50 |  | **1,500** |  | 1,50 |  | **1,500** |  |
| 41 | luglio 2004 | **NRAE** | 1,00 |  | **1,000** |  | 1,00 |  | **1,000** |  |
| 41 | ottobre 2005 | **NRAE** | 1,00 |  | **1,000** |  | 1,00 |  | **1,000** |  |
| 41 | settembre 2006 | **NRAE** | 0,50 |  | **0,500** |  | 0,50 |  | **0,500** |  |
| 41 | giugno 2007 | **NRAE** | 0,00 |  | **0,000** |  | 0,00 |  | **0,000** |  |
| 41 | giugno 2008 | **NRAE** | 0,00 |  | **0,000** |  | 0,00 |  | **0,000** |  |
| 42 | settembre 1999 | **NRAE** | 6,50 | 0,75 | **6,875** |  | 6,00 | 1,00 | **6,500** |  |
| 42 | ottobre 2000 | **NRAE** | 7,00 | 0,75 | **7,375** |  | 6,50 | 1,00 | **7,000** |  |
| 42 | ottobre 2001 | **NRAE** | 7,25 | 0,75 | **7,625** |  | 7,25 | 1,00 | **7,750** |  |
| 42 | settembre 2002 | **NRAE** | 7,25 | 0,75 | **7,625** |  | 7,25 | 1,00 | **7,750** |  |
| 42 | gennaio 2004 | **NRAE** | 7,25 | 0,75 | **7,625** |  | 7,25 | 1,00 | **7,750** |  |
| 42 | luglio 2005 | **NRAE** | 6,25 | 0,50 | **6,500** |  | 6,25 | 0,75 | **6,625** |  |
| 42 | marzo 2006 | **NRAE** | 6,25 | 0,50 | **6,500** |  | 6,25 | 0,75 | **6,625** |  |
| 42 | aprile 2007 | **NRAE** | 6,50 | 0,50 | **6,750** |  | 6,50 | 0,75 | **6,875** |  |
| 42 | gennaio 2008 | **NRAE** | 6,50 | 0,75 | **6,875** |  | 6,50 | 0,75 | **6,875** |  |
| 42 | novembre 2008 | **NRAE** | 5,75 | 0,75 | **6,125** |  | 5,75 | 1,00 | **6,250** |  |
| 42 | settembre 2009 | **NRAE** | 5,75 | 0,75 | **6,125** |  | 5,75 | 1,00 | **6,250** |  |
| 42 | ottobre 2010 | **NRAE** | 5,75 | 0,75 | **6,125** |  | 5,75 | 1,00 | **6,250** |  |
| 42 | settembre 2011 | **NRAE** | 5,25 | 0,75 | **5,625** |  | 5,75 | 1,00 | **6,250** |  |
| 42 | ottobre 2012 | **NRAE** | 5,25 | 0,50 | **5,500** |  | 5,50 | 0,75 | **5,875** |  |
| 42 | settembre 2013 | **NRAE** | 5,25 | 0,50 | **5,500** |  | 5,50 | 0,75 | **5,875** |  |
| 42 | novembre 2014 | **NRAE** | 5,25 | 0,50 | **5,500** |  | 5,50 | 0,75 | **5,875** |  |
| 42 | dicembre 2015 | **NRAE** | 5,25 | 0,50 | **5,500** |  | 5,50 | 0,75 | **5,875** |  |
| 43 | febbraio 1996 | **NRAE** | 1,50 |  | **1,500** |  | 2,00 |  | **2,000** |  |
| 43 | gennaio 1997 | **NRAE** | 2,00 |  | **2,000** |  | 3,00 |  | **3,000** |  |
| 43 | giugno 1998 | **NRAE** | 4,00 |  | **4,000** |  | 3,00 |  | **3,000** |  |
| 43 | gennaio 1999 | **NRAE** | 5,00 |  | **5,000** |  | 3,00 |  | **3,000** |  |
| 43 | dicembre 1999 | **NRAE** | 5,00 |  | **5,000** |  | 5,00 |  | **5,000** |  |
| 43 | ottobre 2000 | **NRAE** | 5,50 |  | **5,500** |  | 5,00 |  | **5,000** |  |
| 43 | novembre 2001 | **NRAE** | 5,50 |  | **5,500** |  | 5,00 |  | **5,000** |  |
| 43 | febbraio 2003 | **NRAE** | 5,50 |  | **5,500** |  | 5,00 |  | **5,000** |  |
| 43 | dicembre 2003 | **NRAE** | 5,50 |  | **5,500** |  | 5,00 |  | **5,000** |  |
| 43 | marzo 2005 | **NRAE** | 5,50 |  | **5,500** |  | 5,00 |  | **5,000** |  |
| 43 | giugno 2006 | **NRAE** | 5,50 |  | **5,500** |  | 5,00 |  | **5,000** |  |
| 43 | giugno 2007 | **NRAE** | 5,50 |  | **5,500** |  | 5,00 |  | **5,000** |  |
| 43 | novembre 2007 | **NRAE** | 5,00 |  | **5,000** |  | 5,00 |  | **5,000** |  |
| 43 | marzo 2009 | **NRAE** | 5,00 |  | **5,000** |  | 5,00 |  | **5,000** |  |
| 43 | maggio 2010 | **NRAE** | 5,00 |  | **5,000** |  | 5,00 |  | **5,000** |  |
| 43 | luglio 2011 | **NRAE** | 5,00 |  | **5,000** |  | 5,00 |  | **5,000** |  |
| 43 | marzo 2012 | **NRAE** | 5,00 |  | **5,000** |  | 5,00 |  | **5,000** |  |
| 44 | gennaio 2001 | **RAE** | 3,75 |  | **3,750** |  | 5,50 | 0,25 | **5,625** |  |
| 44 | febbraio 2002 | **RAE** | 3,75 |  | **3,750** |  | 6,00 | 0,50 | **6,250** |  |
| 44 | maggio 2003 | **RAE** | 3,75 |  | **3,750** |  | 6,00 | 0,50 | **6,250** |  |
| 44 | gennaio 2004 | **RAE** | 3,75 |  | **3,750** |  | 5,25 | 0,50 | **5,500** |  |
| 44 | aprile 2005 | **RAE** | 4,50 |  | **4,500** |  | 4,75 | 0,50 | **5,000** |  |
| 44 | marzo 2006 | **RAE** | 3,50 |  | **3,500** |  | 5,50 | 0,50 | **5,750** |  |
| 44 | dicembre 2006 | **RAE** | 3,50 |  | **3,500** |  | 5,50 | 0,50 | **5,750** |  |
| 44 | marzo 2008 | **RAE** | 3,50 |  | **3,500** |  | 5,50 | 0,50 | **5,750** |  |
| 44 | dicembre 2008 | **RAE** | 3,50 |  | **3,500** |  | 5,50 | 0,75 | **5,875** |  |
| 44 | aprile 2010 | **RAE** | 3,50 |  | **3,500** |  | 4,50 | 1,00 | **5,000** |  |
| 44 | giugno 2011 | **RAE** | 3,50 |  | **3,500** |  | 4,50 | 1,00 | **5,000** |  |
| 44 | dicembre 2011 | **RAE** | 3,50 |  | **3,500** |  | 4,50 | 1,00 | **5,000** |  |
| 44 | dicembre 2012 | **RAE** | 3,50 |  | **3,500** |  | 4,00 | 1,00 | **4,500** |  |
| 44 | dicembre 2013 | **RAE** | 3,50 |  | **3,500** |  | 4,00 | 1,00 | **4,500** |  |
| 44 | ottobre 2014 | **RAE** | 3,00 |  | **3,000** |  | 3,50 | 1,00 | **4,000** |  |
| 44 | novembre 2015 | **RAE** | 3,00 |  | **3,000** |  | 3,50 | 1,00 | **4,000** |  |
| 44 | novembre 2016 | **RAE** | 3,00 |  | **3,000** |  | 3,50 | 1,00 | **4,000** |  |
| 45 | ottobre 1997 | **NRAE** | 4,00 | 1,00 | **4,500** |  | 4,00 | 1,25 | **4,625** |  |
| 45 | ottobre 1998 | **NRAE** | 4,00 | 1,00 | **4,500** |  | 4,00 | 1,25 | **4,625** |  |
| 45 | novembre 1999 | **NRAE** | 4,00 | 1,50 | **4,750** |  | 5,00 | 1,25 | **5,625** |  |
| 45 | ottobre 2000 | **NRAE** | 4,00 | 1,50 | **4,750** |  | 5,00 | 1,25 | **5,625** |  |
| 45 | dicembre 2001 | **NRAE** | 4,00 | 1,50 | **4,750** |  | 5,00 | 1,25 | **5,625** |  |
| 45 | gennaio 2003 | **NRAE** | 4,50 | 1,50 | **5,250** |  | 5,50 | 1,50 | **6,250** |  |
| 45 | gennaio 2004 | **NRAE** | 4,50 | 1,50 | **5,250** |  | 5,00 | 1,50 | **5,750** |  |
| 45 | novembre 2004 | **NRAE** | 4,00 | 1,50 | **4,750** |  | 4,00 | 1,50 | **4,750** |  |
| 45 | dicembre 2005 | **NRAE** | 4,00 | 1,00 | **4,500** |  | 4,00 | 1,25 | **4,625** |  |
| 45 | aprile 2007 | **NRAE** | 4,00 | 1,50 | **4,750** |  | 4,00 | 1,50 | **4,750** |  |
| 45 | aprile 2008 | **NRAE** | 4,00 | 1,50 | **4,750** |  | 4,00 | 1,50 | **4,750** |  |
| 45 | giugno 2009 | **NRAE** | 4,00 | 1,50 | **4,750** |  | 4,00 | 1,50 | **4,750** |  |
| 45 | novembre 2009 | **NRAE** | 4,00 | 1,50 | **4,750** |  | 4,00 | 1,50 | **4,750** |  |
| 45 | novembre 2010 | **NRAE** | 4,00 | 1,50 | **4,750** |  | 4,00 | 1,50 | **4,750** |  |
| 45 | novembre 2011 | **NRAE** | 4,00 | 1,50 | **4,750** |  | 4,00 | 1,50 | **4,750** |  |
| 45 | novembre 2012 | **NRAE** | 4,00 | 1,50 | **4,750** |  | 4,00 | 1,50 | **4,750** |  |
| 45 | dicembre 2013 | **NRAE** | 4,00 | 1,50 | **4,750** |  | 4,00 | 1,50 | **4,750** |  |
| 46 | ottobre 1998 | **PAE** | 3,50 | 0,75 | **3,875** |  | 3,50 | 0,50 | **3,750** |  |
| 46 | ottobre 1999 | **PAE** | 3,50 | 0,75 | **3,875** |  | 3,50 | 0,50 | **3,750** |  |
| 46 | novembre 2000 | **PAE** | 3,50 | 0,75 | **3,875** |  | 3,50 | 0,50 | **3,750** |  |
| 46 | maggio 2002 | **PAE** | 2,00 | 0,50 | **2,250** |  | 2,00 | 0,75 | **2,375** |  |
| 46 | giugno 2003 | **PAE** | 2,00 | 0,50 | **2,250** |  | 2,00 | 0,75 | **2,375** |  |
| 46 | giugno 2004 | **PAE** | 2,00 | 0,50 | **2,250** |  | 2,00 | 0,75 | **2,375** |  |
| 46 | giugno 2005 | **PAE** | 2,00 | 0,50 | **2,250** |  | 2,00 | 0,75 | **2,375** |  |
| 46 | febbraio 2006 | **PAE** | 2,00 | 0,50 | **2,250** |  | 2,00 | 0,75 | **2,375** |  |
| 46 | marzo 2007 | **PAE** | 2,00 | 0,75 | **2,375** |  | 2,00 | 0,50 | **2,250** |  |
| 46 | maggio 2008 | **PAE** | 1,50 | 0,50 | **1,750** |  | 1,50 | 0,75 | **1,875** |  |
| 46 | giugno 2009 | **PAE** | 0,75 | 0,50 | **1,000** |  | 1,50 | 0,75 | **1,875** |  |
| 46 | aprile 2010 | **PAE** | 0,75 | 0,75 | **1,125** |  | 1,00 | 0,50 | **1,250** |  |
| 46 | luglio 2011 | **PAE** | 0,00 | 0,75 | **0,375** |  | 1,00 | 0,50 | **1,250** |  |
| 46 | aprile 2012 | **PAE** | 0,00 | 0,75 | **0,375** |  | 1,00 | 0,25 | **1,125** |  |
| 46 | febbraio 2013 | **PAE** | 0,00 | 0,75 | **0,375** |  | 0,50 | 0,25 | **0,625** |  |
| 46 | giugno 2014 | **PAE** | 0,00 | 0,75 | **0,375** |  | 0,50 | 0,25 | **0,625** |  |
| 46 | giugno 2015 | **PAE** | 0,00 | 0,75 | **0,375** |  | 0,50 | 0,25 | **0,625** |  |
| 47 | luglio 1996 | **PAE** | 5,00 | 1,50 | **5,750** | * | 5,50 | 1,50 | **6,250** |  |
| 47 | maggio 1997 | **PAE** | 5,00 | 1,50 | **5,750** | * | 6,00 | 1,50 | **6,750** |  |
| 47 | giugno 1998 | **PAE** | 5,25 | 1,50 | **6,000** | * | 6,00 | 1,50 | **6,750** |  |
| 47 | febbraio 1999 | **PAE** | 6,00 | 1,50 | **6,750** | * | 6,00 | 1,50 | **6,750** |  |
| 47 | gennaio 2000 | **PAE** | 6,00 | 1,50 | **6,750** | * | 6,00 | 1,50 | **6,750** |  |
| 47 | luglio 2001 | **PAE** | 5,50 | 1,50 | **6,250** | * | 6,00 | 1,50 | **6,750** |  |
| 47 | maggio 2002 | **PAE** | 5,00 | 1,50 | **5,750** | * | 6,00 | 1,50 | **6,750** |  |
| 47 | gennaio 2003 | **PAE** | 4,50 | 1,50 | **5,250** | * | 6,00 | 1,50 | **6,750** |  |
| 47 | dicembre 2003 | **PAE** | 4,00 | 1,50 | **4,750** | * | 6,00 | 1,50 | **6,750** |  |
| 47 | ottobre 2004 | **PAE** | 4,00 | 1,50 | **4,750** | * | 6,00 | 1,50 | **6,750** |  |
| 47 | gennaio 2006 | **PAE** | 4,00 | 1,50 | **4,750** | * | 6,00 | 1,50 | **6,750** |  |
| 47 | febbraio 2007 | **PAE** | 4,00 | 1,50 | **4,750** | * | 6,00 | 1,50 | **6,750** |  |
| 47 | aprile 2008 | **PAE** | 3,50 | 1,50 | **4,250** | * | 6,00 | 1,50 | **6,750** |  |
| 47 | maggio 2009 | **PAE** | 3,50 | 1,50 | **4,250** | * | 6,00 | 1,50 | **6,750** |  |
| 47 | gennaio 2010 | **PAE** | 3,50 | 1,50 | **4,250** | * | 6,00 | 1,50 | **6,750** |  |
| 47 | gennaio 2011 | **PAE** | 3,50 | 1,50 | **4,250** | * | 6,00 | 1,50 | **6,750** |  |
| 47 | luglio 2012 | **PAE** | 3,50 | 1,50 | **4,250** | * | 6,00 | 1,50 | **6,750** |  |
| 48 | maggio 1997 | **RAE** | 1,00 | 1,00 | **1,500** |  | 0,75 | 1,75 | **1,625** |  |
| 48 | gennaio 1998 | **RAE** | 1,00 | 1,00 | **1,500** |  | 0,75 | 1,75 | **1,625** |  |
| 48 | aprile 1999 | **RAE** | 1,50 | 0,75 | **1,875** |  | 0,75 | 1,75 | **1,625** |  |
| 48 | maggio 2000 | **RAE** | 1,50 | 0,75 | **1,875** |  | 0,75 | 1,75 | **1,625** |  |
| 48 | luglio 2001 | **RAE** | 2,00 | 0,75 | **2,375** |  | 1,25 | 1,75 | **2,125** |  |
| 48 | maggio 2002 | **RAE** | 1,75 | 1,00 | **2,250** |  | 1,25 | 1,75 | **2,125** |  |
| 48 | luglio 2003 | **RAE** | 1,75 | 1,00 | **2,250** |  | 1,50 | 1,75 | **2,375** |  |
| 48 | gennaio 2004 | **RAE** | 1,75 | 1,00 | **2,250** |  | 1,50 | 1,75 | **2,375** |  |
| 48 | aprile 2005 | **RAE** | 1,75 | 1,00 | **2,250** |  | 1,50 | 1,75 | **2,375** |  |
| 48 | maggio 2006 | **RAE** | 1,75 | 1,00 | **2,250** |  | 1,25 | 1,75 | **2,125** |  |
| 48 | settembre 2007 | **RAE** | 1,75 | 1,00 | **2,250** |  | 1,25 | 1,75 | **2,125** |  |
| 48 | aprile 2008 | **RAE** | 1,75 | 1,00 | **2,250** |  | 1,25 | 1,75 | **2,125** |  |
| 48 | gennaio 2009 | **RAE** | 1,75 | 1,00 | **2,250** |  | 1,25 | 1,75 | **2,125** |  |
| 48 | aprile 2010 | **RAE** | 1,75 | 1,00 | **2,250** |  | 1,25 | 1,75 | **2,125** |  |
| 48 | maggio 2011 | **RAE** | 1,75 | 1,00 | **2,250** |  | 1,25 | 1,75 | **2,125** |  |
| 48 | aprile 2012 | **RAE** | 1,75 | 1,00 | **2,250** |  | 1,25 | 1,75 | **2,125** |  |
| 48 | maggio 2013 | **RAE** | 1,75 | 1,00 | **2,250** |  | 1,25 | 1,75 | **2,125** |  |
| 49 | marzo 1999 | **RAE** | 5,00 |  | **5,000** |  | 5,50 |  | **5,500** |  |
| 49 | ottobre 1999 | **RAE** | 5,00 |  | **5,000** |  | 5,50 |  | **5,500** |  |
| 49 | febbraio 2001 | **RAE** | 4,50 |  | **4,500** |  | 5,00 |  | **5,000** |  |
| 49 | luglio 2001 | **RAE** | 3,75 |  | **3,750** |  | 5,00 |  | **5,000** |  |
| 49 | ottobre 2002 | **RAE** | 3,00 |  | **3,000** |  | 4,75 |  | **4,750** |  |
| 49 | ottobre 2003 | **RAE** | 3,00 |  | **3,000** |  | 4,75 |  | **4,750** |  |
| 49 | novembre 2004 | **RAE** | 2,75 |  | **2,750** |  | 4,75 |  | **4,750** |  |
| 49 | ottobre 2005 | **RAE** | 2,25 |  | **2,250** |  | 4,25 |  | **4,250** |  |
| 49 | ottobre 2006 | **RAE** | 2,50 |  | **2,500** |  | 4,25 |  | **4,250** |  |
| 49 | ottobre 2007 | **RAE** | 2,50 |  | **2,500** |  | 4,25 |  | **4,250** |  |
| 49 | novembre 2008 | **RAE** | 2,50 |  | **2,500** |  | 4,25 |  | **4,250** |  |
| 49 | novembre 2009 | **RAE** | 2,00 |  | **2,000** |  | 3,50 |  | **3,500** |  |
| 49 | ottobre 2010 | **RAE** | 2,00 |  | **2,000** |  | 3,50 |  | **3,500** |  |
| 49 | ottobre 2011 | **RAE** | 1,50 |  | **1,500** |  | 2,50 |  | **2,500** |  |
| 49 | ottobre 2012 | **RAE** | 1,50 |  | **1,500** |  | 2,50 |  | **2,500** |  |
| 49 | novembre 2013 | **RAE** | 1,50 |  | **1,500** |  | 2,50 |  | **2,500** |  |
| 49 | novembre 2014 | **RAE** | 1,50 |  | **1,500** |  | 2,50 |  | **2,500** |  |
| 50 | giugno 2001 | **RAE** | 6,00 | 1,50 | **6,750** |  | 4,50 | 1,25 | **5,125** |  |
| 50 | aprile 2002 | **RAE** | 6,00 | 1,50 | **6,750** |  | 4,50 | 1,25 | **5,125** |  |
| 50 | dicembre 2002 | **RAE** | 6,00 | 1,50 | **6,750** |  | 4,50 | 1,25 | **5,125** |  |
| 50 | gennaio 2004 | **RAE** | 6,00 | 1,50 | **6,750** |  | 4,50 | 1,25 | **5,125** |  |
| 50 | dicembre 2004 | **RAE** | 6,00 | 1,50 | **6,750** |  | 4,50 | 1,25 | **5,125** |  |
| 50 | dicembre 2005 | **RAE** | 6,00 | 1,50 | **6,750** |  | 4,50 | 1,25 | **5,125** |  |
| 50 | giugno 2007 | **RAE** | 5,00 | 1,50 | **5,750** |  | 4,50 | 1,25 | **5,125** |  |
| 50 | marzo 2008 | **RAE** | 5,00 | 1,50 | **5,750** |  | 4,50 | 1,25 | **5,125** |  |
| 50 | dicembre 2008 | **RAE** | 4,00 | 1,25 | **4,625** |  | 4,00 | 1,00 | **4,500** |  |
| 50 | maggio 2010 | **RAE** | 3,25 | 1,00 | **3,750** |  | 3,00 | 0,75 | **3,375** |  |
| 50 | maggio 2011 | **RAE** | 3,25 | 1,00 | **3,750** |  | 3,00 | 0,75 | **3,375** |  |
| 50 | luglio 2012 | **RAE** | 2,50 | 1,00 | **3,000** |  | 2,50 | 0,75 | **2,875** |  |
| 50 | febbraio 2013 | **RAE** | 1,50 | 0,25 | **1,625** |  | 2,00 | 0,75 | **2,375** |  |
| 50 | dicembre 2013 | **RAE** | 1,50 | 0,25 | **1,625** |  | 2,00 | 0,75 | **2,375** |  |
| 50 | gennaio 2015 | **RAE** | 1,00 | 1,00 | **1,500** |  | 1,25 | 0,75 | **1,625** |  |
| 50 | dicembre 2015 | **RAE** | 1,00 | 1,00 | **1,500** |  | 1,25 | 0,75 | **1,625** |  |
| 50 | dicembre 2016 | **RAE** | 1,00 | 1,00 | **1,500** |  | 1,25 | 0,75 | **1,625** |  |
| 51 | ottobre 1998 | **NRAE** | 4,75 | 0,75 | **5,125** |  | 5,00 | 1,50 | **5,750** | * |
| 51 | luglio 1999 | **NRAE** | 4,75 | 0,75 | **5,125** |  | 5,00 | 1,50 | **5,750** | * |
| 51 | giugno 2000 | **NRAE** | 5,50 | 1,25 | **6,125** |  | 5,00 | 1,75 | **5,875** | * |
| 51 | ottobre 2001 | **NRAE** | 5,00 | 1,25 | **5,625** |  | 5,00 | 1,75 | **5,875** | * |
| 51 | settembre 2002 | **NRAE** | 5,00 | 1,25 | **5,625** |  | 5,00 | 1,75 | **5,875** | * |
| 51 | settembre 2003 | **NRAE** | 4,50 | 1,25 | **5,125** |  | 5,00 | 1,50 | **5,750** | * |
| 51 | settembre 2004 | **NRAE** | 3,50 | 1,25 | **4,125** |  | 4,00 | 1,50 | **4,750** | * |
| 51 | settembre 2005 | **NRAE** | 3,50 | 1,25 | **4,125** |  | 4,00 | 1,50 | **4,750** | * |
| 51 | settembre 2006 | **NRAE** | 3,50 | 1,00 | **4,000** |  | 3,50 | 1,25 | **4,125** | * |
| 51 | settembre 2007 | **NRAE** | 3,50 | 1,00 | **4,000** |  | 3,50 | 1,25 | **4,125** | * |
| 51 | settembre 2008 | **NRAE** | 3,50 | 1,00 | **4,000** |  | 3,50 | 1,25 | **4,125** | * |
| 51 | settembre 2009 | **NRAE** | 2,50 | 0,75 | **2,875** |  | 2,75 | 1,25 | **3,375** | * |
| 51 | settembre 2010 | **NRAE** | 2,50 | 0,75 | **2,875** |  | 2,25 | 1,25 | **2,875** | * |
| 51 | settembre 2011 | **NRAE** | 2,50 | 0,75 | **2,875** |  | 2,25 | 1,25 | **2,875** | * |
| 51 | settembre 2012 | **NRAE** | 2,50 | 0,75 | **2,875** |  | 2,25 | 1,25 | **2,875** | * |
| 51 | settembre 2013 | **NRAE** | 2,50 | 0,75 | **2,875** |  | 2,25 | 1,25 | **2,875** | * |
| 51 | settembre 2014 | **NRAE** | 2,50 | 0,75 | **2,875** |  | 2,25 | 1,25 | **2,875** | * |
| 52 | settembre 2000 | **NRAE** | 1,50 |  | **1,500** |  | 1,50 | 0,50 | **1,750** |  |
| 52 | ottobre 2001 | **NRAE** | 1,50 |  | **1,500** |  | 1,50 | 0,50 | **1,750** |  |
| 52 | giugno 2002 | **NRAE** | 1,50 |  | **1,500** |  | 1,50 | 0,50 | **1,750** |  |
| 52 | ottobre 2003 | **NRAE** | 1,50 |  | **1,500** |  | 1,50 | 0,50 | **1,750** |  |
| 52 | luglio 2004 | **NRAE** | 1,25 |  | **1,250** |  | 1,00 | 0,50 | **1,250** |  |
| 52 | ottobre 2005 | **NRAE** | 1,25 |  | **1,250** |  | 1,00 | 0,50 | **1,250** |  |
| 52 | luglio 2006 | **NRAE** | 1,25 |  | **1,250** |  | 1,00 | 0,50 | **1,250** |  |
| 52 | settembre 2007 | **NRAE** | 1,25 |  | **1,250** |  | 1,00 | 0,50 | **1,250** |  |
| 52 | novembre 2008 | **NRAE** | 1,25 |  | **1,250** |  | 1,00 | 0,50 | **1,250** |  |
| 52 | febbraio 2010 | **NRAE** | 1,50 |  | **1,500** |  | 1,25 | 0,50 | **1,500** |  |
| 52 | febbraio 2011 | **NRAE** | 1,50 |  | **1,500** |  | 0,50 | 0,50 | **0,750** |  |
| 52 | febbraio 2012 | **NRAE** | 1,50 |  | **1,500** |  | 0,50 | 0,50 | **0,750** |  |
| 52 | dicembre 2012 | **NRAE** | 1,50 |  | **1,500** |  | 0,50 | 0,50 | **0,750** |  |
| 52 | ottobre 2013 | **NRAE** | 1,25 |  | **1,250** |  | 0,50 | 0,25 | **0,625** |  |
| 52 | settembre 2014 | **NRAE** | 1,25 |  | **1,250** |  | 0,50 | 0,50 | **0,750** |  |
| 52 | novembre 2015 | **NRAE** | 1,25 |  | **1,250** |  | 0,50 | 0,50 | **0,750** |  |
| 52 | luglio 2016 | **NRAE** | 1,25 |  | **1,250** |  | 0,50 | 0,50 | **0,750** |  |
| 53 | agosto 2000 | **NRAE** | 3,00 | 0,50 | **3,250** |  | 3,00 | 0,50 | **3,250** |  |
| 53 | ottobre 2001 | **NRAE** | 3,50 | 0,50 | **3,750** |  | 3,50 | 0,50 | **3,750** |  |
| 53 | giugno 2002 | **NRAE** | 3,75 | 0,50 | **4,000** |  | 3,50 | 0,50 | **3,750** |  |
| 53 | ottobre 2003 | **NRAE** | 3,75 | 0,50 | **4,000** |  | 3,50 | 0,50 | **3,750** |  |
| 53 | luglio 2004 | **NRAE** | 3,75 | 0,50 | **4,000** |  | 3,50 | 0,50 | **3,750** |  |
| 53 | novembre 2005 | **NRAE** | 3,75 | 0,50 | **4,000** |  | 3,50 | 0,50 | **3,750** |  |
| 53 | luglio 2006 | **NRAE** | 4,00 | 0,50 | **4,250** |  | 4,00 | 0,50 | **4,250** |  |
| 53 | settembre 2007 | **NRAE** | 4,00 | 0,50 | **4,250** |  | 4,00 | 0,50 | **4,250** |  |
| 53 | novembre 2008 | **NRAE** | 4,00 | 0,50 | **4,250** |  | 4,00 | 0,50 | **4,250** |  |
| 53 | febbraio 2010 | **NRAE** | 3,50 | 0,50 | **3,750** |  | 4,00 | 0,50 | **4,250** |  |
| 53 | febbraio 2011 | **NRAE** | 3,50 | 0,50 | **3,750** |  | 4,00 | 0,50 | **4,250** |  |
| 53 | febbraio 2012 | **NRAE** | 3,50 | 0,50 | **3,750** |  | 4,00 | 0,50 | **4,250** |  |
| 53 | febbraio 2013 | **NRAE** | 3,25 | 0,50 | **3,500** |  | 3,50 | 0,75 | **3,875** |  |
| 53 | ottobre 2013 | **NRAE** | 3,25 | 0,50 | **3,500** |  | 3,50 | 0,75 | **3,875** |  |
| 53 | settembre 2014 | **NRAE** | 3,25 | 0,50 | **3,500** |  | 3,50 | 0,75 | **3,875** |  |
| 53 | luglio 2015 | **NRAE** | 3,25 | 0,50 | **3,500** |  | 3,50 | 0,75 | **3,875** |  |
| 53 | luglio 2016 | **NRAE** | 3,25 | 0,50 | **3,500** |  | 3,50 | 0,75 | **3,875** |  |
| 54 | marzo 1996 | **NRAE** | 3,00 | 0,50 | **3,250** |  | 3,50 | 0,25 | **3,625** |  |
| 54 | maggio 1997 | **NRAE** | 3,00 | 0,50 | **3,250** |  | 3,50 | 0,25 | **3,625** |  |
| 54 | febbraio 1998 | **NRAE** | 3,00 | 0,50 | **3,250** |  | 3,50 | 0,50 | **3,750** |  |
| 54 | marzo 1999 | **NRAE** | 3,50 | 0,50 | **3,750** |  | 3,50 | 0,25 | **3,625** |  |
| 54 | febbraio 2000 | **NRAE** | 3,50 | 0,50 | **3,750** |  | 3,50 | 0,25 | **3,625** |  |
| 54 | marzo 2001 | **NRAE** | 3,50 | 0,50 | **3,750** |  | 3,50 | 0,25 | **3,625** |  |
| 54 | giugno 2002 | **NRAE** | 3,50 | 0,50 | **3,750** |  | 3,50 | 0,25 | **3,625** |  |
| 54 | ottobre 2003 | **NRAE** | 3,50 | 0,50 | **3,750** |  | 3,50 | 0,25 | **3,625** |  |
| 54 | febbraio 2004 | **NRAE** | 3,50 | 0,50 | **3,750** |  | 3,50 | 0,50 | **3,750** |  |
| 54 | febbraio 2005 | **NRAE** | 3,50 | 0,50 | **3,750** |  | 3,50 | 0,25 | **3,625** |  |
| 54 | marzo 2006 | **NRAE** | 3,50 | 0,50 | **3,750** |  | 3,50 | 0,25 | **3,625** |  |
| 54 | dicembre 2006 | **NRAE** | 3,50 | 0,50 | **3,750** |  | 3,50 | 0,25 | **3,625** |  |
| 54 | dicembre 2007 | **NRAE** | 3,50 | 0,50 | **3,750** |  | 3,50 | 0,25 | **3,625** |  |
| 54 | novembre 2008 | **NRAE** | 3,50 | 0,50 | **3,750** |  | 3,50 | 0,25 | **3,625** |  |
| 54 | febbraio 2010 | **NRAE** | 3,50 | 0,50 | **3,750** |  | 3,50 | 0,25 | **3,625** |  |
| 54 | febbraio 2011 | **NRAE** | 3,00 | 0,50 | **3,250** |  | 3,00 | 0,25 | **3,125** |  |
| 54 | novembre 2011 | **NRAE** | 3,00 | 0,50 | **3,250** |  | 3,00 | 0,25 | **3,125** |  |
| 55 | gennaio 1997 | **RAE** | 5,75 | 0,75 | **6,125** |  | 6,00 | 0,50 | **6,250** |  |
| 55 | maggio 1998 | **RAE** | 6,25 | 0,50 | **6,500** |  | 6,50 | 0,50 | **6,750** |  |
| 55 | febbraio 1999 | **RAE** | 7,00 | 0,50 | **7,250** |  | 6,50 | 0,50 | **6,750** |  |
| 55 | dicembre 1999 | **RAE** | 7,00 | 0,75 | **7,375** |  | 6,50 | 0,50 | **6,750** |  |
| 55 | novembre 2000 | **RAE** | 7,00 | 0,75 | **7,375** |  | 6,50 | 0,50 | **6,750** |  |
| 55 | gennaio 2002 | **RAE** | 7,00 | 0,75 | **7,375** |  | 6,50 | 0,50 | **6,750** |  |
| 55 | dicembre 2002 | **RAE** | 7,00 | 0,75 | **7,375** |  | 6,50 | 0,50 | **6,750** |  |
| 55 | gennaio 2004 | **RAE** | 7,00 | 0,75 | **7,375** |  | 6,50 | 0,50 | **6,750** |  |
| 55 | aprile 2005 | **RAE** | 6,75 | 0,75 | **7,125** |  | 6,00 | 0,75 | **6,375** |  |
| 55 | aprile 2006 | **RAE** | 6,75 | 0,75 | **7,125** |  | 6,00 | 0,75 | **6,375** |  |
| 55 | settembre 2007 | **RAE** | 6,75 | 0,75 | **7,125** |  | 6,00 | 0,75 | **6,375** |  |
| 55 | dicembre 2007 | **RAE** | 6,75 | 0,75 | **7,125** |  | 6,00 | 0,75 | **6,375** |  |
| 55 | novembre 2008 | **RAE** | 6,75 | 0,75 | **7,125** |  | 6,00 | 0,75 | **6,375** |  |
| 55 | settembre 2010 | **RAE** | 6,75 | 0,75 | **7,125** |  | 6,00 | 0,75 | **6,375** |  |
| 55 | ottobre 2010 | **RAE** | 6,00 | 0,75 | **6,375** |  | 6,00 | 1,00 | **6,500** |  |
| 55 | ottobre 2011 | **RAE** | 6,00 | 0,75 | **6,375** |  | 6,00 | 1,00 | **6,500** |  |
| 55 | dicembre 2012 | **RAE** | 5,75 | 0,75 | **6,125** |  | 5,00 | 1,00 | **5,500** |  |
| 56 | gennaio 1994 | **PAE** | 6,50 | 1,50 | **7,250** | * | 6,00 | 1,50 | **6,750** |  |
| 56 | luglio 1995 | **PAE** | 6,50 | 1,50 | **7,250** | * | 6,00 | 1,50 | **6,750** |  |
| 56 | ottobre 1996 | **PAE** | 6,50 | 1,50 | **7,250** | * | 6,00 | 1,50 | **6,750** |  |
| 56 | maggio 1997 | **PAE** | 6,50 | 1,50 | **7,250** | * | 6,00 | 1,50 | **6,750** |  |
| 56 | giugno 1998 | **PAE** | 6,50 | 1,50 | **7,250** | * | 6,00 | 1,50 | **6,750** |  |
| 56 | febbraio 1999 | **PAE** | 6,00 | 1,50 | **6,750** | * | 5,50 | 1,25 | **6,125** |  |
| 56 | dicembre 1999 | **PAE** | 6,00 | 1,50 | **6,750** | * | 5,50 | 1,25 | **6,125** |  |
| 56 | febbraio 2001 | **PAE** | 6,00 | 1,50 | **6,750** | * | 5,50 | 1,25 | **6,125** |  |
| 56 | febbraio 2002 | **PAE** | 6,00 | 1,50 | **6,750** | * | 5,00 | 1,25 | **5,625** |  |
| 56 | ottobre 2003 | **PAE** | 6,00 | 1,50 | **6,750** | * | 3,50 | 1,25 | **4,125** |  |
| 56 | ottobre 2004 | **PAE** | 5,00 | 1,50 | **5,750** | * | 2,50 | 1,25 | **3,125** |  |
| 56 | marzo 2005 | **PAE** | 4,50 | 1,50 | **5,250** | * | 2,00 | 1,50 | **2,750** |  |
| 56 | giugno 2006 | **PAE** | 4,00 | 1,50 | **4,750** | * | 1,50 | 1,50 | **2,250** |  |
| 56 | dicembre 2006 | **PAE** | 3,00 | 1,50 | **3,750** | * | 1,50 | 1,50 | **2,250** |  |
| 56 | gennaio 2008 | **PAE** | 3,00 | 1,50 | **3,750** | * | 1,00 | 1,75 | **1,875** |  |
| 56 | gennaio 2009 | **PAE** | 3,00 | 1,50 | **3,750** | * | 1,00 | 1,50 | **1,750** |  |
| 56 | gennaio 2010 | **PAE** | 3,00 | 1,50 | **3,750** | * | 1,00 | 1,50 | **1,750** |  |
| 57 | ottobre 1997 | **RAE** | 4,50 | 0,25 | **4,625** |  | 2,00 | 0,75 | **2,375** |  |
| 57 | gennaio 1999 | **RAE** | 5,00 | 0,25 | **5,125** |  | 2,00 | 0,75 | **2,375** |  |
| 57 | settembre 1999 | **RAE** | 6,00 | 0,25 | **6,125** |  | 3,50 | 0,75 | **3,875** |  |
| 57 | ottobre 2000 | **RAE** | 6,00 | 0,25 | **6,125** |  | 4,50 | 0,75 | **4,875** |  |
| 57 | settembre 2001 | **RAE** | 6,25 | 0,25 | **6,375** |  | 4,75 | 0,75 | **5,125** |  |
| 57 | aprile 2002 | **RAE** | 6,25 | 0,25 | **6,375** |  | 4,75 | 0,75 | **5,125** |  |
| 57 | dicembre 2003 | **RAE** | 6,00 | 0,25 | **6,125** |  | 4,50 | 0,75 | **4,875** |  |
| 57 | gennaio 2005 | **RAE** | 6,00 | 0,25 | **6,125** |  | 4,50 | 0,75 | **4,875** |  |
| 57 | novembre 2005 | **RAE** | 6,00 | 0,25 | **6,125** |  | 4,50 | 0,75 | **4,875** |  |
| 57 | novembre 2006 | **RAE** | 6,00 | 0,25 | **6,125** |  | 4,50 | 0,75 | **4,875** |  |
| 57 | novembre 2007 | **RAE** | 6,00 | 0,25 | **6,125** |  | 4,50 | 0,75 | **4,875** |  |
| 57 | settembre 2008 | **RAE** | 6,00 | 0,25 | **6,125** |  | 4,50 | 0,75 | **4,875** |  |
| 57 | dicembre 2009 | **RAE** | 6,00 | 0,25 | **6,125** |  | 4,50 | 0,75 | **4,875** |  |
| 57 | settembre 2010 | **RAE** | 6,00 | 0,25 | **6,125** |  | 4,50 | 0,75 | **4,875** |  |
| 57 | novembre 2011 | **RAE** | 6,00 | 0,25 | **6,125** |  | 4,50 | 0,75 | **4,875** |  |
| 57 | ottobre 2012 | **RAE** | 6,00 | 0,25 | **6,125** |  | 4,50 | 0,75 | **4,875** |  |
| 57 | dicembre 2013 | **RAE** | 6,00 | 0,25 | **6,125** |  | 4,50 | 0,75 | **4,875** |  |
| 58 | luglio 1996 | **PAE** | 2,75 | 0,50 | **3,000** |  | 3,25 | 0,25 | **3,375** |  |
| 58 | gennaio 1997 | **PAE** | 2,75 | 0,50 | **3,000** |  | 3,25 | 0,25 | **3,375** |  |
| 58 | ottobre 1997 | **PAE** | 2,75 | 0,50 | **3,000** |  | 3,25 | 0,25 | **3,375** |  |
| 58 | settembre 1998 | **PAE** | 2,75 | 0,50 | **3,000** |  | 3,25 | 0,25 | **3,375** |  |
| 58 | settembre 1999 | **PAE** | 3,25 | 0,50 | **3,500** |  | 3,25 | 0,25 | **3,375** |  |
| 58 | febbraio 2001 | **PAE** | 4,00 | 0,50 | **4,250** |  | 4,00 | 0,25 | **4,125** |  |
| 58 | luglio 2002 | **PAE** | 4,50 | 0,50 | **4,750** |  | 4,75 | 0,25 | **4,875** |  |
| 58 | gennaio 2003 | **PAE** | 4,50 | 0,50 | **4,750** |  | 4,75 | 0,25 | **4,875** |  |
| 58 | novembre 2003 | **PAE** | 4,50 | 0,50 | **4,750** |  | 4,75 | 0,25 | **4,875** |  |
| 58 | gennaio 2005 | **PAE** | 4,50 | 0,50 | **4,750** |  | 4,75 | 0,25 | **4,875** |  |
| 58 | gennaio 2006 | **PAE** | 4,50 | 0,50 | **4,750** |  | 4,75 | 0,25 | **4,875** |  |
| 58 | maggio 2007 | **PAE** | 4,50 | 0,50 | **4,750** |  | 4,00 | 0,25 | **4,125** |  |
| 58 | gennaio 2008 | **PAE** | 4,50 | 0,50 | **4,750** |  | 4,00 | 0,25 | **4,125** |  |
| 58 | marzo 2009 | **PAE** | 4,50 | 0,50 | **4,750** |  | 4,00 | 0,25 | **4,125** |  |
| 58 | febbraio 2010 | **PAE** | 4,50 | 0,50 | **4,750** |  | 4,00 | 0,25 | **4,125** |  |
| 58 | novembre 2010 | **PAE** | 4,50 | 0,50 | **4,750** |  | 4,00 | 0,25 | **4,125** |  |
| 58 | maggio 2012 | **PAE** | 4,50 | 0,50 | **4,750** |  | 4,00 | 0,25 | **4,125** |  |
| 59 | maggio 1999 | **PAE** | 6,00 | 2,50 | **7,250** |  | 6,25 | 2,50 | **7,500** |  |
| 59 | marzo 2000 | **PAE** | 6,00 | 2,50 | **7,250** |  | 6,00 | 2,50 | **7,250** |  |
| 59 | aprile 2001 | **PAE** | 6,00 | 2,50 | **7,250** |  | 6,00 | 2,50 | **7,250** |  |
| 59 | novembre 2002 | **PAE** | 6,00 | 2,50 | **7,250** |  | 6,00 | 2,50 | **7,250** |  |
| 59 | febbraio 2004 | **PAE** | 6,00 | 2,50 | **7,250** |  | 6,00 | 2,50 | **7,250** |  |
| 59 | luglio 2004 | **PAE** | 6,00 | 2,50 | **7,250** |  | 5,50 | 2,50 | **6,750** |  |
| 59 | dicembre 2005 | **PAE** | 6,00 | 2,50 | **7,250** |  | 5,50 | 2,50 | **6,750** |  |
| 59 | aprile 2006 | **PAE** | 6,00 | 2,50 | **7,250** |  | 5,50 | 2,50 | **6,750** |  |
| 59 | novembre 2007 | **PAE** | 6,00 | 2,50 | **7,250** |  | 5,50 | 2,50 | **6,750** |  |
| 59 | luglio 2008 | **PAE** | 6,00 | 2,50 | **7,250** |  | 5,50 | 2,50 | **6,750** |  |
| 59 | luglio 2009 | **PAE** | 6,00 | 2,50 | **7,250** |  | 5,50 | 2,50 | **6,750** |  |
| 59 | maggio 2010 | **PAE** | 6,00 | 2,50 | **7,250** |  | 5,50 | 2,50 | **6,750** |  |
| 59 | maggio 2011 | **PAE** | 6,00 | 2,50 | **7,250** |  | 5,50 | 2,50 | **6,750** |  |
| 59 | marzo 2012 | **PAE** | 5,50 | 2,50 | **6,750** |  | 5,50 | 2,50 | **6,750** |  |
| 59 | aprile 2013 | **PAE** | 5,00 | 2,50 | **6,250** |  | 5,00 | 2,50 | **6,250** |  |
| 59 | settembre 2014 | **PAE** | 5,00 | 2,50 | **6,250** |  | 5,00 | 2,50 | **6,250** |  |
| 59 | maggio 2015 | **PAE** | 5,00 | 2,50 | **6,250** |  | 5,00 | 2,50 | **6,250** |  |
| 60 | febbraio 2000 | **NRAE** | 6,00 | 1,50 | **6,750** |  | 6,00 | 1,75 | **6,875** |  |
| 60 | aprile 2001 | **NRAE** | 6,25 | 1,50 | **7,000** |  | 6,00 | 1,75 | **6,875** |  |
| 60 | dicembre 2001 | **NRAE** | 6,25 | 1,50 | **7,000** |  | 6,00 | 1,75 | **6,875** |  |
| 60 | marzo 2003 | **NRAE** | 6,00 | 1,50 | **6,750** |  | 6,00 | 1,75 | **6,875** |  |
| 60 | giugno 2004 | **NRAE** | 6,00 | 1,50 | **6,750** |  | 6,00 | 1,75 | **6,875** |  |
| 60 | gennaio 2005 | **NRAE** | 6,00 | 1,50 | **6,750** |  | 6,00 | 1,75 | **6,875** |  |
| 60 | giugno 2006 | **NRAE** | 6,00 | 1,50 | **6,750** |  | 6,00 | 1,75 | **6,875** |  |
| 60 | marzo 2007 | **NRAE** | 6,00 | 1,50 | **6,750** |  | 6,00 | 1,75 | **6,875** |  |
| 60 | febbraio 2008 | **NRAE** | 6,25 | 1,50 | **7,000** |  | 6,25 | 1,75 | **7,125** |  |
| 60 | maggio 2009 | **NRAE** | 6,25 | 1,50 | **7,000** |  | 6,25 | 1,75 | **7,125** |  |
| 60 | ottobre 2009 | **NRAE** | 6,25 | 1,50 | **7,000** |  | 6,25 | 1,75 | **7,125** |  |
| 60 | luglio 2011 | **NRAE** | 6,25 | 1,50 | **7,000** |  | 6,25 | 1,75 | **7,125** |  |
| 60 | giugno 2012 | **NRAE** | 6,25 | 1,50 | **7,000** |  | 6,25 | 1,75 | **7,125** |  |
| 60 | gennaio 2013 | **NRAE** | 6,25 | 1,50 | **7,000** |  | 6,25 | 1,75 | **7,125** |  |
| 60 | febbraio 2014 | **NRAE** | 6,25 | 1,50 | **7,000** |  | 6,25 | 1,75 | **7,125** |  |
| 60 | maggio 2015 | **NRAE** | 6,25 | 1,50 | **7,000** |  | 6,25 | 1,75 | **7,125** |  |
| 60 | ottobre 2015 | **NRAE** | 6,25 | 1,50 | **7,000** |  | 6,25 | 1,75 | **7,125** |  |
| 61 | febbraio 1999 | **PAE** | 5,00 | 0,50 | **5,250** |  | 5,00 | 0,50 | **5,250** |  |
| 61 | novembre 1999 | **PAE** | 7,25 | 0,75 | **7,625** |  | 7,25 | 1,00 | **7,750** |  |
| 61 | novembre 2000 | **PAE** | 8,00 | 0,75 | **8,375** |  | 8,00 | 1,00 | **8,500** |  |
| 61 | ottobre 2002 | **PAE** | 8,00 | 1,25 | **8,625** |  | 8,00 | 1,00 | **8,500** |  |
| 61 | gennaio 2003 | **PAE** | 8,00 | 0,75 | **8,375** |  | 8,00 | 1,00 | **8,500** |  |
| 61 | novembre 2003 | **PAE** | 8,00 | 0,75 | **8,375** |  | 8,00 | 1,00 | **8,500** |  |
| 61 | aprile 2005 | **PAE** | 8,25 | 0,75 | **8,625** |  | 8,00 | 1,00 | **8,500** |  |
| 61 | giugno 2006 | **PAE** | 8,00 | 0,75 | **8,375** |  | 8,00 | 1,00 | **8,500** |  |
| 61 | novembre 2006 | **PAE** | 7,00 | 0,75 | **7,375** |  | 7,00 | 0,75 | **7,375** |  |
| 61 | gennaio 2008 | **PAE** | 7,00 | 0,75 | **7,375** |  | 7,00 | 0,75 | **7,375** |  |
| 61 | agosto 2009 | **PAE** | 7,00 | 0,75 | **7,375** |  | 7,00 | 0,75 | **7,375** |  |
| 61 | settembre 2010 | **PAE** | 7,00 | 0,75 | **7,375** |  | 7,00 | 0,75 | **7,375** |  |
| 61 | giugno 2011 | **PAE** | 7,00 | 0,75 | **7,375** |  | 7,00 | 0,75 | **7,375** |  |
| 61 | settembre 2012 | **PAE** | 7,00 | 0,75 | **7,375** |  | 7,00 | 0,75 | **7,375** |  |
| 61 | gennaio 2013 | **PAE** | 7,00 | 0,75 | **7,375** |  | 7,00 | 0,75 | **7,375** |  |
| 61 | maggio 2014 | **PAE** | 7,00 | 0,75 | **7,375** |  | 7,00 | 0,75 | **7,375** |  |
| 61 | luglio 2015 | **PAE** | 7,00 | 0,75 | **7,375** |  | 7,00 | 0,75 | **7,375** |  |
| 62 | marzo 1992 | **RAE** | 3,50 | 0,50 | **3,750** |  | 2,50 | 0,75 | **2,875** |  |
| 62 | aprile 1993 | **RAE** | 4,50 | 0,50 | **4,750** |  | 2,50 | 0,75 | **2,875** |  |
| 62 | settembre 1994 | **RAE** | 4,25 | 0,50 | **4,500** |  | 2,75 | 0,75 | **3,125** |  |
| 62 | giugno 1995 | **RAE** | 4,50 | 0,50 | **4,750** |  | 3,25 | 0,75 | **3,625** |  |
| 62 | marzo 1996 | **RAE** | 4,50 | 0,50 | **4,750** |  | 4,00 | 0,75 | **4,375** |  |
| 62 | aprile 1997 | **RAE** | 5,00 | 0,50 | **5,250** |  | 4,50 | 0,75 | **4,875** |  |
| 62 | aprile 1998 | **RAE** | 5,00 | 0,50 | **5,250** |  | 4,50 | 0,75 | **4,875** |  |
| 62 | aprile 1999 | **RAE** | 5,00 | 0,50 | **5,250** |  | 4,50 | 0,75 | **4,875** |  |
| 62 | maggio 2000 | **RAE** | 4,50 | 0,75 | **4,875** |  | 4,50 | 0,75 | **4,875** |  |
| 62 | dicembre 2001 | **RAE** | 4,50 | 0,75 | **4,875** |  | 4,50 | 0,75 | **4,875** |  |
| 62 | febbraio 2002 | **RAE** | 4,50 | 0,75 | **4,875** |  | 4,50 | 0,75 | **4,875** |  |
| 62 | settembre 2003 | **RAE** | 4,00 | 0,75 | **4,375** |  | 4,00 | 0,75 | **4,375** |  |
| 62 | luglio 2004 | **RAE** | 4,00 | 0,75 | **4,375** |  | 4,00 | 0,75 | **4,375** |  |
| 62 | novembre 2005 | **RAE** | 3,50 | 0,75 | **3,875** |  | 3,50 | 0,75 | **3,875** |  |
| 62 | ottobre 2006 | **RAE** | 3,50 | 0,75 | **3,875** |  | 3,50 | 0,75 | **3,875** |  |
| 62 | aprile 2007 | **RAE** | 3,50 | 0,75 | **3,875** |  | 3,50 | 0,75 | **3,875** |  |
| 62 | settembre 2008 | **RAE** | 3,50 | 0,75 | **3,875** |  | 3,50 | 0,75 | **3,875** |  |
| 63 | marzo 1995 | **RAE** | 8,25 | 1,00 | **8,750** |  | 8,00 | 0,75 | **8,375** |  |
| 63 | luglio 1995 | **RAE** | 8,25 | 1,00 | **8,750** |  | 8,00 | 0,75 | **8,375** |  |
| 63 | gennaio 1997 | **RAE** | 8,25 | 1,00 | **8,750** |  | 8,00 | 0,75 | **8,375** |  |
| 63 | gennaio 1998 | **RAE** | 8,50 | 1,00 | **9,000** |  | 8,00 | 0,75 | **8,375** |  |
| 63 | febbraio 1999 | **RAE** | 8,50 | 1,00 | **9,000** |  | 8,25 | 0,75 | **8,625** |  |
| 63 | luglio 1999 | **RAE** | 8,50 | 1,00 | **9,000** |  | 8,00 | 0,75 | **8,375** |  |
| 63 | giugno 2000 | **RAE** | 8,50 | 1,00 | **9,000** |  | 8,00 | 0,75 | **8,375** |  |
| 63 | maggio 2001 | **RAE** | 8,50 | 1,00 | **9,000** |  | 8,00 | 0,75 | **8,375** |  |
| 63 | giugno 2002 | **RAE** | 8,50 | 1,00 | **9,000** |  | 8,25 | 0,75 | **8,625** |  |
| 63 | settembre 2003 | **RAE** | 8,00 | 1,25 | **8,625** |  | 8,25 | 0,75 | **8,625** |  |
| 63 | maggio 2004 | **RAE** | 7,25 | 1,25 | **7,875** |  | 7,25 | 0,75 | **7,625** |  |
| 63 | maggio 2005 | **RAE** | 6,50 | 1,50 | **7,250** |  | 7,25 | 0,75 | **7,625** |  |
| 63 | luglio 2006 | **RAE** | 6,50 | 1,50 | **7,250** |  | 7,25 | 0,75 | **7,625** |  |
| 63 | luglio 2007 | **RAE** | 6,50 | 1,50 | **7,250** |  | 7,25 | 0,75 | **7,625** |  |
| 63 | ottobre 2008 | **RAE** | 7,00 | 1,25 | **7,625** |  | 7,25 | 0,75 | **7,625** |  |
| 63 | gennaio 2010 | **RAE** | 7,00 | 1,25 | **7,625** |  | 7,25 | 0,75 | **7,625** |  |
| 63 | gennaio 2011 | **RAE** | 7,00 | 1,25 | **7,625** |  | 7,25 | 0,75 | **7,625** |  |
| 64 | febbraio 1998 | **PAE** | 3,00 | 0,50 | **3,250** | * | 3,00 | 1,25 | **3,625** |  |
| 64 | luglio 1998 | **PAE** | 4,00 | 0,50 | **4,250** | * | 4,00 | 1,25 | **4,625** |  |
| 64 | ottobre 1999 | **PAE** | 5,50 | 0,50 | **5,750** | * | 4,50 | 1,25 | **5,125** |  |
| 64 | novembre 2000 | **PAE** | 5,50 | 0,50 | **5,750** | * | 4,50 | 1,25 | **5,125** |  |
| 64 | luglio 2001 | **PAE** | 5,50 | 0,50 | **5,750** | * | 4,50 | 1,25 | **5,125** |  |
| 64 | settembre 2002 | **PAE** | 5,00 | 0,50 | **5,250** | * | 4,50 | 1,25 | **5,125** |  |
| 64 | luglio 2003 | **PAE** | 5,50 | 0,50 | **5,750** | * | 5,00 | 1,50 | **5,750** |  |
| 64 | luglio 2004 | **PAE** | 5,00 | 0,75 | **5,375** | * | 4,00 | 1,75 | **4,875** |  |
| 64 | ottobre 2005 | **PAE** | 5,00 | 0,50 | **5,250** | * | 4,00 | 1,50 | **4,750** |  |
| 64 | marzo 2007 | **PAE** | 5,00 | 0,50 | **5,250** | * | 4,00 | 1,50 | **4,750** |  |
| 64 | febbraio 2008 | **PAE** | 5,00 | 0,50 | **5,250** | * | 4,00 | 1,50 | **4,750** |  |
| 64 | novembre 2008 | **PAE** | 4,00 | 0,50 | **4,250** | * | 4,00 | 1,50 | **4,750** |  |
| 64 | marzo 2010 | **PAE** | 4,00 | 0,50 | **4,250** | * | 4,00 | 1,50 | **4,750** |  |
| 64 | gennaio 2011 | **PAE** | 4,00 | 0,50 | **4,250** | * | 4,00 | 1,50 | **4,750** |  |
| 64 | marzo 2012 | **PAE** | 3,50 | 0,50 | **3,750** | * | 4,00 | 1,50 | **4,750** |  |
| 64 | febbraio 2013 | **PAE** | 3,50 | 0,50 | **3,750** | * | 3,25 | 1,50 | **4,000** |  |
| 64 | luglio 2013 | **PAE** | 3,50 | 0,50 | **3,750** | * | 3,00 | 1,50 | **3,750** |  |
| 65 | novembre 2000 | **RAE** | 7,50 | 1,75 | **8,375** |  | 7,50 | 1,00 | **8,000** |  |
| 65 | luglio 2001 | **RAE** | 7,50 | 1,75 | **8,375** |  | 7,50 | 1,00 | **8,000** |  |
| 65 | aprile 2002 | **RAE** | 7,50 | 2,00 | **8,500** |  | 7,50 | 1,75 | **8,375** |  |
| 65 | novembre 2003 | **RAE** | 7,50 | 2,00 | **8,500** |  | 7,50 | 1,75 | **8,375** |  |
| 65 | ottobre 2004 | **RAE** | 7,50 | 2,00 | **8,500** |  | 7,50 | 1,75 | **8,375** |  |
| 65 | novembre 2005 | **RAE** | 7,50 | 2,00 | **8,500** |  | 7,50 | 1,75 | **8,375** |  |
| 65 | febbraio 2007 | **RAE** | 7,00 | 2,00 | **8,000** |  | 7,00 | 1,75 | **7,875** |  |
| 65 | gennaio 2008 | **RAE** | 7,00 | 2,00 | **8,000** |  | 7,00 | 1,75 | **7,875** |  |
| 65 | febbraio 2009 | **RAE** | 7,00 | 2,00 | **8,000** |  | 7,00 | 1,75 | **7,875** |  |
| 65 | aprile 2009 | **RAE** | 7,00 | 2,00 | **8,000** |  | 7,00 | 1,75 | **7,875** |  |
| 65 | giugno 2010 | **RAE** | 6,50 | 1,75 | **7,375** |  | 6,50 | 1,50 | **7,250** |  |
| 65 | giugno 2011 | **RAE** | 6,50 | 1,75 | **7,375** |  | 6,50 | 1,50 | **7,250** |  |
| 65 | luglio 2012 | **RAE** | 5,75 | 1,75 | **6,625** |  | 6,00 | 1,50 | **6,750** |  |
| 65 | settembre 2013 | **RAE** | 5,75 | 1,75 | **6,625** |  | 5,50 | 1,50 | **6,250** |  |
| 65 | ottobre 2014 | **RAE** | 5,00 | 1,50 | **5,750** |  | 5,00 | 1,50 | **5,750** |  |
| 65 | maggio 2015 | **RAE** | 5,00 | 1,50 | **5,750** |  | 5,00 | 1,50 | **5,750** |  |
| 65 | ottobre 2016 | **RAE** | 5,00 | 1,50 | **5,750** |  | 5,00 | 1,50 | **5,750** |  |
| 66 | settembre 2000 | **NRAE** | 6,75 | 0,50 | **7,000** |  | 6,75 | 0,75 | **7,125** |  |
| 66 | settembre 2001 | **NRAE** | 6,75 | 0,50 | **7,000** |  | 6,75 | 0,75 | **7,125** |  |
| 66 | ottobre 2002 | **NRAE** | 6,75 | 0,50 | **7,000** |  | 6,75 | 0,75 | **7,125** |  |
| 66 | novembre 2003 | **NRAE** | 6,75 | 0,50 | **7,000** |  | 6,75 | 1,00 | **7,250** |  |
| 66 | ottobre 2004 | **NRAE** | 5,75 | 0,50 | **6,000** |  | 5,75 | 0,75 | **6,125** |  |
| 66 | ottobre 2005 | **NRAE** | 5,75 | 0,50 | **6,000** |  | 5,75 | 0,75 | **6,125** |  |
| 66 | novembre 2006 | **NRAE** | 5,75 | 0,50 | **6,000** |  | 5,75 | 0,75 | **6,125** |  |
| 66 | novembre 2007 | **NRAE** | 5,75 | 0,50 | **6,000** |  | 5,75 | 0,75 | **6,125** |  |
| 66 | febbraio 2009 | **NRAE** | 5,75 | 0,50 | **6,000** |  | 5,75 | 0,75 | **6,125** |  |
| 66 | febbraio 2010 | **NRAE** | 5,75 | 0,50 | **6,000** |  | 5,75 | 0,75 | **6,125** |  |
| 66 | luglio 2010 | **NRAE** | 5,75 | 0,50 | **6,000** |  | 5,75 | 0,75 | **6,125** |  |
| 66 | maggio 2011 | **NRAE** | 5,75 | 0,50 | **6,000** |  | 5,75 | 0,75 | **6,125** |  |
| 66 | luglio 2012 | **NRAE** | 5,75 | 0,50 | **6,000** |  | 5,75 | 0,75 | **6,125** |  |
| 66 | ottobre 2013 | **NRAE** | 5,75 | 0,50 | **6,000** |  | 5,75 | 0,75 | **6,125** |  |
| 66 | maggio 2014 | **NRAE** | 5,75 | 0,50 | **6,000** |  | 5,75 | 0,75 | **6,125** |  |
| 66 | luglio 2015 | **NRAE** | 5,75 | 0,50 | **6,000** |  | 5,75 | 0,75 | **6,125** |  |
| 66 | ottobre 2016 | **NRAE** | 5,75 | 0,50 | **6,000** |  | 5,75 | 0,75 | **6,125** |  |

*amblyopic eyes
