# Supplementary material for: Long-term development of refractive error in refractive, nonrefractive and partially accommodative esotropia
Source: PLoS One. 2018 Sep 24;13(9):e0204396. doi: 10.1371/journal.pone.0204396 (PMC6152953; doi:10.1371/journal.pone.0204396)
Supplement: S5 Table — (DOCX) [file pone.0204396.s005.docx]

**S5 Table.**

| **patients#** | **AC/A ratio** |
| --- | --- |
| **16** | 10 |
| **31** | 10 |
| **51** | 10 |
| **66** | 9 |
| **39** | 8 |
| **40** | 8 |
| **41** | 8 |
| **52** | 8 |
| **53** | 7 |
| **54** | 7 |
| **9** | 6,5 |
| **34** | 6,5 |
| **13** | 6,4 |
| **14** | 6 |
| **21** | 6 |
| **42** | 6 |
| **28** | 5,5 |
| **43** | 5,5 |
| **45** | 5,5 |
| **60** | 5,5 |
